# A sulfatide-centered ultra-high-resolution magnetic resonance MALDI imaging benchmark dataset for MS1-based lipid annotation tools

**DOI:** 10.1093/gigascience/giaf150

**Published:** 2025-12-09

**Authors:** Lars Gruber, Stefan Schmidt, Thomas Enzlein, Carsten Hopf

**Affiliations:** CeMOS Research and Transfer Center, Mass Spectrometry and Optical Spectroscopy, Technische Hochschule Mannheim, 68165 Mannheim, Germany; Medical Faculty, Heidelberg University, 69117 Heidelberg, Germany; CeMOS Research and Transfer Center, Mass Spectrometry and Optical Spectroscopy, Technische Hochschule Mannheim, 68165 Mannheim, Germany; CeMOS Research and Transfer Center, Mass Spectrometry and Optical Spectroscopy, Technische Hochschule Mannheim, 68165 Mannheim, Germany; CeMOS Research and Transfer Center, Mass Spectrometry and Optical Spectroscopy, Technische Hochschule Mannheim, 68165 Mannheim, Germany; Medical Faculty, Heidelberg University, 69117 Heidelberg, Germany; Mannheim Center for Translational Neuroscience (MCTN), Medical Faculty Mannheim, Heidelberg University, 68167 Mannheim, Germany

**Keywords:** MALDI mass spectrometry, MRMS, MALDI imaging, mass spectrometry imaging, metabolite annotation tools, mid-infrared imaging, isotope fine structure, lipidomics, metabolomics

## Abstract

**Background:**

Spatial omics techniques are indispensable for studying complex biological systems and for the discovery of spatial biomarkers. While several current matrix-assisted laser desorption/ionization mass spectrometry imaging (MSI) instruments are capable of localizing numerous metabolites at high spatial and spectral resolution, most MSI data are acquired at the MS1 level only. Assigning molecular identities based on MS1 data presents significant analytical and computational challenges, as the inherent limitations of MS1 data preclude confident annotations beyond the sum formula level.

**Results:**

To enable future advancements of computational lipid annotation tools, well-characterized benchmark—or ground-truth—datasets are crucial, which exceed the scope of synthetic data or data derived from mimetic tissue models. To this end, we provide 2 sulfatide-centered, biology-driven magnetic resonance MSI (MR-MSI) datasets at different mass resolving powers that characterize lipids in a mouse model of human metachromatic dystrophy. These data include an ultra-high-resolution (R ∼1,230,000) quantum cascade laser mid-infrared imaging-guided MR-MSI dataset that enables isotopic fine structure analysis and therefore enhances the level of confidence substantially. To highlight the usefulness of the data, we compared 118 manual sulfatide annotations with the number of decoy database-controlled sulfatide annotations performed in Metaspace (67 at a false discovery rate <10%).

**Conclusions:**

Overall, our datasets can be used to benchmark annotation algorithms, validate spatial biomarker discovery pipelines, and serve as a reference for future studies that explore sulfatide metabolism and its spatial regulation.

## Data Description

The absence of ground-truth datasets (i.e., prior knowledge of which metabolites/lipid are present or not in a tissue of interest and the nonavailability of corresponding datasets containing high-confidence annotations for a large number of metabolites/lipids) has been posing a major obstacle to computational advancements in mass spectrometry imaging (MSI) [1]. In particular, datasets that can challenge computational tools for molecular annotation will be crucial for rapid progress in the field [2, 3]. To this end, we generated reusable and widely applicable datasets comprising quadruplicates of spatially focused, high-resolution mass spectrometry imaging data (MS1 level) derived from kidneys of an arylsulfatase A–deficient (ARSA^−/−^) mouse, a well-known genetic model of human metachromatic leukodystrophy [4]. Specifically, we developed a workflow that leverages quantum cascade laser mid-infrared (QCL-MIR) imaging to guide MSI on a 7T Fourier transform ion cyclotron resonance (FT-ICR) magnetic resonance mass spectrometer (MRMS; Supplementary Figs. S1 and S2). The resulting MS1 data were interpreted in conjunction with precise reference annotations obtained for sulfatide glycosphingolipid species in defined kidney regions by on-tissue fragmentation-based lipid identification using imaging parallel reaction monitoring–parallel acquisition serial fragmentation (IPRM-PASEF) on an orthogonal trapped ion mobility spectrometry (TIMS) time-of-flight mass spectrometer [5]. Through combination of ultra-high-resolution (R ∼1,230,000) MR-MSI MS1 data with systematic MS2 data obtained on a different mass spectrometer, we are establishing a concept for generating such benchmark datasets. Four biological replicates and cross-modal validation against 4D-lipidomics TIMS-MS ensure data quality [5]. The ultra-high-resolution dataset was further intended to be complemented by a high-resolution dataset. All files and preprocessing scripts are publicly available, thus supporting benchmarking and integration within spatial omics analyses, as further demonstrated in this work using Metaspace-ML [6].

## Context

Matrix-assisted laser desorption/ionization (MALDI) MSI has evolved into an invaluable tool in spatial biology [7–9] that enables the label-free detection and statistically validated visualization of molecular distributions in tissues [10, 11]. However, achieving reliable bimolecular interpretation of the inherently complex spatial molecular patterns fundamentally depends on the availability of high-quality datasets featuring unambiguous molecular identifications. Such datasets are crucial for facilitating the discovery of spatial biomarkers and yielding insights into tissue function, pathology, and pharmacodynamic or therapeutic responses [12, 13].

Prompted by the instrumental limitations outlined, for instance, in the 4S paradigm [7], several specialized methodologies for subspace imaging have been developed to facilitate the generation of high-quality MSI datasets, including spatial sparse sampling strategies [14, 15] or guided approaches. The latter comprises mass-guided approaches, for example, single-cell imaging [16] or on-tissue MS2 [17], and imaging-guided approaches [18–22], including the recently developed QCL-MIR imaging-guided MSI workflow [5]. In general, these workflows have been introduced with the objective of enhancing overall throughput. Sometimes, acquisition time saved by restricting MSI to defined regions of interest (ROIs) is reallocated to alternative workflows that operate MSI with advanced instrumental settings for MS1 data. These alternative workflows can include adjustments to laser beam settings [23], increased transient durations in FT-ICR MSI, or optimized ramp times in TIMS-MSI, which improve spatial resolution, mass resolution, and ion mobility separation, respectively. Furthermore, imaging-based guidance methods can be combined with a sophisticated MSI technique for on-tissue MS2 that utilizes ion mobility-enhanced methods such as IPRM-PASEF [5, 17]. This integration substantially increased data quality by enhancing the confidence level for molecular identifications, all without the need for high-performance liquid chromatography (HPLC) separation and directly in the spatial context of the tissue [24]. Notably, the exploration of the chemical space of sulfatide isoforms in an ARSA^−/−^ mouse model enabled us to introduce a ground truth to the MSI field, since sulfatides are known to accumulate in distinct ROIs of kidney sections from these mice.

**Figure 1:**
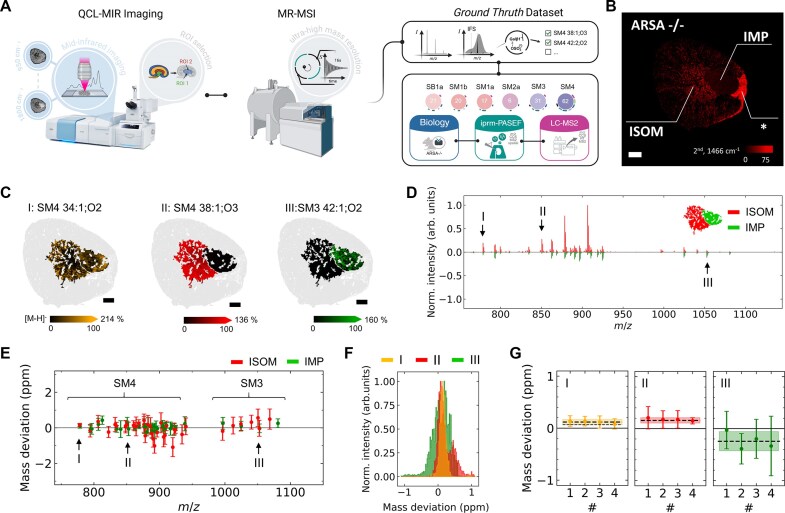
A sulfatide-centered, ultra-high-resolution QCL-MIR guided MSI benchmark dataset. (A) Schematic overview of data acquisition and incorporation of reference information. The methodology includes quantum cascade laser mid-infrared (QCL-MIR) imaging of kidney sections for region-focused magnetic resonance mass spectrometry imaging (MR-MSI) at ultra-long transient times (∼16 seconds), followed by manual sulfatide annotations based on a reference dataset validated at the MS2 level [5]. Created in BioRender. https://BioRender.com/to74o7c. (B) Representative lipid distribution in an ARSA^−/−^ kidney section based on the second derivative of absorbance at 1,466 cm^−1^ yielded predominant sulfatide accumulation in the ISOM region. The asterisk marks a region of high lipid content, as described in [5]. Scale bar: 500 µm. (C) Overlay of region-focused ion images of (I) *m/z* 778.5146 (SM4 34:1;O2[M-H]^−^; orange), (II) *m/z* 850.5721 (SM4 38:1;O3[M-H]^−^; red), and (III) *m/z* 1,052.6923 (SM3 42:1;O2[M-H]^−^; green) in kidney (gray). Mass window, ±3 ppm. (D) Representative butterfly plot of average mass spectra for the inner medulla/papilla (IMP; green) and ISOM (red) identified by QCL-MIR. (E) Mass deviation and uncertainties (standard deviation) for 47 sulfatides (signals present in at least 50 pixels of either region IMP or ISOM) at R_2_ ∼1.23 M. *m/z* values are shifted by +0.2 (IMP) or −0.2 (ISOM) for visualization. (F) Histogram of sum intensities in ISOM and IMP for (I), (II), and (III) measured with a mass resolution of ∼1.23 M at *m/z* 800. (G) Weighted mean mass deviation (dotted line) and uncertainty (*n* = 4) presented as internal error (filled area) for (I), (II), and (III). To this end, following Roux et al. [32], the larger value of the internal and extern error iwas taken as the final uncertainty to provide a conservative estimate. The shown results are consistent with the theoretical value within two standard deviations.

As our QCL-MIR imaging-based guidance approach is inherently instrument-agnostic, we have applied it in this study to create an ultra-high-resolution, sulfatide-focused MS1 benchmark dataset using a 7T XR FT-ICR (Supplementary Figs. S1 and S2). Using externally validated annotations, this dataset may become a unique benchmark resource (Fig. 1A) for developing and enhancing MS1 tools for deep spatial lipidomics and related research fields. This is especially important, because most MSI studies today still rely on acquiring only MS1 data [9, 25].

By default, annotations of MS1 data are limited to the sum formula level. For this and other reasons, the unambiguous annotation of sum formulae to MS1 data remains a nontrivial task, even with ultra-high mass resolving power (R >500.000) spectra, due to the sheer chemical diversity within biological samples [2, 26, 27]. Consequently, a single precise mass measurement may correspond to multiple candidate sum formulae, thereby complicating definitive assignment, even when the isotopic fine structures (IFSs) can be resolved. Nevertheless, the progressive exploitation of accurate mass, isotopic envelope, and IFS information [26–29] substantially increases the reliability of metabolite annotation in MR-MSI–based spatial omics studies, a process in which computational tools play a crucial role by enabling automated annotation workflows. For future advancement of such tools in MSI, well-characterized benchmark datasets with high mass accuracy will be essential to enable robust validation and method development. This highlights the necessity and reuse potential of the dataset introduced in this study.

## Methods

### QCL-MIR imaging of mouse kidneys

Animal studies involving ARSA^−/−^ mice and cryo-sectioning of kidneys have been described before [5]. To ultimately focus ultra-high-resolution MR-MSI data generation on defined kidney ROIs on an adjacent tissue section, we used QCL-MIR imaging for a prescan, followed by segmentation of hyperspectral QCL-MIR data to define ROIs [5]. These were then transferred to the MR-MSI instrument (Supplementary Figs. S1 and S2). To this end, QCL-MIR imaging data were recorded in sweep scan mode within a spectral range of 950–1800 cm^−1^ at a spectral sampling interval of 4 cm^−1^ on a Hyperion II ILIM (Bruker Optics) equipped with a 3.5× objective. Subsequently, ROIs were generated and selected using in-house software [30] based on spatial sulfatide distributions in ARSA^−/−^ mouse kidneys, which predominantly occur in the inner medulla/papillae (IMP) and inner stripe of outer medulla (ISOM). Specifically, these ROIs were then targeted for MR-MSI data acquisition with transient times of 15.7 seconds. All measurements were repeated for *n* = 4 biological replicates.

### Matrix spray-coating

A total of 10 mg/mL 2,5-dihydroxyacetophenone (DHAP) was dissolved in 70% acetonitrile (ACN) with 125 mM ammonium sulfate. After sonication, 0.1% trifluoroacetic acid (TFA) and 3 µM SM4 35:1;O2 (100 µg/mL [= 157.41 µM] in MeOH/chloroform 2:1) as internal standard (IS) were added. Matrix was applied with an M5 TM-Sprayer (HTX Technologies). Temperatures of the spray nozzle and tray were 75°C and 35°C, respectively. The spraying parameters were as follows: spray nozzle velocity, 1,200 mm/min; flow rate, 0.1 mL/min; number of passes, 10; track spacing, 2 mm; pattern, HH; pressure, 10 psi; gas low rate, 2 L/min; nozzle height, 40 mm; and drying time, 0 seconds.

### MRMS imaging data acquisition

Ultra-high-resolution MSI data were acquired on a solariX 7T XR FT-ICR MS (Bruker Daltonics), equipped with a smartbeam II 2-kHz laser and ftms control 2.3.0 software (Bruker Daltonics, Build 92). Mass spectra were acquired in negative ion mode (*m/z* range 401.29–2,600) and data acquisition size of 8 M, resulting in a free induction decay (FID) time of 15.7 seconds and a mass resolving power of 1,230,000 at *m/z* 800. Reducing the number of data points in the time domain to 512k resulted in an FID time of 0.98 seconds and a mass resolving power of R ∼77,000. Ion optics settings were constant for all measurements: funnel RF amplitude (150 Vpp), source octopole (5 MHz, 350 Vpp), and collision cell voltage: 1.5 V, cell: 2 MHz, 1,200 Vpp. The source DC optics were also constant for all measurements (capillary exit: −200 V, deflector plate: −220 V, funnel 1: −150 V, skimmer 1: −15 V), as well as the ParaCell parameters (transfer exit lens: 30 V, analyzer entrance: 10 V, sidekick: 0 V, side kick offset: 1.5 V, front/back trap plate: −3.4 V, back trap plate quench: 30 V). Sweet excitation power for ion detection was set to 14%, and ion accumulation time was 0.05 seconds. The transfer optics were as follows: time of flight, 1 ms; frequency, 4 MHz; and RF amplitude, 350 Vpp. The laser parameters were as follows: laser power, 32%; laser shots, 20; laser frequency, 200 Hz; and laser focus, medium, at a lateral step size of 40 µm.

### Mass spectrometry imaging data analysis

For state-of-the-art annotation with Metaspace [31], the v2 (Metaspace ML [6]) algorithm and an *m/z* tolerance of 2 ppm were utilized. The imzML files were exported from SCiLS Lab (Version 2024a Pro; Bruker Daltonics). A custom reference database integrating LipidMaps with 780 theoretically derived sulfatide structures, available as Supplementary Dataset 3, was constructed for Metaspace analysis. All sulfatide species manually identified in this study (91 annotations from the first replicate, 97 from the second replicate) were established constituents of the original database prior to manual annotation, thereby ensuring methodological independence and precluding database incompleteness as a confounding factor in the assessment of algorithm performance. For manual annotation, we compared the QCL-MIR–guided MR-MSI data against the ground-truth data from Gruber et al. [5]. For direct comparison of ultra-high-resolution and high-resolution datasets, MS-based data segmentation was employed to enable subspace modeling. Bisecting k-means clustering of the MSI data from whole-tissue kidney sections was performed via SCiLS Lab to delineate anatomical regions, specifically the ISOM and the IMP. These region outlines were subsequently used to generate a region-focused dataset, facilitating a more targeted comparative analysis.

## Materials

All chemicals and solvents were of HPLC-MS grade. Conductive indium tin oxide (ITO)–coated glass slides were purchased from Diamond Coatings. The MALDI matrix DHAP was purchased from Thermo Fisher Scientific. ACN, ethanol, liquid chromatography–mass spectrometry (LC-MS) water, 2-propanol, and ammonium sulfate were obtained from VWR Chemicals. The sulfatide standard C_17_ mono-sulfo galactosyl(β) ceramide d18:1/17:0 (SM4 35:1;O2) was purchased from Avanti Polar Lipids. TFA, Mayer’s hemalum solution, hydrochloric acid, sodium bicarbonate, magnesium sulfate, eosin Y-solution 0.5%, xylene, and eukitt were purchased from Merck KGaA.

## Data Validation and Quality Control

To enable the advancement of MS1-based molecular annotation tools for spatial biomarker discovery pipelines and other purposes, we acquired and in-depth–characterized 2 sulfatide-centered, biology-driven datasets derived from an arylsulfatase A mouse model at 2 different mass resolving powers. This includes an ultra-high mass resolution MR-MSI dataset (R ∼1,230,000) acquired using our recently introduced QCL-MIR guidance approach [5], in this study, combined with a 7T FT-ICR mass spectrometer. Briefly summarized, this workflow utilizes QCL-MIR imaging microscopy to rapidly acquire hyperspectral data from tissue samples, here, fresh-frozen kidney sections. Subsequent application of unsupervised segmentation algorithms, specifically k-means clustering, allows for the identification of biologically relevant ROIs within the kidney tissue, particularly IMP and ISOM, where sulfatides are enriched in ARSA^−/−^ mice. Ultra-high-resolution MR-MSI was then performed in a ROI-targeted manner on these 2 tissue morphologies within kidneys from two 12-week-old and two 60-week-old ARSA^−/−^ mice (Fig. 1A; Supplementary Figs. S1 and S2). The QCL-MIR imaging enabled focus on just 2 morphologies, enhancing both analytical depth and data acquisition efficiency (e.g., measurement time). As a reference and benchmark for the molecular content of these tissue areas and the total number of potential sulfatide identifications, we relied on our published reference data, which are based on 3 pillars: a known biological pathway leading to lipid class–specific accumulation of sulfatides, IPRM-PASEF–derived molecular identifications in conjunction with 4-dimensional lipidomics LC-MS data (Supplementary Table S1), and systematic MS/MS fragmentation validation [5]. Sulfatide structural identifications were systematically validated through on-tissue parallel reaction monitoring with ion mobility separation. Diagnostic fragment ions specific to each sulfatide subclass provide an unambiguous biochemical chain of evidence. These include neutral loss of the α-hydroxylated fatty acid (α-OH-FA), fragment for a phytosphingoid backbone (PSPB-Gal-SO₃⁻), and characteristic sulfate-containing headgroup fragments: GalNAc-Gal-SO₃⁻, Glc-Gal-SO₃⁻, and RCF-Gal-SO₃⁻ for the respective sulfatide species (SM4, SM3, SM2a, and SB1a). Common fragments, such as Gal-SO₃⁻ and HSO_4_⁻, appear across all sulfatide subclasses and serve as internal validation markers. Representative MS/MS spectra from sulfatide standards demonstrated high consistency (cosine similarity ≥0.99) across replicates, confirming the reliability and reproducibility of these fragmentation patterns. All manual annotations in this benchmark dataset were assigned only after successful MS/MS validation within the reference dataset, ensuring maximal confidence for downstream benchmarking studies.

**Figure 2:**
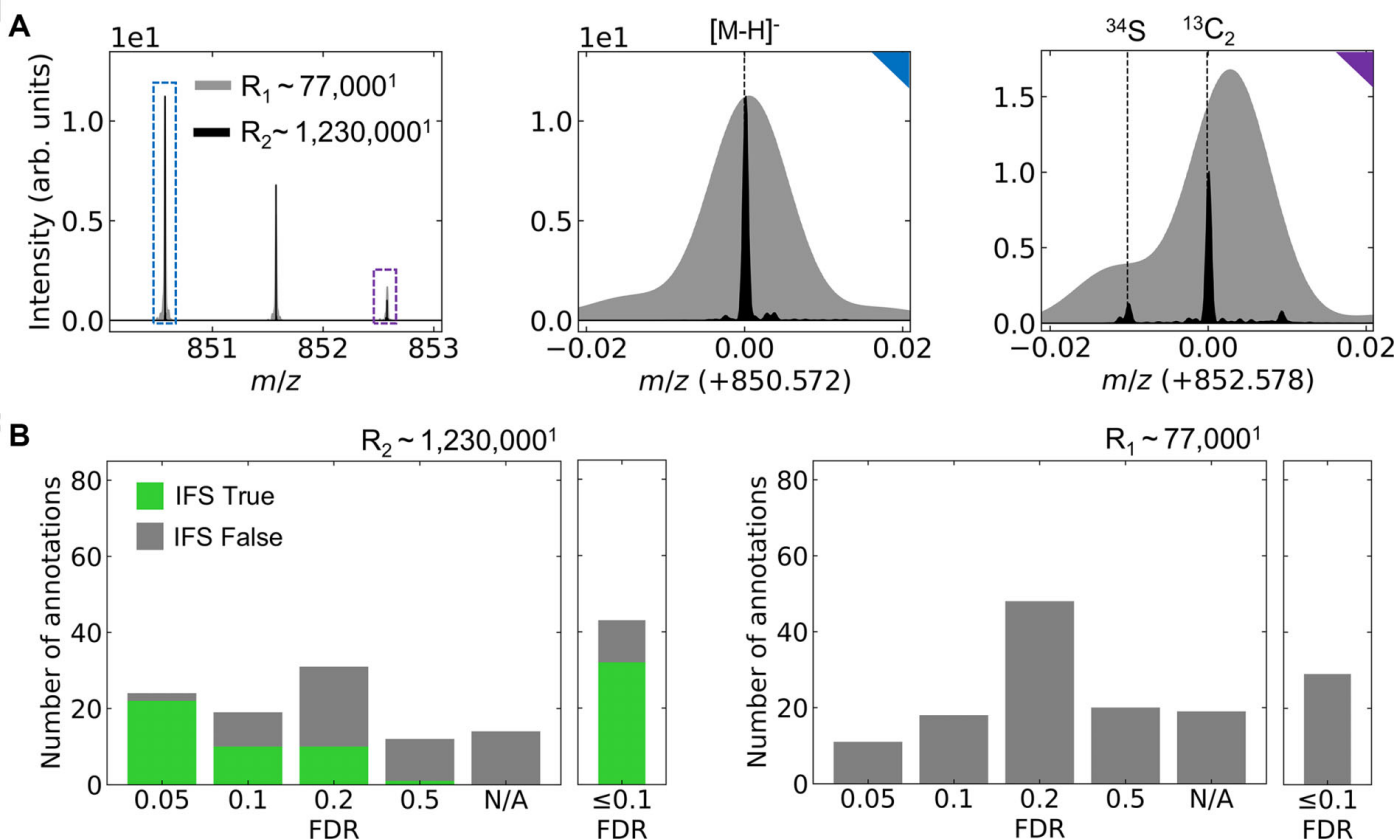
Evaluation of the annotation quality for the sulfatide-centered QCL-MIR–guided MSI dataset. (A) Ultra-high-resolution MR-MSI data were acquired with a mass resolution of R_1_ ∼77,000 (gray) and R_2_ ∼1,230,000 (black). Isotopic fine structure (IFS) of SM4 38:1;O3[M-H]^−^ incl. ^13^C_2_ (M+2) and ^34^S isotopic peaks, normalized to the monoisotopic peak. (B) Sulfatide annotations were performed using Metaspace with an in-house database containing 780 theoretical sulfatides compiled from LipidMaps. The ultra-high-resolution dataset (R_2_ ∼1,230,000, left) yielded substantially higher annotation rates than the standard resolution dataset (R_1_ ∼77,000, right), demonstrating the superior discriminatory power of ultra-high mass resolving power for sulfatide characterization. At FDR ≤10%, the ultra-high-resolution dataset identified 43 total sulfatides (32 with IFS confirmation, 11 without), compared to only 29 annotations at the same FDR threshold for the standard resolution dataset (which yielded 11 annotations at the more stringent FDR ≤5%). ^1^Mass resolving power at *m/z* 800. N/A marks annotations that were performed manually (and validated via *in situ* MS/MS and/or LC-MS/MS) but were not annotated by Metaspace at any FDR level.

We compared conventional MR-MSI of whole kidney slices at a mass resolution of R_1_ ∼77,000 at *m/z* 800 (1-second FID time; 14,331 pixels, 40 × 40 µm² pixel size; 5 hours of data acquisition) in the FT-ICR with QCL-MIR–guided analysis (Fig. 1B) focused on the ISOM and IMP ROIs, which achieved R_2_ ∼1,230,000 at *m/z* 800 (16-second FID time; 2,672 pixels, 40 × 40 µm² pixel size; 11.6 hours of data acquisition), resulting in a 16-fold increase in mass resolving power (Supplementary Fig. S3). For the QCL-MIR–guided MR-MSI dataset, 3 sulfatide ion images are presented as examples that displayed similar intensities in both ROIs (**I**, *m/z* 778.5146 [SM4 34:1;O2[M-H]^−^]), higher intensity in ISOM (**II**, *m/z* 850.5721 [SM4 38:1;O3[M-H]^−^]), or higher intensity in IMP (**III**, *m/z* 1,052.6923 [SM3 42:1;O2[M-H]^−^]). Unique molecular fingerprints were obtained per region (Fig. 1D). In both datasets, the mass deviation was constant across the *m/z* range (750–1,100) and was consistently below 2 ppm, even when considering the uncertainties across *n* = 4 biological replicates (Fig. 2E; Supplementary Fig. S4). The maximum mass deviation was about 1 ppm for the 2 less intense sulfatide ions **(I)** and (**III)** and less than 0.2 ppm for ion **(II)** at R_1_ ∼77,000, improving to below 0.2 ppm for (**I**) and (**II**) (around 0.5 ppm for (**III**)) with a mass resolution of R_2_ ∼1,230,000 (Fig. 1F; Supplementary Fig. S4). The reproducibility of our data is emphasized by comparing the mass deviation across *n* = 4 biological replicates for ions (**I**)–(**III**), all of which showed values below 1 ppm (Fig. 1G). Overall, the uncertainty of *m/z* values was reduced by a factor of 3–6 for data acquired at ultra-high-resolution on an FT-ICR instrument.

The ultra-high mass resolving power applied to the QCL-MIR dataset enables the detection of IFS for the sulfatides. The IFS, particularly the peak attributed to ^34^S, was very well resolved, with a signal-to-noise ratio of approximately 10 times the full width at half maximum (FWHM) (Fig. 2A; Supplementary Fig. S5; Supplementary Dataset 2). Nevertheless, it is important to note that in all cases where FID times are notably prolonged, it is necessary to operate with a reduced total ion current. This precautionary measure is pivotal to reduce (local) space charge effects [33–35] within the ion cyclotron resonance (ICR) cell (Supplementary Fig. S6). However, this results in a loss of sensitivity, which in turn leads to slightly reduced numbers of sulfatide annotations, particularly for those isoforms that have a comparatively lower concentration in IMP and ISOM than in the cortex. Overall, the number of candidate sulfatide identifications using the QCL-MIR imaging-guided ultra-high-mass resolution MR-MSI method was 91 and 97 for the two 60-week ARSA^−/−^ mice, compared to 118 and 115 annotations obtained with the conventional whole-tissue method (Table 1). The identification confidence was, on the other hand, dramatically improved, as 34 and 39 ultra-high mass resolution spectra were supported by IFS information (Table 1). Identification was performed manually, as IFS is currently not utilized adequately in commercial sum formula annotation tools, which match experimental against theoretical isotope patterns [36]. In the sulfatide case, the ^34^S isotope peak was not automatically recognized as such, but predefinition of S and N as constituents of the molecule of interest in the Bruker Smart Formula tool led to successful searches.

**Table 1:** Cumulative numbers of sulfatide subclass isoforms identified in ARSA^−/−^ mouse kidney by QCL-MIR imaging-guided MR-MSI. Whole kidney sections of 12- or 60-week-old ARSA^−/−^ mice (*n* = 2 each) were analyzed by conventional nonguided MR-MSI with a mass resolution of R_1_ ∼77k (at *m/z* 800) and QCL-MIR imaging-guided MR-MSI with a mass resolution of R_2_ ∼1,230k (at *m/z* 800). In many cases of QCL-MIR imaging-guided MSI, isotope fine structures (IFS) could be used for added confidence.

			SM4	SM3	SM2a	SB1a	Total
**R_1_, FID 1 second**	Total annotations in ROI	60w_1	56	37	6	19	118
		60w_2	54	36	6	19	115
		12w_1	45	36	6	16	103
		12w_2	52	36	6	18	112
**R_2_, FID 16 seconds**	Total annotations in ROI	60w_1	54	25	0	12	91
		60w_2	54	27	4	12	97
		12w_1	42	16	1	8	67
		12w_2	52	19	1	5	77
	Annotations with confirmed IFS evidence	60w_1	25	7	0	2	34
		60w_2	27	8	0	4	39
		12w_1	17	4	0	1	22
		12w_2	20	6	0	0	26

To highlight the potential reuse of our dataset as a benchmark for cutting-edge MALDI MSI data annotation, we employed the open-source Metaspace platform [37] to compare sulfatide annotations identified across varying false discovery rate (FDR) thresholds. FDR-based quality measures have recently been critically assessed in the more mature field of proteomics [38], as (i) FDR determination is often a black box, and (ii) it is often unclear at what level FDR is set. For MSI, the FDR-control process in Metaspace is fairly transparent [31]. Given the limited coverage of sulfatides in publicly available databases, we curated a custom database integrating LipidMaps entries with 780 theoretically derived sulfatide structures (Supplementary Dataset 3). For data acquired at 77,000 mass resolving power, IMP and ISOM were extracted using MS feature-based segmentation to maintain consistency in the analysis.

Comparison of our manual annotations with Metaspace’s database-controlled annotations at different FDR thresholds reveals both the effectiveness of current annotation algorithms and specific limitations when applied to ultra-high-resolution FT-ICR MSI datasets. The superior performance of ultra-high mass resolving power is evident across all FDR stringency levels. At FDR ≤5%, the ultra-high-resolution dataset (1,230,000 R) yielded 24 sulfatide annotations (22 supported by IFS), whereas the standard resolution dataset (77,000 R) identified only 11 annotations (Fig. 2B). At a 10% FDR threshold, this performance gap widened substantially: Metaspace identified 43 sulfatides from the ultra-high-resolution dataset (32 confirmed by IFS peaks), compared to only 29 annotations at standard resolution, demonstrating robust algorithmic performance and the critical importance of mass resolving power for sulfatide characterization. The number of annotations continued to increase with more permissive FDR thresholds, reaching 74 sulfatides (42 supported by IFS) at FDR ≥20% for the ultra-high-resolution dataset and 77 for the standard resolution dataset, although the relative proportion supported by IFS declined with increasing FDR stringency.

However, 14 sulfatides remained unannotated (N/A in Fig. 2B), even at a permissive ≥50% FDR threshold. These unannotated species represent low-abundance compounds for which only the monoisotopic [M-H]⁻ peak exceeded the signal-to-noise threshold, while the complete isotopic envelope fell below detection limits (Supplementary Fig. S7). This represents a measurement constraint rather than an algorithmic deficiency: Metaspace appropriately requires resolvable isotopic envelope information for confident annotation and conservatively rejects *m/z* features with incomplete or missing isotopic patterns rather than generating spurious assignments. These missing annotations were recovered through manual monoisotopic *m/z* matching against the custom reference database and external validation [5].

Metaspace’s scoring method limits isotopic envelope matching to the 4 most intense peaks. While this approach successfully annotates species with sufficient abundance to resolve their complete isotopic patterns, it fails to exploit diagnostic IFS information when present above noise thresholds. Our analysis demonstrates that 37% of sulfatide identifications in the first replicate (34 of 91) and 40% in the second replicate (39 of 97) were confirmed by detecting ³^4^S IFS peaks. This represents an underutilized signal: high-confidence annotations substantiated by robust IFS peak detection remain indistinguishable from lower-confidence assignments based solely on precursor *m/z* and mass error tolerance within the current algorithmic framework.

Future annotation algorithms should be refined to leverage the full isotopic envelope in ultra-high-resolution datasets while explicitly accounting for signal intensity–dependent constraints on IFS detectability. Prospective algorithmic developments should incorporate (i) expansion of isotopic envelope matching to encompass all resolved peaks above instrument-specific signal-to-noise thresholds, superseding the current 4-peak limitation; (ii) implementation of confidence stratification metrics that explicitly correlate annotation reliability with precursor ion signal intensity and noise levels, acknowledging that minor isotopic constituents—such as ³^4^S with 4.5% relative abundance compared to the monoisotopic form (exemplified by SM4 38:1;O3 in Fig. 2A)—exhibit attenuated detectability at reduced precursor ion intensities, thereby imposing practical limitations on IFS-based structural discrimination for low-abundance species; (iii) integration of machine learning classification frameworks trained on empirically acquired IFS signatures from characterized standards, enabling recognition of subclass-specific isotopic patterns across heterogeneous signal intensity regimes; and (iv) incorporation of complementary analytical dimensions such as TIMS-FT-ICR (recently applied to lipid analysis) or MS/MS fragmentation patterns to adjudicate between isotopically indistinguishable molecular formulas when mass defect alone provides insufficient discriminatory power [39, 40]. While such hyphenated approaches show promise for structural discrimination, their integration into spatial lipidomics workflows remains under development [39, 40]. This benchmark dataset provides a validated ground truth required for development and rigorous evaluation of such algorithmic advances, establishing quantitative performance benchmarks for annotation tools in the ultra-high-resolution regime and reinforcing that expert curation remains indispensable despite high data quality achieved with contemporary instrumentation.

## Reuse Potential

This rigorously validated (see [5]) sulfatide-focused benchmark dataset offers significant reuse potential for the MSI community. Collected at 2 levels of mass resolving power, the dataset enables a thorough evaluation of annotation strategies, especially those that utilize IFS for high-confidence MS1-level metabolite identification. As shown, IFS analysis yields greater annotation accuracy than current automated platforms, highlighting the need for further methodological innovation in computational annotation. Although some studies describe ultra-high resolution [41, 42], none of these share their data under Findable, Accessible, Interoperable, and Reusable [FAIR, 43] principles, and no externally validated annotations are available. Notably, the ultra-high-resolution QCL-MIR–guided approach used here emphasizes annotation confidence through isotopic fine structure analysis and reduced ion current to minimize space-charge effects; however, this optimization involves trade-offs in sensitivity and spatial coverage compared to traditional whole-tissue MSI experiments. The number of sulfatides identified in the QCL-MIR–guided regions of interest (91 and 97 for the 2 replicates) was lower than in the corresponding unguided whole-tissue analysis (118 and 115), reflecting this methodological trade-off between analytical depth and comprehensive tissue coverage. This dataset is a valuable resource for benchmarking and validating spatial biomarker discovery pipelines, developing new annotation algorithms, and supporting future research into sulfatide metabolism and its spatial regulation in biological tissues. Its utility for both computational tool development and biological studies ensures ongoing relevance for advancing spatial metabolomics and related fields.

## Availability of Source Code and Requirements

All code used for data generation within this study was already published in the scope of [5].

Project name: QCL_MIR_guided_MSI

Project homepage: https://github.com/CeMOS-Mannheim/QCL_MIR_guided_MSI

License: MIT license

System requirements

Operating system: Windows 10 (or higher)

Programming language: Python v3.8

Package management: https://github.com/CeMOS-Mannheim/QCL_MIR_guided_MSI/blob/master/requirements.txt

Hardware requirements: 32 GB RAM and Intel Core i5-9600 K CPU @ 3.70 GHz 3.70 GHz Processor (or better)

## Additional Files

**Supplementary Dataset 1**. Evaluation of sulfatide peak analysis for conventional 7T XR FT-ICR data with a 1-second FID time.

**Supplementary Dataset 2**. Evaluation of sulfatide peak analysis for conventional 7T XR FT-ICR data with a 16-second FID time.

**Supplementary Dataset 3**. List of theoretical sulfatide configurations provided as a .xlsx file.

**Supplementary Dataset 4**. List of all annotated sulfatide configurations, including mass deviation and IFS indication, for each replicate provided as a .xlsx file.

**Supplementary Dataset 5**. List of all Metaspace sulfatide annotations for both mass resolving powers and each replicate provided as a .xlsx file.

**Supplementary Fig. S1**. Schematic overview of the QCL-MIR imaging guidance and MR-MSI data acquisition workflow. Tissues (here, kidneys from arylsulfatase A knockout mice) are cryosectioned and dried and scanned by quantum-cascade laser (QCL) mid-infrared (MIR) imaging microscopy. Hyperspectral datasets are segmented based on chemical composition of the tissue, and regions of interest (ROI) are transferred to an FT-ICR magnetic resonance mass spectrometry imaging (MR-MSI) instrument. The tissue section is spray-coated with a chemical MALDI matrix and subjected to MALDI MS imaging on the MR-MSI system at either high resolution (R ∼ 77,000) or ultra-high resolution (R ∼ 1,230,000).

**Supplementary Fig. S2**. Detailed schematic of the guidance and data acquisition workflow. (i) Schematic overview of the QCL microscope and the QCL-IRI-guided MSI workflow for fresh-frozen biological specimens in reflection mode. (ii) Key spectral features are selected using the second derivative of absorbance (1,466 cm⁻¹, CH₂ bending; 1,742 cm⁻¹, C=O stretch). (iii) A single wavenumber (1,656 cm⁻¹) image is acquired as a reference and coregistered with the QCL-MIR dataset-derived regions of interest (iv and v). (vi) MR-MSI is performed with a focus on these ROIs, enabling the detection of isotopic fine structures of sulfatides. A_ν_: absorbance; CR: coherence reduction.

**Supplementary Fig. S3**. Evolution of experimentally deducted FWHM for 2 different mass resolving powers across the lipid mass range for datasets of a 60-week-old mouse kidney. Full width at half maximum (FWHM) as a function of the *m/z* value for 2 different free induction decay (FID) times of 1 second (gray, mass resolving power R_1_) and 16 seconds (black, mass resolving power R_2_). The orange (1-second FID) and blue (16-second FID) curves result from a locally estimated scatterplot smoothing (loess) and are plotted to guide the eye. On average, the ratio of the FWHM with R_2_ to the FWHM with R_1_ agrees well with the expected mass resolving power determined by the relative duration of the FID times.

**Supplementary Fig. S4**. Mass deviation across the lipid mass range for R_1_ ∼77,000 across *n* = 4 biological replicates of ARSA^−/−^ mouse kidney. (a) Mean mass deviation for sulfatides identified by MR-MSI (solariX 7T XR) with a mass resolution of R_1_ ∼77,000 at *m/z* 800. Means of mass deviation and standard deviations for each sulfatide were obtained from the sum intensity histograms in b, which resulted from the mean *m/z* value (determined across all pixels) of a given sulfatide. (b) Histogram of the sum intensity for the 3 sulfatides I SM4 34:1;O2[M-H]^−^, II SM4 38:1;O3[M-H]^−^, and III SM3 42:1;O2[M-H]^−^.

**Supplementary Fig. S5**. Spatial distribution of 3 sulfatide species and their respective isotopic fine structure. Ultra-high-resolution MRMS data for (a) I *m/z* 778.5146 (SM4 34:1;O2[M-H]^−^), (b) II *m/z* 850.5721 (SM4 38:1;O3[M-H]^−^) , and (c) III *m/z* 1,052.6923 (SM3 42:1;O2[M-H]^−^) acquired with a mass resolution of R_2_ ∼1,230,000 using QCL-MIR imaging-guided MR-MSI. Positions of the isotopic fine structure (IFS) peak, including ^13^C_2_ (M+2) and ^34^S, are highlighted by dotted lines, respectively. Respective data for all other sulfatides are presented in Supplementary Dataset 2. Pixel size, 40 µm.

**Supplementary Fig. S6**. Observation of space-charge effects for 3 sulfatide examples. Evaluation for (a) *m/z* 850.5720 (SM4 38:1;O3[M-H]^−^), (b) *m/z* 878.6033 (SM4 40:1;O3[M-H]^−^), and (c) *m/z* 890.6397 (SM4 42:1;O2[M-H]^−^). All data were acquired with a mass resolution of R_2_ ∼1,230,000 using QCL-MIR imaging-guided MR-MSI. Bin width for histogram, 0.05 ppm.

**Supplementary Fig. S7**. Exemplar of a missing annotation (N/A case): SM4 34:0;O4 with undetectable isotopic fine structure. (a) Spatial distribution and mass spectra of SM4 34:0;O4 (*m/z* 812.52, C_40_H_79_NO_13_S) demonstrating why this low-abundance sulfatide remained unannotated by Metaspace despite high-confidence manual identification. Left: spatial distribution map showing localized tissue presence. Center: full mass spectrum window showing the monoisotopic [M-H]⁻ peak clearly visible above noise (1e4 intensity scale). Right: ultra-high-resolution zoom of the isotopic region (1e3 intensity scale) showing that while the monoisotopic peak is detectable, the diagnostic ^13^C₂ and ^34^S isotopic peaks fall entirely below the signal-to-noise threshold and are not detected, rendering isotopic pattern matching impossible. (b) Mass accuracy analysis across the dataset pixels for this species showing mass deviations scatter (left, pixel index), the relationship between intensity and mass accuracy (center, demonstrating the low signal intensity constraint), and a histogram revealing the sparse pixel distribution (right). This N/A example illustrates the fundamental measurement limitation: only the [M-H]⁻ monoisotopic peak is experimentally observable for this low-abundance species, making algorithm-based isotopic envelope matching physically impossible, even with ultra-high mass resolving power.

**Supplementary Table S1**. Overview of sulfatides identified in ARSA^−/−^ kidney by MALDI-MSI. Uncertainties as standard deviation (*n* = 4) in parentheses. Sulfatides with predominant accumulation in the cortex region are marked in italics. Sulfatides uniquely found in Gruber et al. (2025) are marked with an asterisk, and internal standard is marked with a hash mark. SM1a/b sulfatides were not considered here, since their [M-H]^−^ cannot be discriminated from SB1a [M-HSO3]^−^; for details, see Gruber et al. (2025). Detailed feature list for each replicate can be found in Supplementary Dataset 4.

## Abbreviations

ACN: acetonitrile; ARSA: arylsulfatase A; DHAP: 2,5-dihydroxyacetophenone; FDR: false discovery rate; FID: free induction decay; FT-ICR: Fourier transform ion cyclotron resonance; FWHM: full width at half maximum; HPLC: high-performance liquid chromatography; ICR: ion cyclotron resonance; IFS: isotopic fine structure; IMP: inner medulla/papillae; IPRM-PASEF: imaging parallel reaction monitoring–parallel acquisition serial fragmentation; ISOM: inner stripe of outer medulla; LC-MS: liquid chromatography–mass spectrometry; MALDI: matrix-assisted laser desorption/ionization; MIR: mid-infrared; MRMS: magnetic resonance mass spectrometry; MSI: mass spectrometry imaging; *m/z*: mass to charge ratio; QCL: quantum cascade laser; ROI: region of interest; TFA: trifluoroacetic acid; TIMS: trapped ion mobility spectrometry.

## Declaration of Generative AI and AI-Assisted Technologies in the Writing Process

During the preparation of this work, the author(s) used perplexity.ai to improve the readability and language of the manuscript. After using this tool/service, the author(s) reviewed and edited the content as needed and take(s) full responsibility for the content of the published article.

## Supplementary Material

giaf150_Supplemental_Files

giaf150_Authors_Response_To_Reviewer_Comments_Original_Submission

giaf150_GIGA-D-25-00298_original_submission

giaf150_GIGA-D-25-00298_Revision_1

giaf150_Reviewer_1_Report_Original_Submission Morteza Akbari, Ph.D. -- 9/22/2025

giaf150_Reviewer_1_Report_Revision_1 Morteza Akbari, Ph.D. -- 11/9/2025

giaf150_Reviewer_2_Report_Original_Submission Hikmet Budak, PhD -- 10/20/2025

giaf150_Reviewer_2_Report_Revision_1 Hikmet Budak, PhD -- 11/22/2025

## Data Availability

The underlying MALDI MR-MSI raw data supporting the findings of this study are openly available in Zenodo at [44]. The *.imzML* files of the processed MR-MSI are available via Metaspace under [45].

## References

[1] Alexandrov T . Spatial metabolomics and imaging mass spectrometry in the age of artificial intelligence. Annu Rev Biomed Data Sci. 2020;3:61–87. 10.1146/annurev-biodatasci-011420-031537. 34056560 PMC7610844

[2] Baquer G, Sementé L, Mahamdi T, et al. What are we imaging? Software tools and experimental strategies for annotation and identification of small molecules in mass spectrometry imaging. Mass Spectrom Rev. 2023;42:1927–64. 10.1002/mas.21794. 35822576

[3] Cochran D, Powers R. Fourier transform ion cyclotron resonance mass spectrometry applications for metabolomics. Biomedicines. 2024;12:1786. 10.3390/biomedicines12081786. 39200250 PMC11351437

[4] Hess B, Saftig P, Hartmann D et al. Phenotype of arylsulfatase A-deficient mice: relationship to human metachromatic leukodystrophy. Proc Natl Acad Sci USA. 1996;93:14821–26. 10.1073/pnas.93.25.14821. 8962139 PMC26220

[5] Gruber L, Schmidt S, Enzlein T, et al. Deep MALDI-MS spatial omics guided by quantum cascade laser mid-infrared imaging microscopy. Nat Commun. 2025;16:4759. 10.1038/s41467-025-59839-3. 40404613 PMC12098849

[6] Wadie B, Stuart L, Rath C M, et al. METASPACE-ML: context-specific metabolite annotation for imaging mass spectrometry using machine learning. Nat Commun. 2024;15:9110. 10.1038/s41467-024-52213-9. 39438443 PMC11496635

[7] Schulz S, Becker M, Groseclose M R, et al. Advanced MALDI mass spectrometry imaging in pharmaceutical research and drug development. Curr Opin Biotechnol. 2019;55:51–59. 10.1016/j.copbio.2018.08.003. 30153614

[8] Ma X, Fernández F M. Advances in mass spectrometry imaging for spatial cancer metabolomics. Mass Spectrom Rev. 2024;43:235–68. 10.1002/mas.21804. 36065601 PMC9986357

[9] Ngai Y T, Lau D, Mittal P et al. Review: highlight of recent advances and applications of MALDI mass spectrometry imaging in 2024. Anal Sci Adv. 2025;6:e70016. 10.1002/ansa.70016. 40352425 PMC12065102

[10] Abu Sammour D, Cairns J L, Boskamp T, et al. Spatial probabilistic mapping of metabolite ensembles in mass spectrometry imaging. Nat Commun. 2023;14:1823. 10.1038/s41467-023-37394-z. 37005414 PMC10067847

[11] Spangenberg P, Bessler S, Widera L, et al. msiFlow: automated workflows for reproducible and scalable multimodal mass spectrometry imaging and microscopy data analysis. Nat Commun. 2025;16:1065. 10.1038/s41467-024-55306-7. 39870624 PMC11772593

[12] Zhang H, Lu K H, Ebbini M, et al. Mass spectrometry imaging for spatially resolved multi-omics molecular mapping. NPJ Imaging. 2024;2:20. 10.1038/s44303-024-00025-3. 39036554 PMC11254763

[13] Rosenberger F A, Thielert M, Mann M. Making single-cell proteomics biologically relevant. Nat Methods. 2023;20:320–23. 10.1038/s41592-023-01771-9. 36899157

[14] Hu H, Helminiak D, Yang M, et al. High-throughput mass spectrometry imaging with dynamic sparse sampling. ACS Meas Sci Au. 2022;2:466–74. 10.1021/acsmeasuresciau.2c00031. 36281292 PMC9585637

[15] Xie Y R, Castro D C, Rubakhin S S, et al. Enhancing the throughput of FT mass spectrometry imaging using joint compressed sensing and subspace modeling. Anal Chem. 2022;94:5335–43. 10.1021/acs.analchem.1c05279. 35324161 PMC8988892

[16] Cairns J L, Huber J, Lewen A, et al. Mass-guided single-cell MALDI imaging of low-mass metabolites reveals cellular activation markers. Adv Sci (Weinh). 2025;12:e2410506. 10.1002/advs.202410506. 39665230 PMC11791930

[17] Heuckeroth S, Behrens A, Wolf C, et al. On-tissue dataset-dependent MALDI-TIMS-MS2 bioimaging. Nat Commun. 2023; 14:7495. 10.1038/s41467-023-43298-9. PMC10657435 37980348

[18] Patterson N H, Tuck M, van de Plas R, et al. Advanced registration and analysis of MALDI imaging mass spectrometry measurements through autofluorescence microscopy. Anal Chem. 2018;90:12395–403. 10.1021/acs.analchem.8b02884. 30272960

[19] Rabe J-H, A Sammour D, Schulz S, et al. Fourier transform infrared microscopy enables guidance of automated mass spectrometry imaging to predefined tissue morphologies. Sci Rep. 2018;8:313. 10.1038/s41598-017-18477-6. 29321555 PMC5762902

[20] Blutke A, Sun N, Xu Z, et al. Light sheet fluorescence microscopy guided MALDI-imaging mass spectrometry of cleared tissue samples. Sci Rep. 2020;10:14461. 10.1038/s41598-020-71465-1. 32879402 PMC7468256

[21] Choe K, Xue P, Zhao H, et al. macroMS: image-guided analysis of random objects by matrix-assisted laser desorption/ionization time-of-flight mass spectrometry. J Am Soc Mass Spectrom. 2021;32:1180–88. 10.1021/jasms.1c00013. 33822609 PMC8102432

[22] Esselman A B, Patterson N H, Migas L G, et al. Microscopy-directed imaging mass spectrometry for rapid high spatial resolution molecular imaging of glomeruli. J Am Soc Mass Spectrom. 2023;34:1305–14. 10.1021/jasms.3c00033. 37319264

[23] Croslow S W, Trinklein T J, Sweedler J V. Advances in multimodal mass spectrometry for single-cell analysis and imaging enhancement. FEBS Lett. 2024;598:591–601. 10.1002/1873-3468.14798. 38243373 PMC10963143

[24] Gachumi G, Purves R W, Hopf C, et al. Fast quantification without conventional chromatography, the growing power of mass spectrometry. Anal Chem. 2020;92:8628–37. 10.1021/acs.analchem.0c00877. 32510944

[25] Yuan J, Li X, Shen X et al. Comprehensive metabolite profiling in single-cell systems via dual-modal MALDI-mass spectrometry imaging. Anal Chem. 2025;97:8729–37. 10.1021/acs.analchem.4c05480. 40237634

[26] Shi S D, Hendrickson C L, Marshall A G. Counting individual sulfur atoms in a protein by ultrahigh-resolution Fourier transform ion cyclotron resonance mass spectrometry: experimental resolution of isotopic fine structure in proteins. Proc Natl Acad Sci USA. 1998;95:11532–37. 10.1073/pnas.95.20.11532. 9751700 PMC21675

[27] Tiquet M, La Rocca R, Kirnbauer S, et al. FT-ICR mass spectrometry imaging at extreme mass resolving power using a dynamically harmonized ICR cell with 1ω or 2ω detection. Anal Chem. 2022;94:9316–26. 10.1021/acs.analchem.2c00754. 35604839 PMC9260710

[28] Popov I A, Nagornov K, Vladimirov G N, et al. Twelve million resolving power on 4.7 T Fourier transform ion cyclotron resonance instrument with dynamically harmonized cell–observation of fine structure in peptide mass spectra. J Am Soc Mass Spectrom. 2014;25:790–99. 10.1007/s13361-014-0846-7. 24604470

[29] Sun Z, Wang F, Liu Y, et al. Recent strategies for improving MALDI mass spectrometry imaging performance towards low molecular weight compounds. Trends Anal Chem. 2024;175:117727. 10.1016/j.trac.2024.117727.

[30] Schmidt S . M2ira Quant. 2025. https://github.com/CeMOS-Mannheim/QCL_MIR_guided_MSI.Accessed 3 March 2025.

[31] Palmer A, Phapale P, Chernyavsky I, et al. FDR-controlled metabolite annotation for high-resolution imaging mass spectrometry. Nat Methods. 2017;14:57–60. 10.1038/nmeth.4072. 27842059

[32] Roux C, Blaum K, Block M, et al. Data analysis of Q-value measurements for double-electron capture with SHIPTRAP. Eur Phys J D. 2013;67:146. 10.1140/epjd/e2013-40110-x.

[33] Hohenester U M, Barbier Saint-Hilaire P, Fenaille F, et al. Investigation of space charge effects and ion trapping capacity on direct introduction ultra-high-resolution mass spectrometry workflows for metabolomics. J Mass Spectrom. 2020;55:e4613. 10.1002/jms.4613. 32881151

[34] Pieczonka S A, Thomas M J, Schmitt-Kopplin P, et al. Harmonization of FT-ICR-MS instruments for interoperable multi-laboratory comprehensive compositional profiling. Anal Chem. 2025;97:8491–98. 10.1021/acs.analchem.5c00488. 40208963 PMC12019771

[35] Wong R L, Amster I J. Experimental evidence for space-charge effects between ions of the same mass-to-charge in fourier-transform ion cyclotron resonance mass spectrometry. Int J Mass Spectrom. 2007;265:99–105. 10.1016/j.ijms.2007.01.014. 19562102 PMC2701712

[36] Thompson C J, Witt M, Forcisi S, et al. An enhanced isotopic fine structure method for exact mass analysis in discovery metabolomics: FIA-CASI-FTMS. J Am Soc Mass Spectrom. 2020;31:2025–34. 10.1021/jasms.0c00047. 32857936

[37] Alexandrov T . METASPACE annotation platform. 2024. http://www.metaspace2020.eu.Accessed 3 September 2024.

[38] The M, Samaras P, Kuster B, et al. Reanalysis of ProteomicsDB using an accurate, sensitive, and scalable false discovery rate estimation approach for protein groups. Mol Cell Proteomics. 2022;21:100437. 10.1016/j.mcpro.2022.100437. 36328188 PMC9718969

[39] Benigni P, Porter J, Ridgeway M E, et al. Increasing analytical separation and duty cycle with nonlinear analytical mobility scan functions in TIMS-FT-ICR MS. Anal Chem. 2018;90:2446–50. 10.1021/acs.analchem.7b04053. 29376337

[40] Wootton C A, Maillard J, Theisen A, et al. A gated TIMS FTICR MS instrument to decipher isomeric content of complex organic mixtures. Anal Chem. 2024;96:11343–52. 10.1021/acs.analchem.4c01370. 38973712

[41] Vandergrift G W, Zemaitis K J, Veličković D, et al. Experimental assessment of mammalian lipidome complexity using multimodal 21 T FTICR mass spectrometry imaging. Anal Chem. 2023;95:10921–29. 10.1021/acs.analchem.3c00518. 37427698

[42] Grgic A, Nagornov K O, Kozhinov A N, et al. Ultrahigh-mass resolution mass spectrometry imaging with an orbitrap externally coupled to a high-performance data acquisition system. Anal Chem. 2024;96:794–801. 10.1021/acs.analchem.3c04146. 38127459 PMC10794996

[43] Wilkinson M D, Dumontier M, Jan Aalbersberg I, et al. The FAIR Guiding Principles for scientific data management and stewardship. Sci. Data. 2016;3:160018. 10.1038/sdata.2016.18 26978244 PMC4792175

[44] Gruber L . A sulfatide-centered ultra-high resolution magnetic resonance MALDI imaging benchmark dataset for MS1-based lipid annotation tools. Zenodo. 2025. 10.5281/zenodo.16842681.Accessed 13 August 2025. PMC12766628 41363756

[45] Gruber L . imzML files for MR-MSI. 2025. https://metaspace2020.org/api_auth/review?prj=0e7b6e78-78cf-11f0-a049-172853cb2b10&token=EaH8S_2gCKoX. Accessed 14 August 2025.

