# Supplementary material for: A sulfatide-centered ultra-high-resolution magnetic resonance MALDI imaging benchmark dataset for MS1-based lipid annotation tools
Source: Gigascience. 2025 Dec 9;14:giaf150. doi: 10.1093/gigascience/giaf150 (PMC12766628; doi:10.1093/gigascience/giaf150)
Supplement: giaf150_Supplemental_Files [file giaf150_supplemental_files.zip › Gigascience_QCL-MRMS_Suppl_REV final.pdf]

SUPPLEMENTARY DATA

Supplementary Dataset 1. Evaluation of sulfatides peak analysis for conventional 7T XR FT-ICR data with 1s FID time.

Supplementary Dataset 2. Evaluation of sulfatides peak analysis for conventional 7T XR FT-ICR data with 16s FID time.

For data visualization and rapid data interpretation, ion images corresponding to a sulfatide feature list consisting of 126 features were exported from SCILS lab software. Additionally, centroided data derived from mean ion intensities and mass resolving power per peak were exported from Data Analysis software. Each peak was modeled using a Gaussian approximation. Data processing in Python identified the peak position of the monoisotopic mass, $^{13}C_2$, and ^{34}S.

Supplementary Dataset 3. List of theoretical sulfatide configurations provided as .xlsx file.

Supplementary Dataset 4. List of all annotated sulfatide configurations, including mass deviation and IFS indication, for each replicate provided as .xlsx file.

Supplementary Dataset 5. List of all Metaspace sulfatide annotations for both mass resolving powers and each replicate provided as .xlsx file.

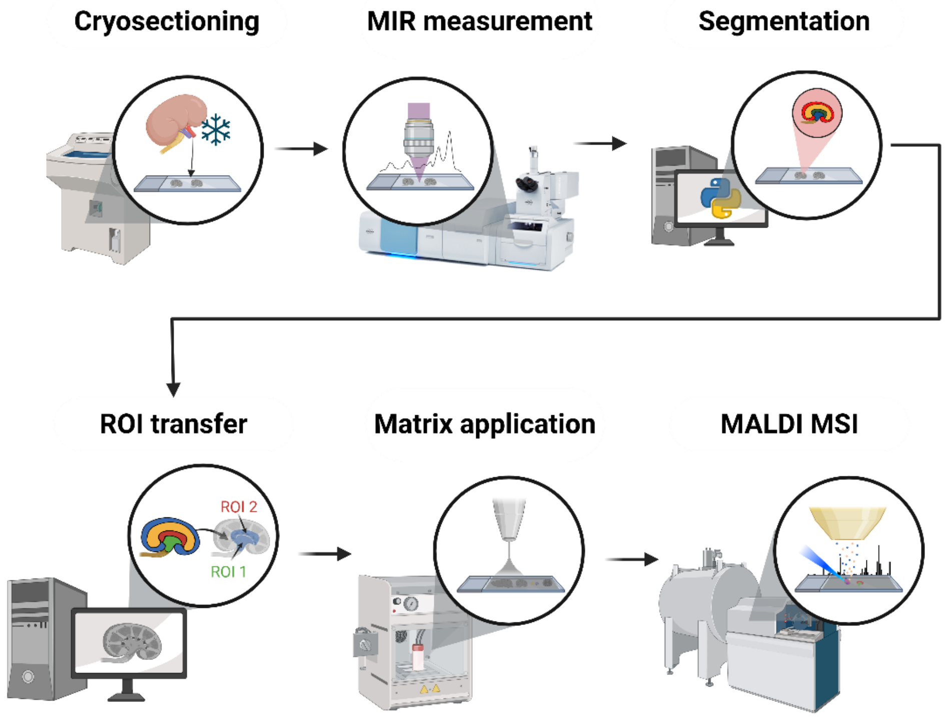

Supplementary Figure 1. Schematic overview of the QCL-MIR imaging guidance and MR-MSI data acquisition workflow.

Tissues (here, kidneys from arylsulfatase A knock-out mice) are cryosectioned and dried and scanned by quantum-cascade laser (QCL) mid-infrared (MIR) imaging microscopy. Hyperspectral datasets are segmented based on chemical composition of the tissue, and regions of interest (ROI) are transferred to an FT-ICR magnetic resonance mass spectrometry imaging (MR-MSI) instrument. The tissue section is spray-coated with a chemical MALDI matrix and subjected to MALDI MS imaging on the MR-MSI system at either high resolution (R ~ 77,000) or ultra-high resolution (R ~ 1,230,000).

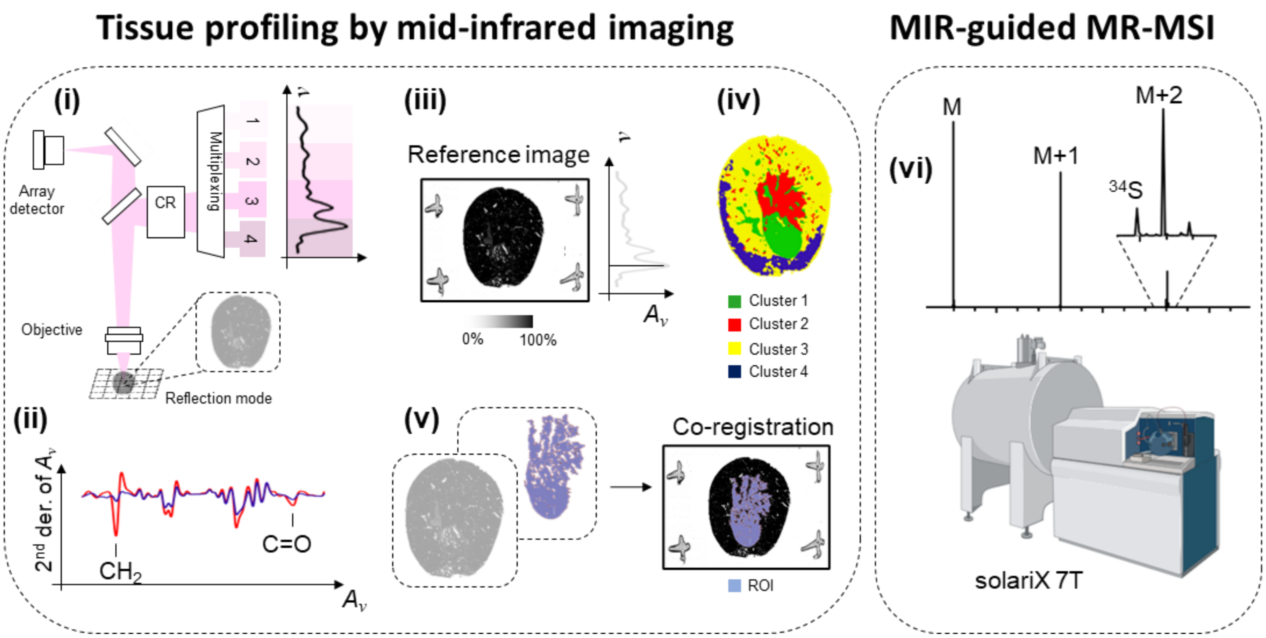

Supplementary Figure 2. Detailed Schematic of the guidance and data acquisition workflow.
Schematic overview of the QCL microscope (i) and the QCL-IRI-guided MSI workflow for fresh-frozen biological specimens in reflection mode. (ii) Key spectral features are selected using the second derivative of absorbance (1466 cm^{-1}, CH_2 bending; 1742 cm^{-1}, C=O stretch). (iii) A single wavenumber (1656 cm^{-1}) image is acquired as a reference and co-registered with the QCL-MIR dataset-derived regions-of-interest (iv and v). (vi) MR-MSI is performed with a focus on these ROIs, enabling the detection of isotopic fine structures of sulfatides. A_ν: absorbance; CR: coherence reduction.

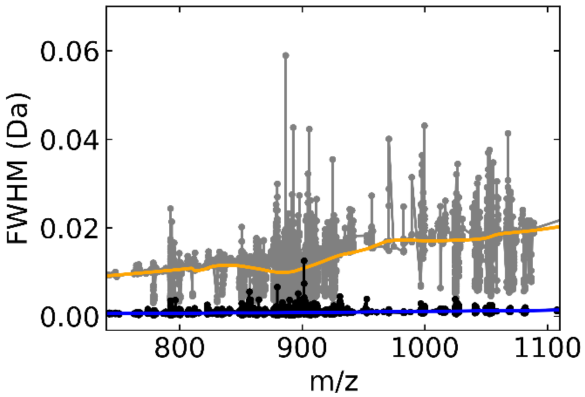

Supplementary Figure 3. Evolution of experimentally deducted FWHM for two different mass resolving powers across the lipid mass range for datasets of a 60-week-old mouse kidney.
Full width at half maximum (FWHM) as a function of the *m/z* value for two different free induction decay (FID) times of 1s (grey, mass resolving power R_1) and 16s (black, mass resolving power R_2). The orange (1s FID) and blue (16s FID) curves result from a locally estimated scatterplot smoothing (loess) and are plotted to guide the eye. On average, the ratio of the FWHM with R_2 to the FWHM with R_1 agrees well with the expected mass resolving power determined by the relative duration of the FID times.

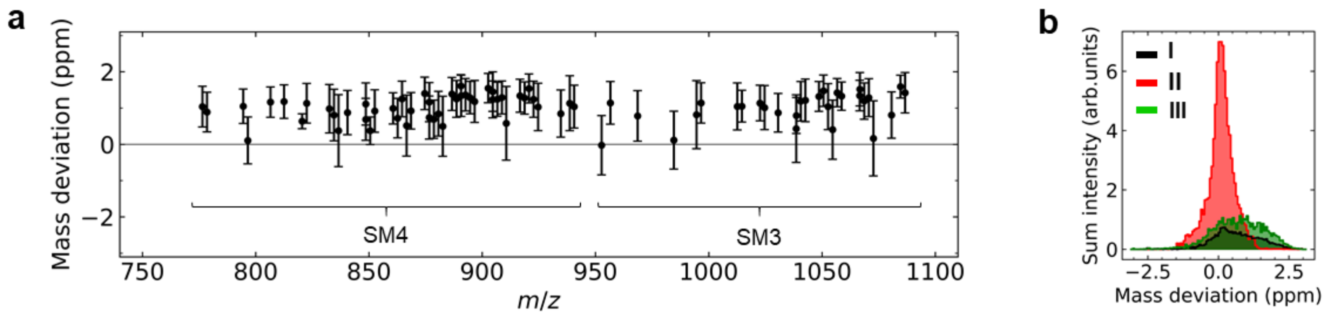

Supplementary Figure 4. Mass deviation across the lipid mass range for R_1~77,000 across n=4 biological replicates of ARSA-/- mouse kidney.
a, Mean mass deviation for sulfatides identified by MR-MSI (solariX 7T XR) with a mass resolution of R_1~77,000 at *m/z* 800. Means of mass deviation and standard deviations for each sulfatide were obtained from the sum intensity histograms in b, which resulted from the mean *m/z* value (determined across all pixels) of a given sulfatide. **b**, Histogram of the sum intensity for the three sulfatides I SM4 34:1;O2[M-H]$^-$, II SM4 38:1;O3[M-H]$^-$, and III SM3 42:1;O2[M-H]$^-$.

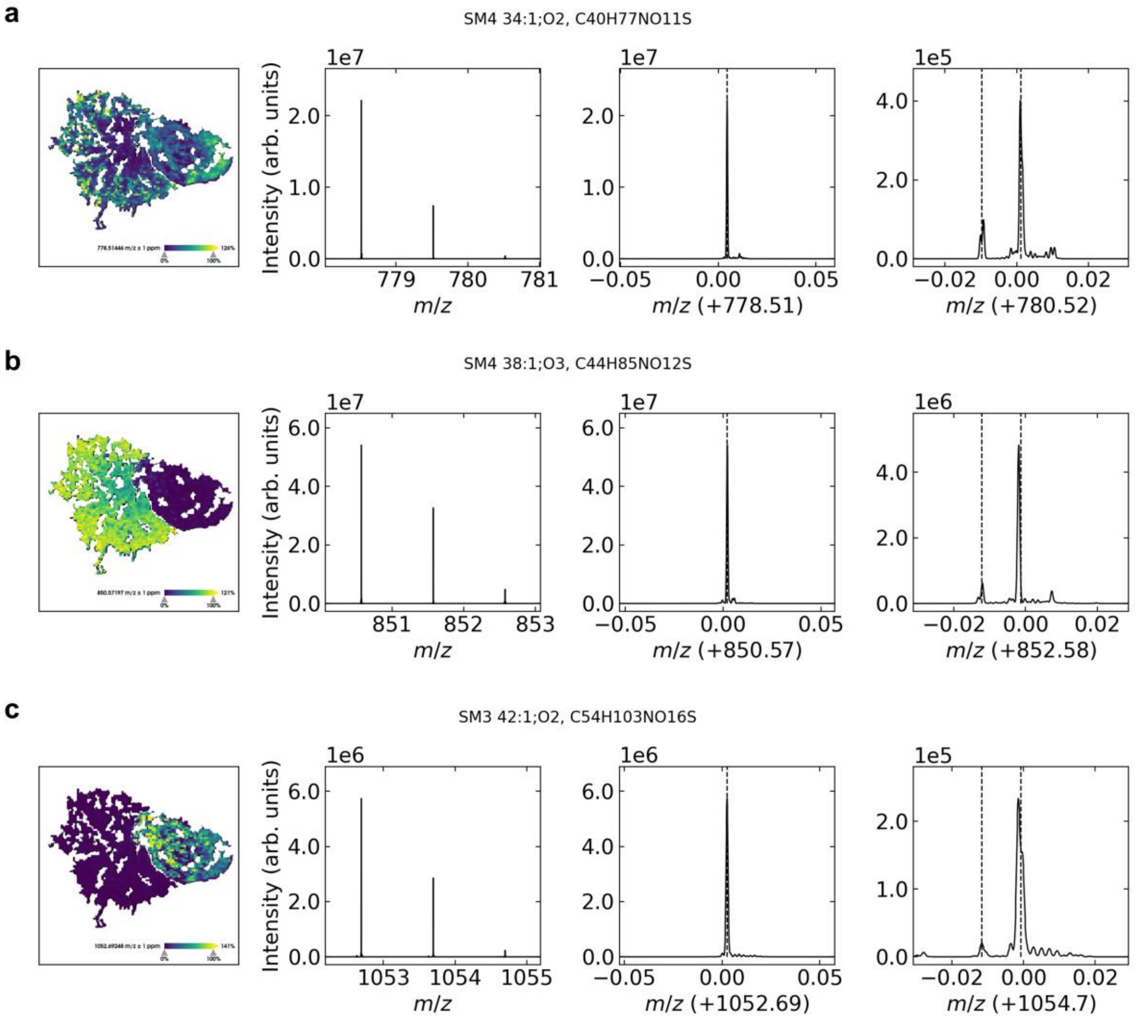

Supplementary Figure 5. Spatial distribution of three sulfatide species and their respective isotopic fine structure.
a, Ultra-high resolution MRMS data for I *m/z* 778.5146 (SM4 34:1;O2[M-H]$^-$; **b**, for II *m/z* 850.5721 (SM4 38:1;O3[M-H]$^-$; **c**, and III *m/z* 1052.6923 (SM3 42:1;O2[M-H] $^-$, acquired with a mass resolution of R_2~1,230,000 using QCL-MIR imaging-guided

MR-MSI. Positions of the isotopic fine structure (IFS) peak, including $^{13}C_2$ (M+2) and ^{34}S, are highlighted by dotted lines, respectively. Respective data for all other sulfatides are presented in the Supplementary Dataset 2. Pixel size, 40 µm.

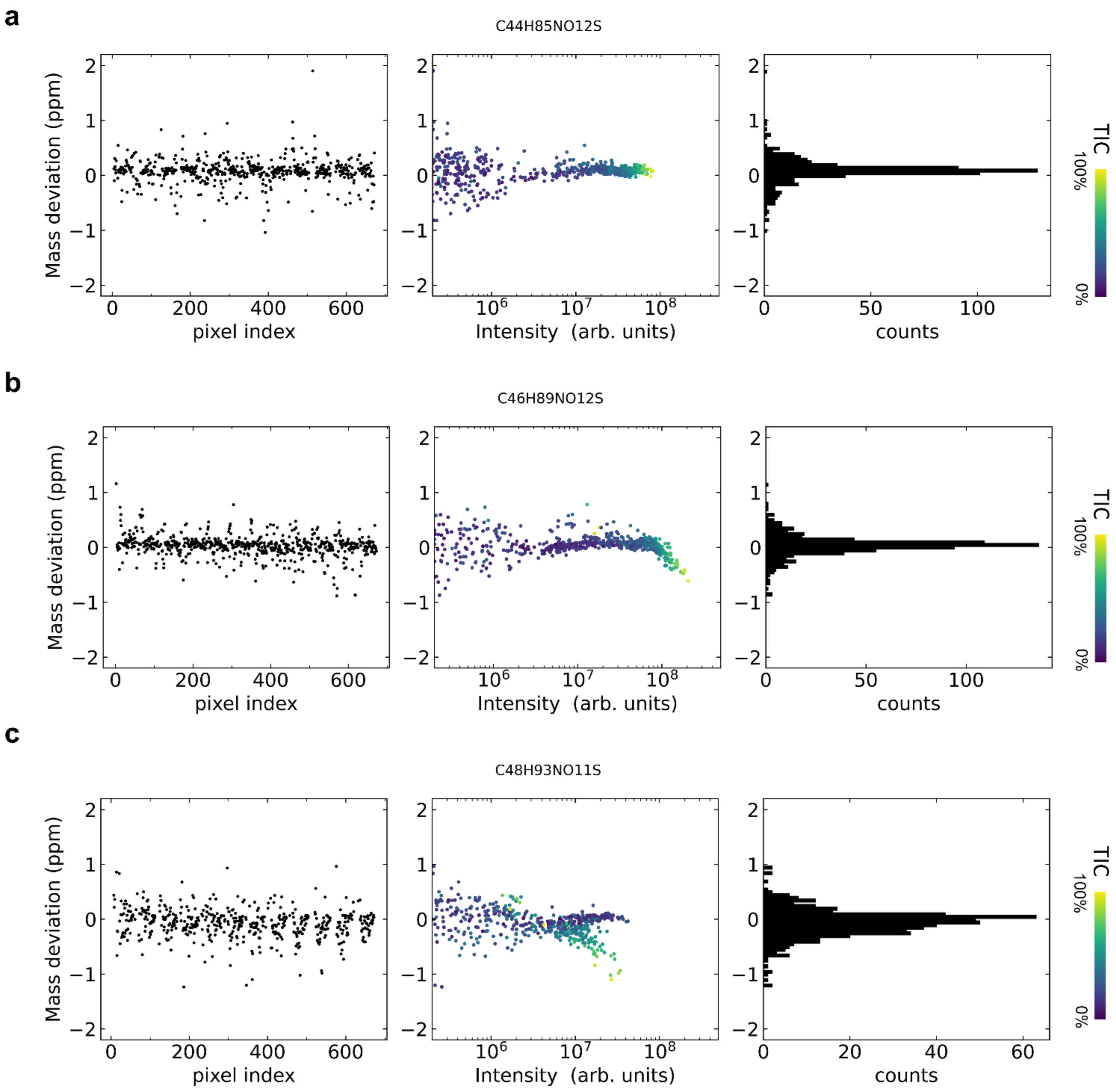

Supplementary Figure 6. Observation of space-charge effects for three sulfatide examples.
a, Evaluation for *m/z* 850.5720 (SM4 38:1;O3$[M-H]^-$; **b**, Evaluation for *m/z* 878.6033 (SM4 40:1;O3$[M-H]^-$; and **c**, Evaluation for *m/z* 890.6397 (SM4 42:1;O2$[M-H]^-$. All data was acquired with a mass resolution of R_2~1,230,000 using QCL-MIR imaging-guided MR-MSI. Bin width for histogram, 0.05 ppm.

a

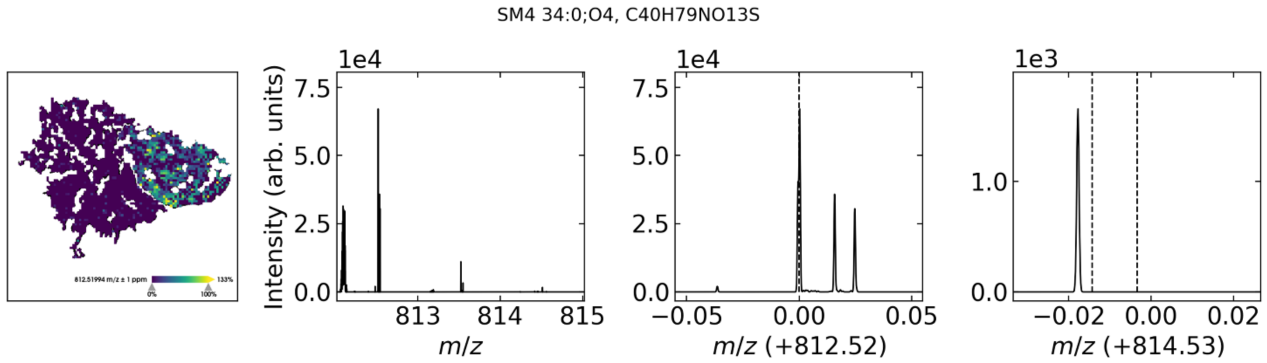

b

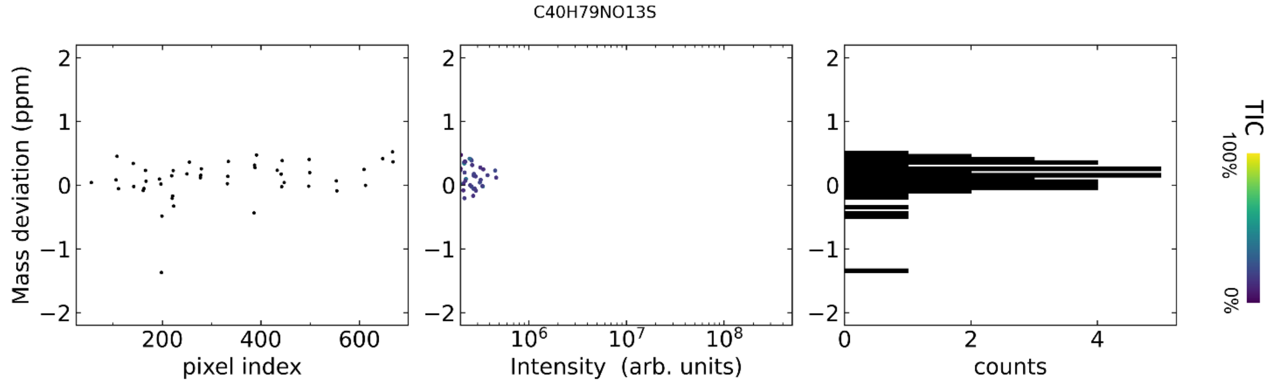

Supplementary Figure 7. Exemplar of a missing annotation (N/A case): SM4 34:0;O4 with undetectable isotopic fine structure. a, Spatial distribution and mass spectra of SM4 34:0;O4 (m/z 812.52, $C_{40}H_{79}NO_{13}S$) demonstrating why this low-abundance sulfatide remained unannotated by Metaspace despite high-confidence manual identification. Left: spatial distribution map showing localized tissue presence. Center: full mass spectrum window showing the monoisotopic $[M-H]^-$ peak clearly visible above noise (1e4 intensity scale). Right: ultra-high-resolution zoom of the isotopic region (1e3 intensity scale) showing that while the monoisotopic peak is detectable, the diagnostic $^{13}C_2$ and ^{34}S isotopic peaks fall entirely below the signal-to-noise threshold and are not detected, rendering isotopic pattern matching impossible. b, Mass accuracy analysis across the dataset pixels for this species showing mass deviations scatter (left, pixel index), the relationship between intensity and mass accuracy (center, demonstrating the low signal intensity constraint), and a histogram revealing the sparse pixel distribution (right). This N/A example illustrates the fundamental measurement limitation: only the $[M-H]^-$ monoisotopic peak is experimentally observable for this low-abundance species, making algorithm-based isotopic envelope matching physically impossible, even with ultra-high mass resolving power.

Supplementary Table 1. Overview of sulfatides identified in ARSA-/- kidney by MALDI-MSI.
Uncertainties as standard deviation (n=4) in parentheses. Sulfatides with predominant accumulation in the cortex region are marked in italics. Sulfatides uniquely found in (Gruber *et al.* 2025) are marked with an asterisk and internal standard is marked with a hash mark. SM1a/b sulfatides were not considered here, since their $[M-H]^-$ can't be discriminated from SB1a $[M-HSO3]^-$, for details see (Gruber *et al.* 2025). Detailed feature list for each replicate can be found as Supplementary Dataset 4.

Name	Sum formula	m/z theo.	Adduct	*m/z* FTICR	ppm FTICR
SM4 18:1;O2	C24H47NO10S	540.284791	$[M-H]^-$	540.28479	-0.097
*SM4 18:0;O3	C24H49NO11S	558.295356	$[M-H]^-$		
*SM4 32:2;O2	C38H71NO11S	748.467507	$[M-H]^-$		
*SM4 32:1;O2	C38H73NO11S	750.483157	$[M-H]^-$		
*SM4 32:2;O3	C38H71NO12S	764.462422	$[M-H]^-$		

*SM4 32:1;O3	C38H73NO12S	766.478072	[M-H]⁻		
SM4 34:2;O2	C40H75NO11S	776.498807	[M-H]⁻	776.49881	0.061
*SM4 33:2;O3	C39H73NO12S	778.478072	[M-H]⁻		
SM4 34:1;O2	C40H77NO11S	778.514457	[M-H]⁻	778.51446	0.116
*SM4 34:2;O3	C40H75NO12S	792.493722	[M-H]⁻		
#SM4 35:1;O2	C41H79NO11S	792.530107	[M-H]⁻		
SM4 34:1;O3	C40H77NO12S	794.509372	[M-H]⁻	794.50937	0.006
SM4 34:0;O3	C40H79NO12S	796.525022	[M-H]⁻	796.52502	0.008
SM4 36:2;O2	C42H79NO11S	804.530107	[M-H]⁻	804.53011	-0.079
SM4 36:1;O2	C42H81NO11S	806.545757	[M-H]⁻	806.54576	0.124
SM4 34:0;O4	C40H79NO13S	812.519936	[M-H]⁻	812.51994	0.123
SM4 36:2;O3	C42H79NO12S	820.525022	[M-H]⁻	820.52502	0.077
*SM4 37:1;O2	C43H83NO11S	820.561407	[M-H]⁻		
SM4 36:1;O3	C42H81NO12S	822.540672	[M-H]⁻	822.54067	-0.056
*SM4 36:0;O3	C42H83NO12S	824.556322	[M-H]⁻		
SM4 38:2;O2	C44H83NO11S	832.561407	[M-H]⁻	832.56141	0.084
SM4 38:1;O2	C44H85NO11S	834.577057	[M-H]⁻	834.57706	0.051
SM4 37:1;O3	C43H83NO12S	836.556322	[M-H]⁻	836.55632	0.006
SM4 36:0;O4	C42H83NO13S	840.551237	[M-H]⁻	840.55124	-0.04
*SM4 39:2;O2	C45H85NO11S	846.577057	[M-H]⁻		
SM4 38:2;O3	C44H83NO12S	848.556322	[M-H]⁻	848.55632	0.075
SM4 39:1;O2	C45H87NO11S	848.592707	[M-H]⁻	848.59271	0.066
SM4 38:1;O3	C44H85NO12S	850.571972	[M-H]⁻	850.57197	0.154
*SM4 38:0;O3	C44H87NO12S	852.587622	[M-H]⁻		
SM4 40:2;O2	C46H87NO11S	860.592707	[M-H]⁻	860.59271	0.118
SM4 40:1;O2	C46H89NO11S	862.608357	[M-H]⁻	862.60836	0.057
SM4 39:1;O3	C45H87NO12S	864.587622	[M-H]⁻	864.58762	0.041
*SM4 39:0;O3	C45H89NO12S	866.603272	[M-H]⁻		
SM4 38:0;O4	C44H87NO13S	868.582536	[M-H]⁻	868.58254	0.132
SM4 41:2;O2	C47H89NO11S	874.608357	[M-H]⁻	874.60836	0.134
SM4 40:2;O3	C46H87NO12S	876.587622	[M-H]⁻	876.58762	0.162
SM4 41:1;O2	C47H91NO11S	876.624007	[M-H]⁻	876.62401	0.063
SM4 40:1;O3	C46H89NO12S	878.603272	[M-H]⁻	878.60327	-0.077
*SM4 40:0;O3	C46H91NO12S	880.618922	[M-H]⁻		
*SM4 39:0;O4	C45H89NO13S	882.598187	[M-H]⁻		
SM4 42:3;O2	C48H89NO11S	886.608357	[M-H]⁻	886.60836	0.013
SM4 42:2;O2	C48H91NO11S	888.624007	[M-H]⁻	888.62401	-0.122
SM4 41:2;O3	C47H89NO12S	890.603272	[M-H]⁻	890.60327	-0.035
SM4 42:1;O2	C48H93NO11S	890.639657	[M-H]⁻	890.63966	-0.103
SM4 41:1;O3	C47H91NO12S	892.618922	[M-H]⁻	892.61892	-0.235
*SM4 41:0;O3	C47H93NO12S	894.634572	[M-H]⁻		
SM4 40:0;O4	C46H91NO13S	896.613837	[M-H]⁻	896.61384	-0.119
SM4 42:3;O3	C48H89NO12S	902.603272	[M-H]⁻	902.60327	0.083
SM4 42:2;O3	C48H91NO12S	904.618922	[M-H]⁻	904.61892	0.149
SM4 43:1;O2	C49H95NO11S	904.655307	[M-H]⁻	904.65531	0.032
SM4 42:1;O3	C48H93NO12S	906.634572	[M-H]⁻	906.63457	-0.073
*SM4 42:0;O3	C48H95NO12S	908.650222	[M-H]⁻		
*SM4 41:0;O4	C47H93NO13S	910.629487	[M-H]⁻		
*SM4 44:3;O2	C50H93NO11S	914.639657	[M-H]⁻		
SM4 44:2;O2	C50H95NO11S	916.655307	[M-H]⁻	916.65531	-0.033
SM4 44:1;O2	C50H97NO11S	918.670957	[M-H]⁻	918.67096	0.087
SM4 43:1;O3	C49H95NO12S	920.650222	[M-H]⁻	920.65022	-0.034
SM4 42:1;O4	C48H93NO13S	922.629487	[M-H]⁻	922.62949	0.007
SM4 42:0;O4	C48H95NO13S	924.645137	[M-H]⁻	924.64514	-0.046
*SM4 44:2;O3	C50H95NO12S	932.650222	[M-H]⁻		
SM4 44:1;O3	C50H97NO12S	934.665872	[M-H]⁻	934.66587	-0.085
*SM4 43:0;O4	C49H97NO13S	938.660787	[M-H]⁻		
SM4 46:1;O2	C52H101NO11S	946.702258	[M-H]⁻	946.70226	0.323
*SM4 44:0;O4	C50H99NO13S	952.676438	[M-H]⁻		
*SM3 18:1;O2	C30H57N1O15S1	702.337615	[M-H]⁻		
*SM3 18:0;O3	C30H59N1O16S1	720.348180	[M-H]⁻		
SM3 34:1;O2	C46H87N1O16S1	940.567281	[M-H]⁻	940.56728	0.100
SM3 34:1;O3	C46H87N1O17S1	956.562196	[M-H]⁻	956.56220	0.113
SM3 36:1;O2	C48H91N1O16S1	968.598581	[M-H]⁻	968.59858	-0.054
*SM3 36:1;O3	C48H91N1O17S1	984.593496	[M-H]⁻		
*SM3 38:2;O2	C50H93N1O16S1	994.614231	[M-H]⁻		
SM3 38:1;O2	C50H95N1O16S1	996.629881	[M-H]⁻	996.62988	0.053
*SM3 38:2;O3	C50H93N1O17S1	1010.609146	[M-H]⁻		
*SM3 39:1;O2	C51H97N1O16S1	1010.645531	[M-H]⁻		
SM3 38:1;O3	C50H95N1O17S1	1012.624796	[M-H]⁻	1012.62480	0.082

SM3 38:0;O3	C50H97N1O17S1	1014.640446	[M-H]$^-$	1014.64044	-0.226
SM3 40:2;O2	C52H97N1O16S1	1022.645531	[M-H]$^-$	1022.64553	0.121
SM3 40:1;O2	C52H99N1O16S1	1024.661181	[M-H]$^-$	1024.66118	0.068
*SM3 38:0;O4	C50H97N1O18S1	1030.635360	[M-H]$^-$		
SM3 40:2;O3	C52H97N1O17S1	1038.640446	[M-H]$^-$	1038.64044	-0.207
SM3 41:1;O2	C53H101N1O16S1	1038.676831	[M-H]$^-$	1038.67683	-0.099
SM3 40:1;O3	C52H99N1O17S1	1040.656096	[M-H]$^-$	1040.65610	0.144
SM3 40:0;O3	C52H101N1O17S1	1042.671746	[M-H]$^-$	*1042.67175*	0.036
SM3 42:3;O2	C54H99N1O16S1	1048.661181	[M-H]$^-$	1048.66118	-0.196
SM3 42:2;O2	C54H101N1O16S1	1050.676831	[M-H]$^-$	1050.67683	0.163
SM3 42:1;O2	C54H103N1O16S1	1052.692481	[M-H]$^-$	1052.69248	-0.075
SM3 41:1;O3	C53H101N1O17S1	1054.671746	[M-H]$^-$	1054.67175	0.101
*SM3 41:0;O3	C53H103N1O17S1	1056.687396	[M-H]$^-$		
SM3 40:0;O4	C52H101N1O18S1	1058.666661	[M-H]$^-$	1058.66666	0.029
*SM3 42:3;O3	C54H99N1O17S1	1064.656096	[M-H]$^-$		
SM3 42:2;O3	C54H101N1O17S1	1066.671746	[M-H]$^-$	1066.67175	0.103
SM3 43:1;O2	C55H105N1O16S1	1066.708131	[M-H]$^-$	1066.70813	0.071
SM3 42:1;O3	C54H103N1O17S1	1068.687396	[M-H]$^-$	1068.68740	0.146
SM3 42:0;O3	C54H105N1O17S1	1070.703046	[M-H]$^-$	*1070.70305*	0.073
*SM3 41:0;O4	C53H103N1O18S1	1072.682311	[M-H]$^-$		
*SM3 44:2;O2	C56H105N1O16S1	1078.708131	[M-H]$^-$		
SM3 44:1;O2	C56H107N1O16S1	1080.723781	[M-H]$^-$	1080.72378	0.111
SM3 42:1;O4	C54H103N1O18S1	1084.682311	[M-H]$^-$	1084.68231	0.743
SM3 42:0;O4	C54H105N1O18S1	1086.697961	[M-H]$^-$	1086.69796	0.101
SM3 44:1;O3	C56H107N1O17S1	1096.718696	[M-H]$^-$	1096.71870	0.009
*SM3 46:1;O2	C58H111N1O16S1	1108.755082	[M-H]$^-$		
*SM2a 38:1;O2	C58H108N2O21S1	1199.709253	[M-H]$^-$		
*SM2a 38:1;O3	C58H108N2O22S1	1215.704168	[M-H]$^-$		
*SM2a 40:1;O2	C60H112N2O21S1	1227.740553	[M-H]$^-$		
*SM2a 40:1;O3	C60H112N2O22S1	1243.735468	[M-H]$^-$		
*SM2a 42:1;O2	C62H116N2O21S1	1255.771853	[M-H]$^-$		
*SM2a 42:1;O3	C62H116N2O22S1	1271.766768	[M-H]$^-$		
SB1a 34:1;O2	C60H110N2O29S2	1305.699477	[M-HSO$_3$]$^-$	1305.69948	0.167
SB1a 34:1;O3	C60H110N2O30S2	1321.694392	[M-HSO$_3$]$^-$	1321.69439	-0.131
SB1a 36:1;O2	C62H114N2O29S2	1333.730777	[M-HSO$_3$]$^-$	1333.73078	0.379
SB1a 36:1;O3	C62H114N2O30S2	1349.725692	[M-HSO$_3$]$^-$	1349.72569	0.341
SB1a 38:1;O2	C64H118N2O29S2	1361.762077	[M-HSO$_3$]$^-$	1361.76208	0.019
SB1a 39:1;O2	C65H120N2O29S2	1375.777727	[M-HSO$_3$]$^-$	1375.77773	0.419
*SB1a 38:1;O3	C64H118N2O29S2	1377.756991	[M-HSO$_3$]$^-$		
SB1a 40:2;O2	C66H120N2O29S2	1387.777727	[M-HSO$_3$]$^-$	1387.77773	0.505
SB1a 40:1;O2	C66H122N2O29S2	1389.793377	[M-HSO$_3$]$^-$	1389.79338	0.170
*SB1a 38:0;O4	C64H120N2O31S2	1395.767556	[M-HSO$_3$]$^-$		
SB1a 41:1;O2	C67H124N2O29S2	1403.809027	[M-HSO$_3$]$^-$	1403.80903	0.103
SB1a 40:1;O3	C66H122N2O30S2	1405.788292	[M-HSO$_3$]$^-$	1405.78829	-0.023
SB1a 42:2;O2	C68H124N2O29S2	1415.809027	[M-HSO$_3$]$^-$	1415.80903	-0.053
SB1a 42:1;O2	C68H126N2O29S2	1417.824677	[M-HSO$_3$]$^-$	1417.82468	0.077
*SB1a 40:0;O4	C66H124N2O31S2	1423.798857	[M-HSO$_3$]$^-$		
SB1a 42:2;O3	C68H124N2O30S2	1431.803942	[M-HSO$_3$]$^-$	1431.80394	-0.120
SB1a 42:1;O3	C68H126N2O30S2	1433.819592	[M-HSO$_3$]$^-$	1433.81959	0.087
*SB1a 41:0;O4	C67H126N2O31S2	1437.814507	[M-HSO$_3$]$^-$		
*SB1a 44:1;O2	C70H130N2O29S2	1445.855977	[M-HSO$_3$]$^-$		
*SB1a 42:0;O4	C68H128N2O31S2	1451.830157	[M-HSO$_3$]$^-$		
