# Supplementary material for: A sulfatide-centered ultra-high-resolution magnetic resonance MALDI imaging benchmark dataset for MS1-based lipid annotation tools
Source: Gigascience. 2025 Dec 9;14:giaf150. doi: 10.1093/gigascience/giaf150 (PMC12766628; doi:10.1093/gigascience/giaf150)
Supplement: giaf150_GIGA-D-25-00298_original_submission [file giaf150_giga-d-25-00298_original_submission.pdf]

GigaScience

A sulfatide-centered ultra-high resolution magnetic resonance MALDI imaging benchmark dataset for MS1-based lipid annotation tools

--Manuscript Draft--

**Manuscript Number:**	GIGA-D-25-00298
**Full Title:**	A sulfatide-centered ultra-high resolution magnetic resonance MALDI imaging benchmark dataset for MS1-based lipid annotation tools
**Article Type:**	Data Note
**Funding Information:**	Bundesministerium für Bildung und Forschung (12FH8I05IA) — Prof. Carsten Hopf<br>Ministerium für Wissenschaft, Forschung und Kunst Baden-Württemberg (Mittelbauprogramm) — Prof. Carsten Hopf<br>Deutsche Forschungsgemeinschaft (262133997) — Prof. Carsten Hopf
**Abstract:**	Spatial 'omics techniques are indispensable for studying complex biological systems and for the discovery of spatial biomarkers. While several current matrix-assisted laser desorption/ionization (MALDI) mass spectrometry imaging (MSI) instruments are capable of localizing numerous metabolites at high spatial and spectral resolution, the majority of MSI data is acquired at the MS1 level only. Assigning molecular identities based on MS1 data presents significant analytical and computational challenges, as the inherent limitations of MS1 data preclude confident annotations beyond the sum formula level. To enable future advancements of computational lipid annotation tools, well-characterized benchmark - or ground truth - datasets are crucial, which exceed the scope of synthetic data or data derived from mimetic tissue models. To this end, we provide two sulfatide-centered, biology-driven magnetic resonance MSI (MR-MSI) datasets at different mass resolving powers that characterize lipids in a mouse model of human metachromatic dystrophy. This data includes an ultra-high-resolution (R ~1,230,000) quantum cascade laser mid-infrared imaging-guided MR-MSI dataset that enables isotopic fine structure analysis and therefore enhances the level of confidence substantially. To highlight the usefulness of the data, we compared 118 manual sulfatide annotations with the number of decoy database-controlled sulfatide annotations performed in Metaspace (67 at FDR < 10%). Overall, our datasets can be used to benchmark annotation algorithms, validate spatial biomarker discovery pipelines, and serve as a reference for future studies that explore sulfatide metabolism and its spatial regulation.
**Corresponding Author:**	Carsten Hopf, Ph.D.<br>HS Mannheim: Hochschule Mannheim<br>MANNHEIM, GERMANY
**Corresponding Author Secondary Information:**	
**Corresponding Author's Institution:**	HS Mannheim: Hochschule Mannheim
**Corresponding Author's Secondary Institution:**	
**First Author:**	Lars Gruber
**First Author Secondary Information:**	
**Order of Authors:**	Lars Gruber<br>Stefan Schmidt<br>Thomas Enzlein<br>Carsten Hopf
**Order of Authors Secondary Information:**	

Powered by Editorial Manager® and ProduXion Manager® from Aries Systems Corporation

Additional Information:	
**Question**	**Response**
Are you submitting this manuscript to a special series or article collection?	No
**Experimental design and statistics**<br><br>Full details of the experimental design and statistical methods used should be given in the Methods section, as detailed in our Minimum Standards Reporting Checklist. Information essential to interpreting the data presented should be made available in the figure legends.<br><br>Have you included all the information requested in your manuscript?	Yes
**Resources**<br><br>A description of all resources used, including antibodies, cell lines, animals and software tools, with enough information to allow them to be uniquely identified, should be included in the Methods section. Authors are strongly encouraged to cite Research Resource Identifiers (RRIDs) for antibodies, model organisms and tools, where possible.<br><br>Have you included the information requested as detailed in our Minimum Standards Reporting Checklist?	Yes
**Availability of data and materials**<br><br>All datasets and code on which the conclusions of the paper rely must be either included in your submission or deposited in publicly available repositories (where available and ethically appropriate), referencing such data using a unique identifier in the references and in the “Availability of Data and Materials” section of your manuscript.	Yes

Have you have met the above requirement as detailed in our Minimum Standards Reporting Checklist?	
GigaScience has policies and guidelines in place for the use of generative AI-writing tools such as ChatGPT. If you have used such writing tools to assist with writing the manuscript this must be declared and cited in the text. Authors should not list AI-writing tools and other AI-assisted technologies as an author or co-author and should acknowledge that they are fully responsible for text generated or refined by AI-writing tools.<p><br><br>A summary of use (particularly in the introduction or among methods) needs to be included at the end of the paper, and the outputs should also be included as a supplementary file hosted in GigaDB or other open repositories. Please read our guidelines for more information. <p><br><br>By submitting to GigaScience, you are aware of the journal's AI-writing tools policy, and if you have declared use of such tools below, you have acknowledged this where appropriate in your manuscript and have made a summary of use and outputs available. <p><br><b>AI-assisted writing tools have been used in the preparation of this manuscript?	Yes

Manuscript

Click here to access/download;Manuscript;Gigascience_QCL-MRMS_Main_clean_new.docx

A sulfatide-centered ultra-high resolution magnetic resonance MALDI imaging benchmark dataset for MS1-based lipid annotation tools

Lars Gruber[1,2], Stefan Schmidt[1], Thomas Enzlein[1], Carsten Hopf[1,2,3,*]

[1]Center for Mass Spectrometry and Optical Spectroscopy (CeMOS), Technische Hochschule Mannheim, Paul-Wittsack-Str. 10, 68165 Mannheim

[2]Medical Faculty, Heidelberg University, Im Neuenheimer Feld 280, 69117 Heidelberg, Germany

[3]Mannheim Center for Translational Neuroscience (MCTN), Medical Faculty Mannheim, Heidelberg University, Theodor Kutzer-Ufer 1-3, 68167 Mannheim, Germany

*To whom correspondence should be addressed.

ABSTRACT: Spatial 'omics techniques are indispensable for studying complex biological systems and for the discovery of spatial biomarkers. While several current matrix-assisted laser desorption/ionization (MALDI) mass spectrometry imaging (MSI) instruments are capable of localizing numerous metabolites at high spatial and spectral resolution, the majority of MSI data is acquired at the MS1 level only. Assigning molecular identities based on MS1 data presents significant analytical and computational challenges, as the inherent limitations of MS1 data preclude confident annotations beyond the sum formula level. To enable future advancements of computational lipid annotation tools, well-characterized benchmark - or ground truth - datasets are crucial, which exceed the scope of synthetic data or data derived from mimetic tissue models. To this end, we provide two sulfatide-centered, biology-driven magnetic resonance MSI (MR-MSI) datasets at different mass resolving powers that characterize lipids in a mouse model of human metachromatic dystrophy. This data includes an ultra-high-resolution (R ~1,230,000) quantum cascade laser mid-infrared imaging-guided MR-MSI dataset that enables isotopic fine structure analysis and therefore enhances the level of confidence substantially. To highlight the usefulness of the data, we compared 118 manual sulfatide annotations with the number of decoy database-controlled sulfatide annotations performed in Metaspace (67 at FDR < 10%). Overall, our datasets can be used to benchmark annotation algorithms, validate spatial biomarker discovery pipelines, and serve as a reference for future studies that explore sulfatide metabolism and its spatial regulation.

Keywords: MALDI mass spectrometry, MRMS, MALDI imaging, mass spectrometry imaging, metabolite annotation tools, mid-infrared imaging, isotope fine structure, lipidomics, metabolomics

DATA DESCRIPTION

The absence of ground truth datasets, i.e., prior knowledge of which metabolites/lipid are present (or not) in a tissue of interest, and the non-availability of corresponding datasets containing high-confidence annotations for a large number of metabolites/lipids, has been posing a major obstacle to computational advancements in mass spectrometry imaging (MSI) [1]. In particular, datasets that can challenge computational tools for molecular annotation will be crucial for rapid progress in the field [2]. To this end, we generated reusable and widely applicable datasets comprising quadruplicates of spatially focused, high-resolution mass spectrometry imaging data (MS1 level) derived from kidneys of an arylsulfatase A-deficient (ARSA-/-) mouse, a well-known genetic model of human metachromatic leukodystrophy [3]. Specifically, we developed a workflow that leverages Quantum Cascade Laser Mid-infrared (QCL-MIR) imaging to guide MSI on a 7T FT-ICR magnetic resonance mass spectrometer (MR-MS; **Supplementary Fig. 1 and 2**). The resulting MS1 data was interpreted in conjunction with precise reference annotations obtained for sulfatide glycosphingolipid species in defined kidney regions by on-tissue fragmentation-based lipid identification using imaging parallel reaction monitoring - parallel acquisition serial fragmentation (iprm-PASEF) on an orthogonal trapped ion mobility spectrometry (tims) TOF mass spectrometer [4]. Through combination of ultra-high resolution (R ~ 1,230,000) MR-MSI MS1 data with systematic MS2 data obtained on a different mass spectrometer, we are establishing a concept for generating such benchmark datasets. Four biological replicates and cross-modal validation against 4D-lipidomics TIMS-MS ensure data quality [4]. The ultra-high resolution dataset was further intended to be complemented by a high-resolution dataset. All files and preprocessing scripts are publicly available, thus supporting benchmarking and integration within spatial omics analyses, as further demonstrated in this work using Metaspace-ML [5].

CONTEXT

MALDI mass spectrometry imaging (MSI) has evolved into an invaluable tool in spatial biology [6, 7] that enables the label-free detection and statistically validated visualization of molecular distributions in tissues [8, 9]. However, achieving reliable bimolecular interpretation of the inherently complex spatial molecular patterns fundamentally depends on the availability of high-quality datasets featuring unambiguous molecular identifications. Such datasets are crucial for facilitating the discovery of spatial biomarkers and yielding insights into tissue function, pathology, and pharmacodynamic or therapeutic responses [10, 11].

Prompted by the instrumental limitations outlined, for instance, in the 4S paradigm [7], several specialized methodologies for subspace imaging have been developed to facilitate the generation of high-quality mass spectrometry imaging (MSI) datasets, including spatial sparse sampling strategies [12, 13] or guided approaches. The latter comprises mass-guided approaches, e.g., single-cell imaging [14] or on-tissue MS2 [15] and imaging-guided approaches [16–20], including the recently developed QCL-MIR imaging-guided MSI workflow [4]. In general, these workflows have been introduced with the objective of enhancing overall throughput. Sometimes, acquisition time saved by restricting MSI to defined ROIs is reallocated to alternative workflows that operate MSI with advanced instrumental settings for MS1 data. These alternative workflows can include adjustments to laser beam settings [21], increased transient durations in FT-ICR MSI, or optimized ramp times in TIMS-MSI, which improve spatial resolution, mass resolution, and ion mobility separation, respectively. Furthermore, imaging-based guidance methods can be combined with a sophisticated MSI technique for on-tissue MS2 that utilizes ion mobility-enhanced methods such as iprm-PASEF [4, 15]. This integration substantially increased data quality by enhancing the confidence level for molecular identifications, all without the need for high-performance liquid chromatography (HPLC) separation and directly in the spatial context of the tissue [22]. Notably, the exploration of the chemical space of sulfatide isoforms in an ARSA-/- mouse model enabled us to introduce a ground truth to the MSI field, since sulfatides are known to accumulate in distinct ROIs of kidney sections from these mice.

As our QCL-MIR imaging-based guidance approach is inherently instrument-agnostic, we have applied it in this study to create an ultra-high-resolution, sulfatide-focused MS1 benchmark dataset using a 7T XR FT-ICR (**Supplementary Fig. 1 and 2**). Using externally validated annotations, this dataset may become a unique benchmark resource (**Fig. 1a**) for developing and enhancing MS1 tools for deep spatial lipidomics and related research fields. This is especially important, because most MSI studies today still rely on acquiring only MS1 data [23, 24].

By default, annotations of MS1 data are limited to the sum formula level. For this and other reasons, the unambiguous annotation of sum formulae to MS1 data remains a non-trivial task, even with ultra-high mass resolving power (R > 500.000) spectra, due to the sheer chemical diversity within biological samples [2, 25, 26]. Consequently, a single precise mass measurement may correspond to multiple candidate sum formulae, thereby complicating definitive assignment, even when the isotopic fine structures (IFS) can be resolved. Nevertheless, the progressive exploitation of accurate mass, isotopic envelope, and IFS information [27–32] substantially increases the reliability of metabolite annotation in MR-MSI-based spatial ‘omics studies, a process in which computational tools play a crucial role by enabling automated annotation workflows. For future advancement of such tools in MSI, well-characterized benchmark datasets with high mass accuracy will be essential to enable robust validation and method development. This highlights the necessity and reuse potential of the dataset introduced in this study.

METHODS

Quantum-cascade laser mid-infrared (QCL-MIR) imaging of mouse kidneys

Animal studies involving ARSA-/- mice and cryo-sectioning of kidneys have been described before [4]. To ultimately focus ultra-high resolution MR-MSI data generation on defined kidney regions of interest (ROI) on an adjacent tissue section, we used QCL-MIR imaging for a pre-scan, followed by segmentation of hyperspectral QCL-MIR data to define ROIs [4]. These were then transferred to the MR-MSI instrument (**Supplementary Fig. 1 and 2**). To this end, QCL-MIR imaging data was recorded in sweep scan mode within a spectral range of 950–1800 cm^{-1} at a spectral sampling interval of 4 cm^{-1} on a Hyperion II ILIM (Bruker Optics, Ettlingen, Germany) equipped with a 3.5x objective. Subsequently, ROIs were generated and selected using *in-house* software (https://github.com/CeMOS-Mannheim/QCL_MIR_guided_MSI) based on spatial sulfatide distributions in ARSA-/- mouse kidneys, which predominantly occur in the Inner Medulla/Papillae (IMP) and Inner Stripe of Outer Medulla (ISOM). Specifically, these ROIs were then targeted for MR-MSI data acquisition with transient times of 15.7s. All measurements were repeated for n=4 biological replicates.

Matrix spray-coating

10 mg/mL DHAP was dissolved in 70% ACN with 125 mM ammonium sulfate. After sonication, 0.1% TFA and 3 µM of SM4 35:1;O2 (100 µg/mL (= 157.41 µM) in MeOH/chloroform 2:1) as internal standard (IS) were added. Matrix was applied with an M5 TM-Sprayer (HTX Technologies, Chapel Hill, USA). Temperatures of the spray nozzle and tray were 75 °C and 35 °C, respectively. The spraying parameters were as follows: Spray Nozzle Velocity: 1200 mm/min; Flow Rate: 0.1 mL/min; No. of Passes: 10; Track Spacing: 2 mm; Pattern: HH; Pressure: 10 psi; Gas Low Rate: 2 L/min; Nozzle Height: 40 mm; Drying Time: 0s.

Magnetic resonance mass spectrometry imaging (MR-MSI) data acquisition

Ultra-high resolution MSI data was acquired on a solariX 7T XR Fourier Transform Ion Cyclotron Resonance (FT-ICR) MS (Bruker Daltonics, Bremen, Germany), equipped with a smartbeam II 2 kHz laser and ftms control 2.3.0 software (Bruker Daltonics, Build 92). Mass spectra were acquired in negative ion mode (*m/z* range 401.29–2600) and data acquisition size of 8 M, resulting in a free induction decay (FID) time of 15.7s according to and a mass resolving power of 1,230,000 at *m/z* 800. Reducing the number of data points in the time domain to 512k resulted in an FID time of 0.98 s and a mass resolving power of R~77,000. Ion optics settings were constant for all measurements: funnel RF amplitude (150 Vpp), source octopole (5 MHz, 350 Vpp), and collision cell voltage: 1.5 V, cell: 2 MHz, 1200 Vpp. The source DC optics were also constant for all measurements (capillary exit: -200 V, deflector plate: -220 V, funnel 1: -150 V, skimmer 1: -15 V), as well as the ParaCell parameters (transfer exit lens: 30 V, analyzer entrance: 10 V, sidekick: 0 V, side kick offset: 1.5 V, front/back trap plate: -3.4 V, back trap plate quench: 30 V). Sweet excitation power for ion detection was set to 14 %, and ion accumulation time was 0.05 s. The transfer optics were as follows: time of flight: 1 ms, frequency: 4 MHz, and RF amplitude: 350 Vpp. The laser parameters were laser power: 32 %, laser shots: 20, laser frequency: 200 Hz, and laser focus: medium, at a lateral step size of 40 µm.

Mass spectrometry imaging data analysis

For state-of-the-art annotation with Metaspace [33], the v2 (Metaspace ML [5]) algorithm and an *m/z* tolerance of 2 ppm were utilized. The *imzML* files were exported from SCiLS Lab (Version 2024a Pro, Bruker Daltonics). For manual annotation, we compared the QCL-MIR-guided MR-MSI data against the ground truth data from [4]. For direct comparison of ultra-high- and high-resolution datasets, MS-based data segmentation was employed to enable subspace modeling. Bisecting k-means clustering of the MSI data from whole-tissue kidney sections was performed via SCiLS Lab to delineate anatomical regions, specifically the inner stripe of the outer medulla (ISOM) and the Inner Medulla/Papillae (IMP). These region outlines were subsequently used to generate a region-focused dataset, facilitating a more targeted comparative analysis.

MATERIALS

All chemicals and solvents were of HPLC-MS grade. Conductive indium tin oxide (ITO)-coated glass slides were purchased from Diamond Coatings (West Midlands, UK). The MALDI matrix 2,5-dihydroxyacetophenone (DHAP) was purchased from Thermo Fisher Scientific (Waltham, Massachusetts, USA). Acetonitrile (ACN), ethanol (EtOH), LC-MS water, 2-propanol (IPA), and ammonium sulfate (AmS) were obtained from VWR Chemicals (Darmstadt, Germany). The sulfatide standard C_{17} mono-sulfo galactosyl(β) ceramide d18:1/17:0 (SM4 35:1;O2) was purchased from Avanti Polar Lipids (Birmingham, USA). Trifluoroacetic acid (TFA), Mayer's hemalum solution, hydrochloric acid, sodium bicarbonate, magnesium sulfate, eosin Y-solution 0.5%, xylene, and eukitt were purchased from Merck KGaA.

DATA VALIDATION AND QUALITY CONTROL

To enable the advancement of MS^1-based molecular annotation tools for spatial biomarker discovery pipelines and other purposes, we acquired and in-depth-characterized two sulfatide-centered, biology-driven datasets derived from an arylsulfatase A mouse model at two different mass resolving powers. This includes an ultra-high mass resolution MR-MSI dataset (R ~ 1,230,000) acquired using our recently introduced Quantum Cascade Laser (QCL) MIR guidance approach [4], in this study, combined with a 7T FT-ICR mass spectrometer. Briefly summarized, this workflow utilizes QCL-MIR imaging microscopy to rapidly acquire hyperspectral data from tissue samples, here, fresh-frozen kidney sections. Subsequent application of unsupervised segmentation algorithms, specifically k-means clustering, allows for the identification of biologically relevant regions of interest (ROIs) within the kidney tissue, particularly inner medulla/papilla (IMP) and inner stripe of outer medulla (ISOM), where sulfatides are enriched in ARSA -/- mice. Ultra-high-resolution MR-MSI was then performed in a ROI-targeted manner on these two tissue morphologies within kidneys from two 12-week-old and two 60-week-old ARSA-/- mice (**Fig. 1a; Supplementary Fig. 1** and **2**). The QCL-MIR imaging enabled focus on just two morphologies, enhancing both analytical depth and data acquisition efficiency, e.g., measurement time. As a reference and benchmark for the molecular content of these tissue areas and the total number of potential sulfatide identifications, we relied on our published reference data, which is based on three pillars: a known biological pathway leading to lipid-class specific accumulation of sulfatides, iprm-PASEF-derived molecular identifications in conjunction with 4D lipidomics LC-MS data [4] (**Supplementary Table 1**).

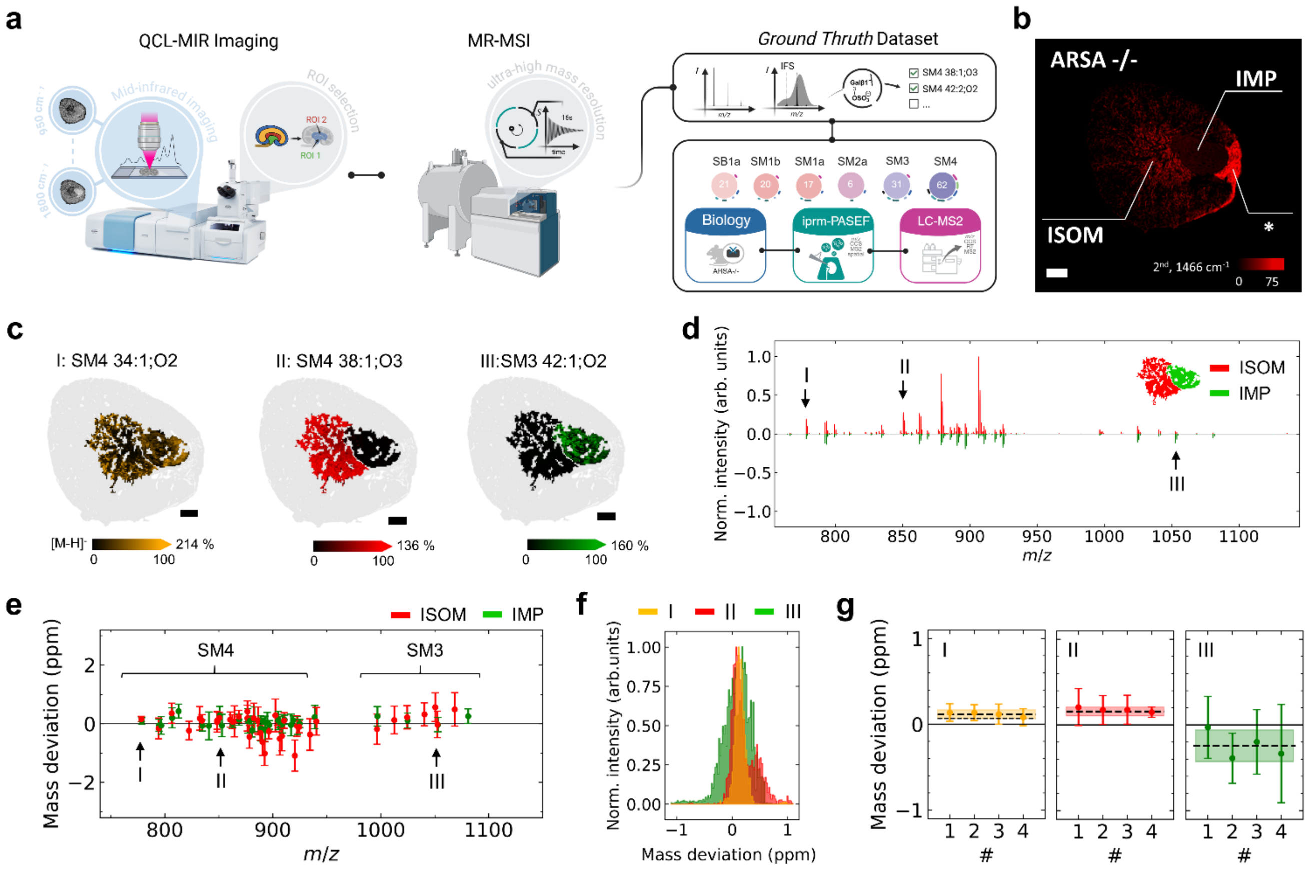

Figure 1. A sulfatide-centered, ultra-high resolution QCL-MIR guided MSI benchmark dataset.
a, Schematic overview of data acquisition and incorporation of reference information. The methodology includes quantum cascade laser mid-infrared (QCL-MIR) imaging of kidney sections for region-focused magnetic resonance mass spectrometry imaging (MR-MSI) at ultra-long transient times (~ 16s), followed by manual sulfatide annotations based on a reference dataset validated at MS2 level [4]. **b,** Representative lipid-distribution in an ARSA-/- kidney section based on the 2nd derivative of absorbance (2nd) at 1466 cm^{-1} yielded predominant sulfatide accumulation in the ISOM region. The asterisk marks a region of high lipid content as described in [4] . Scale bar, 500 µm. **c,** Overlay of region-focused ion images of **(I)** *m/z* 778.5146 (SM4 34:1;O2[M-H]$^-$; orange), **(II)** *m/z* 850.5721 (SM4 38:1;O3[M-H]$^-$; red), and **(III)** *m/z* 1052.6923 (SM3 42:1;O2[M-H]$^-$; green) in kidney (grey). Mass window, ±3 ppm. **d,** Representative butterfly plot of average mass spectra for the inner medulla/papilla (IMP; green) and ISOM (red) identified by QCL-MIR. **e,** Mass deviation and uncertainties (standard deviation) for 47 sulfatides (signals present in at least 50 pixels of either region IMP or ISOM) at R_2~1.23 M. *m/z* values are shifted by +0.2 (IMP) or -0.2 (ISOM) for visualization. **f,** Histogram of sum intensities in ISOM and IMP for (I), (II), and (III) measured with a mass resolution of ~1.23M at *m/z* 800. **g,** Weighted mean mass deviation (dotted line) and uncertainty (*n*=4) presented as internal error[1] (filled area) for (I), (II), and (III).

We compared conventional MR-MSI of whole kidney slices at a mass resolution of R_1~77,000 at $_{m/z}$ 800 (1s free induction decay (FID) time; 14,331 pixels, 40 x 40 µm^2 pixel size; 5 hours of data acquisition) in the FT-ICR with QCL-MIR-guided analysis (**Fig. 1b**) focused on the ISOM and IMP ROIs, which achieved R_2~1,230,000 at *m/z* 800 (16s FID time; 2,672 pixels, 40 x 40 µm^2 pixel size; 11.6 hours of data acquisition), resulting in a 16-fold increase in mass resolving power (**Supplementary Fig. 3**). For the QCL-MIR-guided MR-MSI dataset, three sulfatide ion images are presented as examples that displayed similar intensities in both ROIs (**I**, *m/z* 778.5146 (SM4 34:1;O2[M-H]$^-$)), higher intensity in ISOM (**II**, *m/z* 850.5721 (SM4 38:1;O3[M-H]$^-$)), or higher intensity in IMP (**III**, m/z 1052.6923 [SM3 42:1;O2[M-H]-)). Unique molecular fingerprints were obtained per region (**Fig. 1d**). In both datasets, the mass deviation was constant across the *m/z* range (750-1100) and was consistently below 2 ppm, even when considering the uncertainties across n=4 biological replicates (**Fig. 2e; Supplementary Fig. 4**). The maximum mass deviation was about 1 ppm for the two less intense sulfatide ions **(I)** and **(III)** and less than 0.2 ppm for ion **(II)** at R_1~77,000, improving to below 0.2 ppm for **(I)** and **(II)** (around 0.5 ppm for **(III)**) with a mass resolution of R_2~1,230,000 (**Fig. 1f; Supplementary Fig. 4**). The reproducibility of our data is emphasized by comparing the mass deviation across n=4 biological replicates for ions **(I)-(III)**, all of which showed values below 1 ppm (**Fig. 1g**). Overall, the uncertainty of *m/z* values was reduced by a factor of 3-6 for data acquired at ultra-high resolution on an FT-ICR instrument.

The ultra-high mass resolving power applied to the QCL-MIR dataset enables the detection of isotopic fine structures (IFS) for the sulfatides. The IFS, particularly the peak attributed to ^{34}S, was very well resolved, with a signal-to-noise ratio of approximately 10 times the FWHM (**Fig. 2a; Supplementary Fig. 5; Supplementary Dataset 2**). Nevertheless, it is important to note that in all cases where FID times are notably prolonged, it is necessary to operate with a reduced total ion current. This precautionary measure is pivotal to reduce (local) space charge effects [34–37] within the ion cyclotron resonance (ICR) cell (**Supplementary Fig. 6**). However, this results in a loss of sensitivity, which in turn leads to slightly reduced numbers of sulfatide annotations, in particular for those isoforms that have a comparatively lower concentration in IMP and ISOM than in the cortex. Overall, the number of candidate sulfatide identifications using the QCL-MIR imaging-guided ultra-high-mass resolution MR-MSI method was 91 and 97 for the two 60-week ARSA-/- mice, compared to 118 and 115 annotations obtained with the conventional whole-tissue method (**Table 1**). The identification confidence was, on the other hand, dramatically improved, as 34 and 39 ultra-high-mass resolution spectra were supported by IFS information (**Table 1**). Identification was performed manually, as IFS is currently not utilized adequately in commercial sum formula annotation tools, which match experimental against theoretical isotope patterns [29]. In the sulfatide case, the ^{34}S isotope peak was not automatically recognized as such, but pre-definition of S and N as constituents of the molecule-of-interest in the Bruker Smart Formula tool led to successful searches.

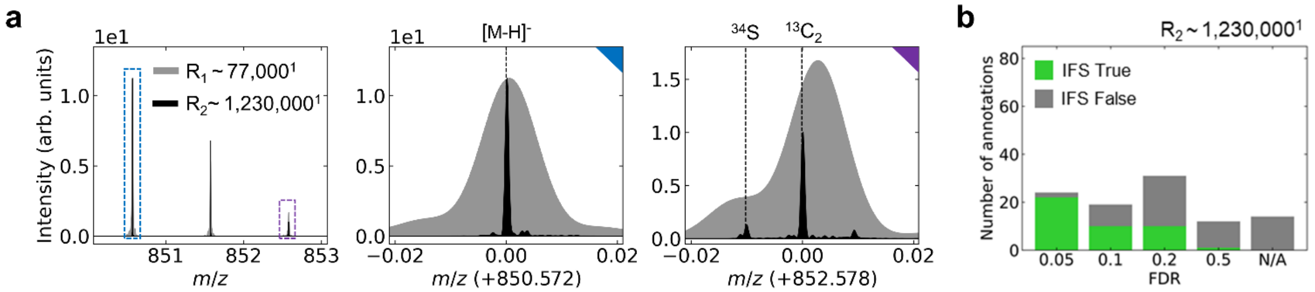

Figure 2. Evaluation of the annotation quality for the sulfatide-centered QCL-MIR guided MSI dataset.
a, Ultra-high resolution MR-MSI data was acquired with a mass resolution of R_1~77,000 (gray) and R_2~1,230,000 (black). Isotopic fine structure (IFS) of SM4 38:1;O3[M-H]$^-$ incl. $^{13}C_2$ (M+2) and ^{34}S isotopic peaks, normalized to the monoisotopic peak. **b,** Sulfatides were annotated using Metaspace, utilizing an in-house database consisting of LipidMaps fed with 780 theoretical sulfatides. [1]Mass resolving power at *m/z* 800. N/A marks annotations that were performed manually (and validated via *in-situ* MS/MS and/or LC-MS/MS) but were not annotated by Metaspace at any FDR level.

To highlight the potential reuse of our dataset as a benchmark for cutting-edge MALDI MSI data annotation, we used the open-source Metaspace platform (http://www.metaspace2020.eu). We compared the number of decoy database-controlled sulfatide annotations at different false-discovery rates (FDR). FDR-based quality measures have recently been critically assessed in the more mature field of proteomics [38], as i) FDR determination is often a black box, and ii) it is often unclear at what level FDR is set. For MSI, the FDR-control process in Metaspace is fairly transparent [33]. Since available databases do not yet sufficiently cover sulfatides, we manually created a database consisting of LipidMaps, which was additionally supplemented with 780 theoretical sulfatide structures (**Supplementary Dataset 3**). For data acquired at 77,000 mass resolving power, IMP and ISOM were extracted using MS feature-based segmentation to maintain consistency in the analysis. At the lowest FDR of 5%, we identified 24 sulfatide annotations for the 1.23M mass resolving power data, of which 22 were supported by IFS (**Fig. 2b**). In contrast, only 11 sulfatides were annotated in the 77k mass resolving power data (**Supplementary Fig. 7**). At 10% FDR, the ultra-high-resolution data yielded 43 annotations (32 supported by IFS), while the 77k mass resolving power data yielded 29 annotations. The number of unlikely annotations with FDR ≥20% was similar, with 74 (with 42 IFS; 1230k) and 77 (77k) sulfatide annotations, respectively. However, compared to our manual annotation, 14 (with 1 IFS; 1230k) and 19 (77k) sulfatides remained unannotated, even with an FDR of ≥50%. It should be noted that within the Metaspace annotation algorithm, only the four most intense peaks are recognized, regardless of whether an IFS is available. Overall, it is clear that even with high data quality, the current community standard tool, Metaspace, cannot eliminate the need for manual inspection of datasets.

RE-USE POTENTIAL

This rigorously validated (see [4]) sulfatide-focused benchmark dataset offers substantial re-use potential for the MSI community. Acquired at two levels of mass resolving power, the dataset enables a comprehensive evaluation of annotation strategies, particularly those that leverage isotopic fine structure (IFS) for high-confidence MS1-level metabolite identification. As demonstrated, IFS analysis provides greater annotation accuracy than current automated platforms, underscoring the need for further methodological innovation in computational annotation. Although studies describing ultra-high resolution exist [39, 40], none of these studies shared their data under FAIR principles, and no externally validated annotations exist. This dataset serves as a valuable resource for benchmarking and validating spatial biomarker discovery

pipelines, developing new annotation algorithms, and supporting future studies into sulfatide metabolism and its spatial regulation in biological tissues. Its broad applicability ensures relevance for both computational tool development and biological research, facilitating advances in spatial metabolomics and related fields.

DATA AVAILABILITY

The underlying MALDI MR-MSI raw data supporting the findings of this study are openly available in Zenodo at http://doi.org/10.5281/zenodo.16842680. The *.imzML* files of the processed MR-MSI are available via Metaspace under https://metaspace2020.org/api_auth/review?prj=0e7b6e78-78cf-11f0-a049-172853cb2b10&token=EaH8S_2gCKoX.

CODE AVAILABILITY

Data acquisition was conducted using existing tools (e,g, https://github.com/CeMOS-Mannheim/QCL_MIR_guided_MSI) and methods as described in the Methods section.

DECLARATIONS

List of abbreviations

ARSA	Arylsulfatase A
FDR	False discovery rate
FID	Free induction decay
FT-ICR	Fourier transform ion cyclotron resonance
FWHM	Full width at half maximum
IFS	Isotopic fine structure
IMP	Inner medulla/papillae
ISOM	Inner stripe of outer medulla
LC-MS	Liquid chromatography mass spectrometry
MALDI	Matrix-assisted laser desorption/ionization
MIR	Mid-infrared
MRMS	Magnetic resonance mass spectrometry
MSI	Mass spectrometry imaging
m/z	mass to charge ratio
QCL	Quantum cascade laser
ROI	Region of interest

Author Contributions (CRediT)

Lars Gruber: Methodology, Investigation, Formal Analysis, Writing - Original Draft
Stefan Schmidt: Methodology, Investigation, Formal Analysis, Writing - Original Draft
Thomas Enzlein: Formal Analysis, Visualization
Carsten Hopf: Conceptualization, Supervision, Project Administration, Resources, Writing - Review & Editing

Funding (FundRef)

This work was supported by:
- The Bundesministerium für Bildung und Forschung (BMBF) under grants 12FH8I05IA (Drugs4Future) and 13FH8I09IA (DrugsData) within the M2Aind partnership (to Carsten Hopf);
- The Ministerium für Wissenschaft, Forschung und Kunst Baden-Württemberg (MWK) via the Mittelbauprogramm (to Carsten Hopf);
- The Deutsche Forschungsgemeinschaft (DFG, project 262133997) for the acquisition of the solariX 7T XR (to Carsten Hopf).

The funders had no role in study design, data collection/analysis, interpretation, or manuscript preparation.

Competing Interests

Bruker Daltonics co-funded the BMBF-funded projects "Drugs4Future" and "DrugsData" within the framework M^{2}Aind, as mandated by BMBF, but did not influence this study. All other authors declare no competing interests.

DECLARATION OF GENERATIVE AI AND AI-ASSISTED TECHNOLOGIES IN THE WRITING PROCESS

During the preparation of this work, the author(s) used perplexity.ai to improve the readability and language of the manuscript. After using this tool/service, the author(s) reviewed and edited the content as needed and take(s) full responsibility for the content of the published article.

ASSOCIATED CONTENT

Supporting Information

The Supporting Information is available free of charge.

AUTHOR INFORMATION

Corresponding Author

*Carsten Hopf, c.hopf@hs-mannheim.de, Center for Mass Spectrometry and Optical Spectroscopy (CeMOS), Technische Hochschule Mannheim, Paul-Wittsack-Str. 10, 68165 Mannheim

Table 1: Cumulative numbers of sulfatide subclass isoforms identified in ARSA-/- mouse kidney by QCL-MIR imaging-guided MR-MSI.

Whole kidney sections of 12- or 60-week-old ARSA-/- mice (n=2 each) were analyzed by conventional non-guided MR-MSI with a mass resolution of R_1 ~ 77k (at m/z 800) and QCL-MIR imaging-guided MR-MSI with a mass resolution of R_2 ~ 1,230k (at *m/z* 800). In many cases of QCL-MIR imaging-guided MSI, isotope fine structures (IFS) could be used for added confidence.

			SM4	SM3	SM2a	SB1a	total
R_1, FID 1s	all	60w_1	56	37	6	19	118
		60w_2	54	36	6	19	115
		12w_1	45	36	6	16	103
		12w_2	52	36	6	18	112
R_2, FID 16s QCL-MIR-guided	all	60w_1	54	25	0	12	91
		60w_2	54	27	4	12	97
		12w_1	42	16	1	8	67
		12w_2	52	19	1	5	77
	Using IFS	60w_1	25	7	0	2	34
		60w_2	27	8	0	4	39
		12w_1	17	4	0	1	22
		12w_2	20	6	0	0	26

References

1. Alexandrov T. Spatial Metabolomics and Imaging Mass Spectrometry in the Age of Artificial Intelligence. Annu Rev Biomed Data Sci. 2020;3:61–87. doi:10.1146/annurev-biodatasci-011420-031537.

2. Baquer G, Sementé L, Mahamdi T, Correig X, Ràfols P, García-Altares M. What are we imaging? Software tools and experimental strategies for annotation and identification of small molecules in mass spectrometry imaging. Mass Spectrom Rev. 2023;42:1927–64. doi:10.1002/mas.21794.

3. Hess B, Saftig P, Hartmann D, Coenen R, Lüllmann-Rauch R, Goebel HH, et al. Phenotype of arylsulfatase A-deficient mice: relationship to human metachromatic leukodystrophy. Proc Natl Acad Sci U S A. 1996;93:14821–6. doi:10.1073/pnas.93.25.14821.

4. Gruber L, Schmidt S, Enzlein T, Vo HG, Bausbacher T, Cairns JL, et al. Deep MALDI-MS spatial omics guided by quantum cascade laser mid-infrared imaging microscopy. Nat Commun. 2025;16:4759. doi:10.1038/s41467-025-59839-3.

5. Wadie B, Stuart L, Rath CM, Drotleff B, Mamedov S, Alexandrov T. METASPACE-ML: Context-specific metabolite annotation for imaging mass spectrometry using machine learning. Nat Commun. 2024;15:9110. doi:10.1038/s41467-024-52213-9.

6. Ma X, Fernández FM. Advances in mass spectrometry imaging for spatial cancer metabolomics. Mass Spectrom Rev. 2024;43:235–68. doi:10.1002/mas.21804.

7. Schulz S, Becker M, Groseclose MR, Schadt S, Hopf C. Advanced MALDI mass spectrometry imaging in pharmaceutical research and drug development. Curr Opin Biotechnol. 2019;55:51–9. doi:10.1016/j.copbio.2018.08.003.

8. Abu Sammour D, Cairns JL, Boskamp T, Marsching C, Kessler T, Ramallo Guevara C, et al. Spatial probabilistic mapping of metabolite ensembles in mass spectrometry imaging. Nat Commun. 2023;14:1823. doi:10.1038/s41467-023-37394-z.

9. Spangenberg P, Bessler S, Widera L, Bottek J, Richter M, Thiebes S, et al. msiFlow: automated workflows for reproducible and scalable multimodal mass spectrometry imaging and microscopy data analysis. Nat Commun. 2025;16:1065. doi:10.1038/s41467-024-55306-7.

10. Rosenberger FA, Thielert M, Mann M. Making single-cell proteomics biologically relevant. Nat Methods. 2023;20:320–3. doi:10.1038/s41592-023-01771-9.

11. Zhang H, Lu KH, Ebbini M, Huang P, Lu H, Li L. Mass spectrometry imaging for spatially resolved multi-omics molecular mapping. Npj Imaging. 2024;2:20. doi:10.1038/s44303-024-00025-3.

12. Xie YR, Castro DC, Rubakhin SS, Sweedler JV, Lam F. Enhancing the Throughput of FT Mass Spectrometry Imaging Using Joint Compressed Sensing and Subspace Modeling. Anal Chem. 2022;94:5335–43. doi:10.1021/acs.analchem.1c05279.

13. Hu H, Helminiak D, Yang M, Unsihuay D, Hilger RT, Ye DH, Laskin J. High-Throughput Mass Spectrometry Imaging with Dynamic Sparse Sampling. ACS Meas Sci Au. 2022;2:466–74. doi:10.1021/acsmeasuresciau.2c00031.

14. Cairns JL, Huber J, Lewen A, Jung J, Maurer SJ, Bausbacher T, et al. Mass-Guided Single-Cell MALDI Imaging of Low-Mass Metabolites Reveals Cellular Activation Markers. Adv Sci (Weinh). 2025;12:e2410506. doi:10.1002/advs.202410506.

15. Heuckeroth S, Behrens A, Wolf C, Fütterer A, Nordhorn ID, Kronenberg K, et al. On-tissue dataset-dependent MALDI-TIMS-MS2 bioimaging. Nat Commun. 2023;14:7495. doi:10.1038/s41467-023-43298-9.

16. Esselman AB, Patterson NH, Migas LG, Dufresne M, Djambazova KV, Colley ME, et al. Microscopy-Directed Imaging Mass Spectrometry for Rapid High Spatial Resolution Molecular Imaging of Glomeruli. J Am Soc Mass Spectrom. 2023;34:1305–14. doi:10.1021/jasms.3c00033.

17. Choe K, Xue P, Zhao H, Sweedler JV. macroMS: Image-Guided Analysis of Random Objects by Matrix-Assisted Laser Desorption/Ionization Time-of-Flight Mass Spectrometry. J Am Soc Mass Spectrom. 2021;32:1180–8. doi:10.1021/jasms.1c00013.

18. Blutke A, Sun N, Xu Z, Buck A, Harrison L, Schriever SC, et al. Light sheet fluorescence microscopy guided MALDI-imaging mass spectrometry of cleared tissue samples. Sci Rep. 2020;10:14461. doi:10.1038/s41598-020-71465-1.

19. Rabe J-H, A Sammour D, Schulz S, Munteanu B, Ott M, Ochs K, et al. Fourier Transform Infrared Microscopy Enables Guidance of Automated Mass Spectrometry Imaging to Predefined Tissue Morphologies. Sci Rep. 2018;8:313. doi:10.1038/s41598-017-18477-6.

20. Patterson NH, Tuck M, van de Plas R, Caprioli RM. Advanced Registration and Analysis of MALDI Imaging Mass Spectrometry Measurements through Autofluorescence Microscopy. Anal Chem. 2018;90:12395–403. doi:10.1021/acs.analchem.8b02884.

21. Croslow SW, Trinklein TJ, Sweedler JV. Advances in multimodal mass spectrometry for single-cell analysis and imaging enhancement. FEBS Lett. 2024;598:591–601. doi:10.1002/1873-3468.14798.

22. Gachumi G, Purves RW, Hopf C, El-Aneed A. Fast Quantification Without Conventional Chromatography, The Growing Power of Mass Spectrometry. Anal Chem. 2020;92:8628–37. doi:10.1021/acs.analchem.0c00877.

23. Yuan J, Li X, Shen X, Xiong P, Zhu N, Ye Y, Liu J. Comprehensive Metabolite Profiling in Single-Cell Systems via Dual-Modal MALDI-Mass Spectrometry Imaging. Anal Chem. 2025;97:8729–37. doi:10.1021/acs.analchem.4c05480.

24. Ngai YT, Lau D, Mittal P, Hoffmann P. Mini Review: Highlight of Recent Advances and Applications of MALDI Mass Spectrometry Imaging in 2024. Anal Sci Adv. 2025;6:e70016. doi:10.1002/ansa.70016.

25. Schrimpe-Rutledge AC, Codreanu SG, Sherrod SD, McLean JA. Untargeted Metabolomics Strategies-Challenges and Emerging Directions. J Am Soc Mass Spectrom. 2016;27:1897–905. doi:10.1007/s13361-016-1469-y.

26. Kind T, Fiehn O. Seven Golden Rules for heuristic filtering of molecular formulas obtained by accurate mass spectrometry. BMC Bioinformatics. 2007;8:105. doi:10.1186/1471-2105-8-105.

27. Sun Z, Wang F, Liu Y, Deng B, Ren R, Wang Y, et al. Recent strategies for improving MALDI mass spectrometry imaging performance towards low molecular weight compounds. TrAC Trends in Analytical Chemistry. 2024;175:117727. doi:10.1016/j.trac.2024.117727.

28. Tiquet M, La Rocca R, Kirnbauer S, Zoratto S, van Kruining D, Quinton L, et al. FT-ICR Mass Spectrometry Imaging at Extreme Mass Resolving Power Using a Dynamically Harmonized ICR Cell with 1ω or 2ω Detection. Anal Chem. 2022;94:9316–26. doi:10.1021/acs.analchem.2c00754.

29. Thompson CJ, Witt M, Forcisi S, Moritz F, Kessler N, Laukien FH, Schmitt-Kopplin P. An Enhanced Isotopic Fine Structure Method for Exact Mass Analysis in Discovery Metabolomics: FIA-CASI-FTMS. J Am Soc Mass Spectrom. 2020;31:2025–34. doi:10.1021/jasms.0c00047.

30. Popov IA, Nagornov K, Vladimirov GN, Kostyukevich YI, Nikolaev EN. Twelve million resolving power on 4.7 T Fourier transform ion cyclotron resonance instrument with dynamically harmonized cell--observation of fine structure in peptide mass spectra. J Am Soc Mass Spectrom. 2014;25:790–9. doi:10.1007/s13361-014-0846-7.

31. Kostyukevich YI, Vladimirov GN, Nikolaev EN. Dynamically harmonized FT-ICR cell with specially shaped electrodes for compensation of inhomogeneity of the magnetic field. Computer simulations of the electric field and ion motion dynamics. J Am Soc Mass Spectrom. 2012;23:2198–207. doi:10.1007/s13361-012-0480-1.

32. Marshall AG, Hendrickson CL. High-resolution mass spectrometers. Annu Rev Anal Chem (Palo Alto Calif). 2008;1:579–99. doi:10.1146/annurev.anchem.1.031207.112945.

33. Palmer A, Phapale P, Chernyavsky I, Lavigne R, Fay D, Tarasov A, et al. FDR-controlled metabolite annotation for high-resolution imaging mass spectrometry. Nat Methods. 2017;14:57–60. doi:10.1038/nmeth.4072.

34. Hohenester UM, Barbier Saint-Hilaire P, Fenaille F, Cole RB. Investigation of space charge effects and ion trapping capacity on direct introduction ultra-high-resolution mass spectrometry workflows for metabolomics. J Mass Spectrom. 2020;55:e4613. doi:10.1002/jms.4613.

35. Nikolaev EN, Kostyukevich YI, Vladimirov GN. Fourier transform ion cyclotron resonance (FT ICR) mass spectrometry: Theory and simulations. Mass Spectrom Rev. 2016;35:219–58. doi:10.1002/mas.21422.

36. Wong RL, Amster IJ. Experimental Evidence for Space-Charge Effects between Ions of the Same Mass-to-Charge in Fourier-Transform Ion Cyclotron Resonance Mass Spectrometry. Int J Mass Spectrom. 2007;265:99–105. doi:10.1016/j.ijms.2007.01.014.

37. Masselon C, Tolmachev AV, Anderson GA, Harkewicz R, Smith RD. Mass measurement errors caused by 'local" frequency perturbations in FTICR mass spectrometry. J Am Soc Mass Spectrom. 2002;13:99–106. doi:10.1016/S1044-0305(01)00333-6.

38. The M, Samaras P, Kuster B, Wilhelm M. Reanalysis of ProteomicsDB Using an Accurate, Sensitive, and Scalable False Discovery Rate Estimation Approach for Protein Groups. Mol Cell Proteomics. 2022;21:100437. doi:10.1016/j.mcpro.2022.100437.

39. Grgic A, Nagornov KO, Kozhinov AN, Michael JA, Anthony IGM, Tsybin YO, et al. Ultrahigh-Mass Resolution Mass Spectrometry Imaging with an Orbitrap Externally Coupled to a High-Performance Data Acquisition System. Anal Chem. 2024;96:794–801. doi:10.1021/acs.analchem.3c04146.

40. Vandergrift GW, Zemaitis KJ, Veličković D, Lukowski JK, Paša-Tolić L, Anderton CR, Kew W. Experimental Assessment of Mammalian Lipidome Complexity Using Multimodal 21 T FTICR Mass Spectrometry Imaging. Anal Chem. 2023;95:10921–9. doi:10.1021/acs.analchem.3c00518.

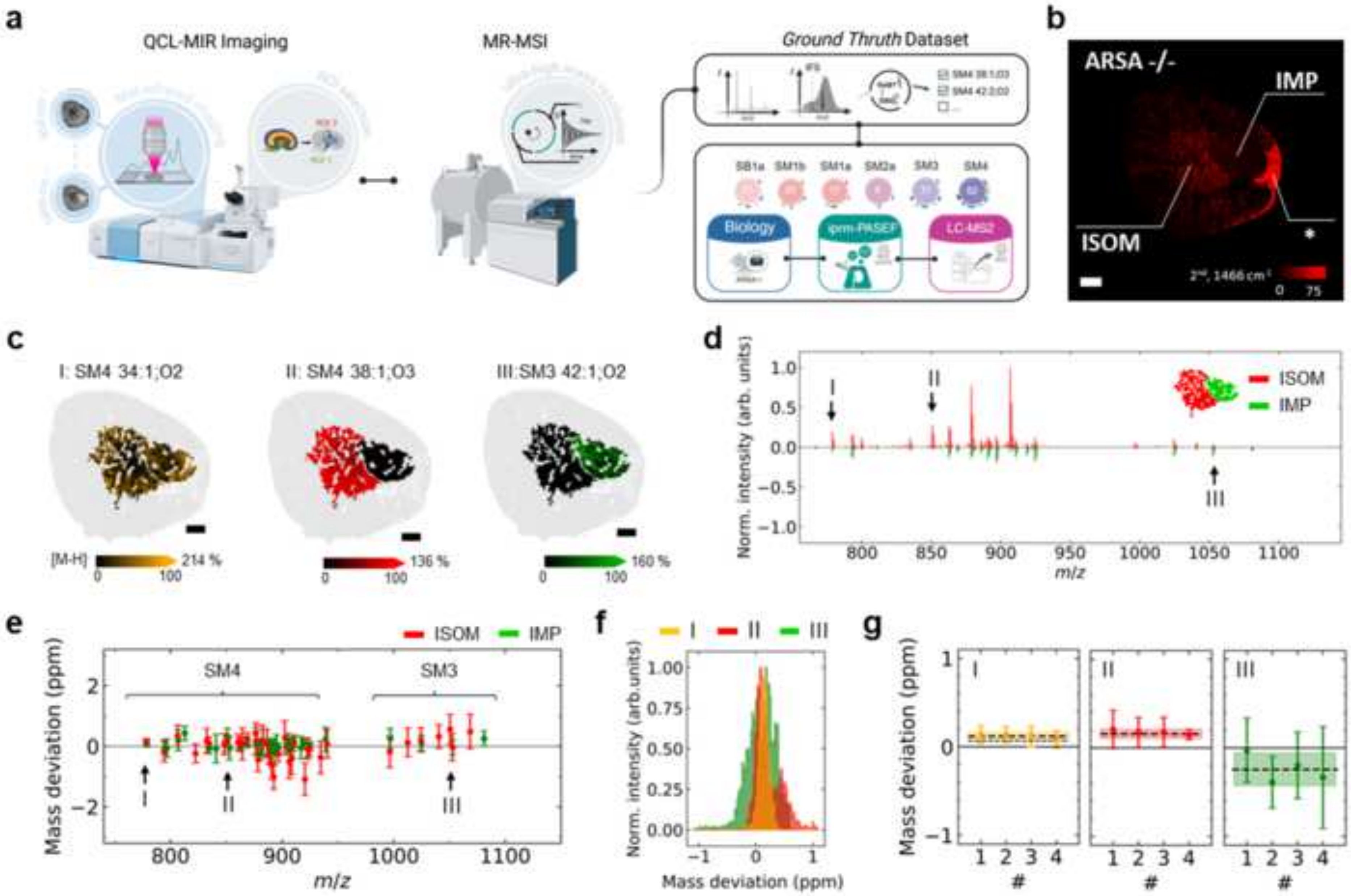
a
QCL-MIR Imaging
MR-MSI
Ground Thruth Dataset
SB1a SM1b SM1a SM2a SM3 SM4
Biology
iprm-PASEF
LC-MS2
b
ARSA -/-
IMP
ISOM
c
I: SM4 34:1;O2
II: SM4 38:1;O3
III:SM3 42:1;O2
[M-H]
214 %
136 %
160 %
d
Norm. intensity (arb. units)
ISOM
IMP
m/z
e
Mass deviation (ppm)
SM4
SM3
m/z
f
Norm. intensity (arb.units)
Mass deviation (ppm)
g
Mass deviation (ppm)
#

Figure2

Click here to access/download;Figure;Fig2.png

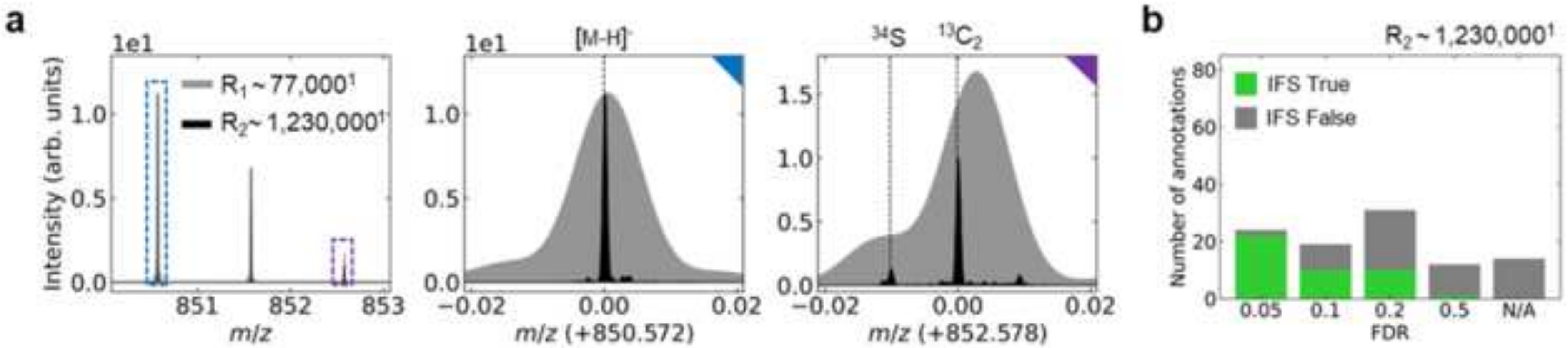

Supplementary Dataset 1

Click here to access/download
Supplementary Material
Supplementary_Dataset_1_FTICR_1s.rar

Supplementary Dataset 2

Click here to access/download
Supplementary Material
Supplementary_Dataset_2_FTICR_16s.rar

Supplementary Dataset 3

Click here to access/download

Supplementary Material

List_of_theoretical_sulfatides_with_isotopes_NEW.xlsx

Supplementary Information

Click here to access/download
Supplementary Material
Gigascience_QCL-MRMS_Suppl.docx

Cover Letter

Click here to access/download;Personal Cover;Gigascience_QCL-MRMS_Cover_Letter.DOCX

Center for Mass Spectrometry and Optical Spectroscopy

Center for Mass Spectrometry and Optical Spectroscopy (CeMOS)

Carsten Hopf, PhD

Paul-Wittsack-Str. 10
68163 Mannheim, Germany
Tel. +49 621 292-6802
Fax. +49 621 292-6-6802-1
Email: c.hopf@hs-mannheim.de

Date: 25.07.2025

To:

Hans Zauner, PhD

Editor, GigaScience Press Germany

Submission of Gruber et al. to *GigaScience*

Dear Dr. Zauner,

It is our pleasure to submit our manuscript entitled **"A sulfatide-centered ultra-high resolution magnetic resonance MALDI imaging benchmark dataset for MS1-based lipid annotation tools"** for consideration as a Data Note in GigaScience.

We believe that our study and the underlying datasets will be of substantial interest to the readers of *GigaScience* for several reasons:

1. We introduce a new data acquisition workflow for mass spectrometry imaging (MSI) that combines quantum cascade laser mid-infrared (QCL-MIR) imaging microscopy with magnetic resonance MSI (MR-MSI). Using this workflow, we generated an ultra-high resolution spatial lipidomics dataset with a resolution of ~1,230,000 and compare it with a more typical dataset at 77,000 resolution.

2. We address the critical lack of ground truth datasets needed to support computational advancements in MSI, thus offering a unique community resource to drive future progress in the field. To this end, we provide these two well-characterized, biologically relevant, sulfatide-centered MSI datasets derived from an arylsulfatase A mouse model. The ultra-high resolution QCL-MIR imaging-guided MR-MSI dataset enables isotopic fine structure analysis.

3. Our Data Note offers detailed manual annotations and comparisons with decoy database-controlled computational annotations, emphasizing both the advantages and current drawbacks of automated annotation methods. All annotations are validated by MS/MS structure elucidation approaches. This resource will aid further development and validation of techniques, and we expect it to become a reference for future research into sulfatide metabolism and its spatial regulation as well as for computational MS1 annotations in lipidomics.

4. Consistent with GigaScience's commitment to open science, we will supply all raw data, annotation files, and comprehensive protocols to guarantee transparency, reproducibility, and optimal reusability by the research community.

Furthermore, the distinctive integration of biological significance, technical variability, and elevated annotation confidence renders these datasets exceptionally valuable for progressing computational methods designed to address the intrinsic challenges associated with MS1-level MSI data.

To our knowledge, this is the first openly accessible biological MR-MSI dataset of its kind, and we believe it will help address the recognized lack of reliable ground truth data in the field. This manuscript is original, not published elsewhere, and not under review by any other journal. All authors have approved it and agree to submit it to GigaScience. Any potential conflicts of interest are disclosed within the manuscript. We hope you find our Data Note relevant and suitable for publication in GigaScience. Thank you for your consideration.

These individuals could be suitable reviewers:

1. Chris Anderton, PhD. Environmental Molecular Sciences Laboratory and Biological Sciences Division, Pacific Northwest National Laboratory, Richland, Washington 99354, United States.
   Email: christopher.anderton@pnnl.gov.
2. Michael Becker, PhD. Boehringer Ingelheim Pharma GmbH & Co. KG, Birkendorfer Str. 65, 88397 Biberach
   Email: michael_4.becker@boehringer-ingelheim.com
3. Andrew Palmer, PhD. GSK Research & Development, Stevenage, UK.
   Email: Andrew.x.palmer@gsk.com
4. Prof. Per E. Andrén. Department of Pharmaceutical Biosciences, Spatial Mass Spectrometry, Science for Life Laboratory, Uppsala University, Uppsala SE-75124, Sweden
   Email: per.andren@uu.se

Yours sincerely,

Carsten

Professor Carsten Hopf, PhD

Head, Center of Mass Spectrometry and Optical Spectroscopy (CeMOS)
