# Supplementary material for: A sulfatide-centered ultra-high-resolution magnetic resonance MALDI imaging benchmark dataset for MS1-based lipid annotation tools
Source: Gigascience. 2025 Dec 9;14:giaf150. doi: 10.1093/gigascience/giaf150 (PMC12766628; doi:10.1093/gigascience/giaf150)
Supplement: giaf150_GIGA-D-25-00298_Revision_1 [file giaf150_giga-d-25-00298_revision_1.pdf]

GigaScience

A sulfatide-centered ultra-high resolution magnetic resonance MALDI imaging benchmark dataset for MS1-based lipid annotation tools

--Manuscript Draft--

**Manuscript Number:**	GIGA-D-25-00298R1
**Full Title:**	A sulfatide-centered ultra-high resolution magnetic resonance MALDI imaging benchmark dataset for MS1-based lipid annotation tools
**Article Type:**	Data Note
**Funding Information:**	Bundesministerium für Bildung und Forschung (12FH8I05IA) — Prof. Dr. Carsten Hopf<br>Ministerium für Wissenschaft, Forschung und Kunst Baden-Württemberg (Mittelbauprogramm) — Prof. Dr. Carsten Hopf<br>Deutsche Forschungsgemeinschaft (262133997) — Prof. Dr. Carsten Hopf<br>Deutsche Forschungsgemeinschaft (497984836) — Prof. Dr. Carsten Hopf
**Abstract:**	Spatial 'omics techniques are indispensable for studying complex biological systems and for the discovery of spatial biomarkers. While several current matrix-assisted laser desorption/ionization (MALDI) mass spectrometry imaging (MSI) instruments are capable of localizing numerous metabolites at high spatial and spectral resolution, the majority of MSI data is acquired at the MS1 level only. Assigning molecular identities based on MS1 data presents significant analytical and computational challenges, as the inherent limitations of MS1 data preclude confident annotations beyond the sum formula level. To enable future advancements of computational lipid annotation tools, well-characterized benchmark - or ground truth - datasets are crucial, which exceed the scope of synthetic data or data derived from mimetic tissue models. To this end, we provide two sulfatide-centered, biology-driven magnetic resonance MSI (MR-MSI) datasets at different mass resolving powers that characterize lipids in a mouse model of human metachromatic dystrophy. This data includes an ultra-high-resolution (R ~1,230,000) quantum cascade laser mid-infrared imaging-guided MR-MSI dataset that enables isotopic fine structure analysis and therefore enhances the level of confidence substantially. To highlight the usefulness of the data, we compared 118 manual sulfatide annotations with the number of decoy database-controlled sulfatide annotations performed in Metaspace (67 at FDR < 10%). Overall, our datasets can be used to benchmark annotation algorithms, validate spatial biomarker discovery pipelines, and serve as a reference for future studies that explore sulfatide metabolism and its spatial regulation.
**Corresponding Author:**	Carsten Hopf, Ph.D.<br>Mannheim University of Applied Sciences: Hochschule Mannheim<br>MANNHEIM, GERMANY
**Corresponding Author Secondary Information:**	
**Corresponding Author's Institution:**	Mannheim University of Applied Sciences: Hochschule Mannheim
**Corresponding Author's Secondary Institution:**	
**First Author:**	Lars Gruber
**First Author Secondary Information:**	
**Order of Authors:**	Lars Gruber<br>Stefan Schmidt<br>Thomas Enzlein<br>Carsten Hopf

Powered by Editorial Manager® and ProduXion Manager® from Aries Systems Corporation

Order of Authors Secondary Information:	
Response to Reviewers:	Gruber et al. GIGA-D-25-00298<br><br>Point-for-Point Reply to Reviewers<br><br>A sulfatide-centered ultra-high resolution magnetic resonance MALDI imaging benchmark dataset for MS1-based lipid annotation tools<br><br><br>Revision2<br>1.Editorial Comments2<br>2.Reviewer #12<br>2.1 Provision of the “Ground Truth" Annotation List:3<br>2.2 Strengthening the "Ground Truth" Justification:3<br>2.3 Deeper Analysis of Automated Annotation Discrepancies:4<br>2.4 Minor Revisions5<br>2.5 Closing Comment7<br>3.Reviewer #2:7<br>3.1 Comment 17<br>3.2 Comment 28<br>3.3 Comment 39<br>3.4 Comment 49<br>3.5 Comment 510<br>3.6 Final Comment10<br><br><br>Revision<br><br>1.Editorial Comments<br>Your manuscript "A sulfatide-centered ultra-high resolution magnetic resonance MALDI imaging benchmark dataset for MS1-based lipid annotation tools" (GIGA-D-25-00298) has been assessed by our reviewers. Although it is of interest, we are unable to consider it for publication in its current form. The reviewers have raised a number of points which we believe would improve the manuscript and may allow a revised version to be published in GigaScience.<br><br>Their reports, together with any other comments, are below. Please also take a moment to check our website at https://www.editorialmanager.com/giga/ for any additional comments that were saved as attachments.<br><br>We thank the Editor and the anonymous reviewers for their careful assessment of our manuscript and for the constructive feedback provided. We greatly appreciate the opportunity to revise and strengthen our work in light of these valuable comments. The reviewer input has identified key areas for clarification and enhancement that will substantially improve both the scientific rigor and clarity of our data note.<br>Below, we provide detailed point-for-point responses to each reviewer comment, with corresponding manuscript revisions highlighted. We believe that these revisions directly address all concerns raised and bring the manuscript into alignment with GigaScience's standards for data transparency and reproducibility.<br><br>In addition, please register any new software application in the bio.tools and SciCrunch.org databases to receive RRID (Research Resource Identification Initiative ID) and biotoolsID identifiers, and include these in your manuscript. Computational workflows should be registered in workflowhub.eu and the DOIs cited in the relevant places in the manuscript. These will facilitate tracking, reproducibility and re-use of your tool.

Powered by Editorial Manager® and ProduXion Manager® from Aries Systems Corporation

Does not apply – as outlined in code availability statement.

All web links and URLs should be given a reference number and included in the reference list rather than within the text of the manuscript. Please remove the URLs, cite them as reference and adjust the order of the reference accordingly. Except for "Availability of Source Code and Requirements"

Two web links and URL were removed from the manuscript and moved to references as reference 30 and 37.

2.Reviewer #1
This manuscript by Gruber et al. provides a Data Note detailing a high-value, sulfatide-focused benchmark dataset for the mass spectrometry imaging (MSI) community. The project is well thought out, technically advanced, and directly meets a major need for biologically relevant, deeply characterized ground-truth data to test MS1-level metabolite annotation software. It is a big technical achievement to create an ultra-high-resolution dataset (R~1,230,000) with a 7T FT-ICR instrument. The use of isotopic fine structure (IFS) to boost annotation confidence is a major strength. Using QCL-MIR imaging strategically to guide the MSI acquisition is a smart and effective way to do things. It's great that the authors are committed to FAIR principles.
The writing in the manuscript is excellent, and the data is very good. It makes a big difference in the field. There are, however, several changes that should be made to make it clearer, more scientifically complete, and more useful as a stand-alone benchmark resource for the community. The following points are given to help make the manuscript stronger for publication.

We sincerely appreciate Reviewer 1's thoughtful and encouraging assessment of our work. Their recognition of the technical achievement and the potential impact of this benchmark dataset for the MSI community is invaluable, and their constructive suggestions for strengthening the manuscript will significantly improve its clarity and utility.

2.1 Provision of the “Ground Truth" Annotation List:
The benchmark dataset is the most important part of this Data Note. The manuscript's supplementary information, on the other hand, doesn't seem to have the final, curated list of manual annotations that make up the "ground truth." For this dataset to be truly reusable for benchmarking third-party software, it needs another table. This table should show all of the manually annotated sulfatides for each replicate, along with their experimental m/z, proposed sum formula, lipid annotation, mass error (ppm), and a way to tell if IFS was used to confirm them.

We thank Reviewer 1 for highlighting this critical gap. We agree that providing a comprehensive, curated ground-truth annotation table is essential for the utility of this dataset as a benchmark. We have added new supplementary datasets 4 and 5. Here, we included a list of all manually annotated sulfatides for each replicate, including the following columns: proposed sum formula, lipid annotation (including acyl chain composition), m/z measured, mass deviation (ppb), and an indicator denoting whether isotopic fine structure (IFS) was used to confirm the annotation (new Supplementary Dataset 4). In addition, we provide a list of all sulfatides we annotated in metaspace with our custom database and at which FDR they were annotated for each replicate (new Supplementary Dataset 5). These files will serve as the definitive reference for benchmarking third-party MS1-level annotation software and will be prominently linked in the main manuscript and data repository to ensure discoverability. We believe this addition will substantially enhance the reusability and scientific value of the dataset for the community.

2.2 Strengthening the "Ground Truth" Justification:
The manuscript depends on an earlier publication (Ref) to validate the sulfatide structures using MS/MS. It is acceptable to reference previous work, but a benchmark Data Note should be as self-sufficient as possible. Please add a short paragraph to the "Data Validation and Quality Control" section that sums up the main MS/MS fragmentation evidence from Ref that backs up the sulfatide identifications. This will give users of the dataset a more complete and clear chain of evidence.

Powered by Editorial Manager® and ProduXion Manager® from Aries Systems Corporation

We thank Reviewer 1 for this important suggestion regarding data self-sufficiency. We agree that a benchmark Data Note should provide readers with a complete chain of evidence without requiring extensive reference to prior publications. We expanded the "Data Validation and Quality Control" section to include a concise summary of the key MS/MS fragmentation patterns and diagnostic ions that support the sulfatide identifications, referencing the earlier publication while distilling the most critical structural evidence. This addition will enable users of the dataset to understand the rationale behind our identifications directly within the manuscript and enhance the utility of the benchmark resource for the community.

2.3 Deeper Analysis of Automated Annotation Discrepancies:
The comparison with Metaspace shows how important this dataset is by showing that even a top-of-the-line tool can't annotate 14 high-confidence sulfatides. The discussion needs to be longer so that it can look at why these failures could be happening. Please explain why Metaspace's scoring algorithm, which only looks at the four most intense isotopic peaks, might not work well with this kind of ultra-high-resolution data where low-intensity IFS peaks (like 34 S) are very important. Talking about how future algorithms could make better use of this information would make the paper much more useful and help with the development of new tools.

We thank Reviewer 1 for this insightful suggestion. We expanded the Discussion section to provide a thorough analysis of the comparison between manual and automated (Metaspace) annotation, emphasizing that Metaspace performs robustly on this benchmark dataset and that the unannotated species represent a data limitation rather than an algorithmic limitation.
Metaspace performed well overall on this benchmark dataset. At a 10% FDR threshold, Metaspace successfully identified 43 of the 91 manually annotated sulfatides in the ultra-high-resolution dataset, demonstrating effective performance for species with complete or near-complete isotopic envelopes above noise thresholds. The algorithm's isotopic pattern matching and mass accuracy-based scoring framework functions reliably within its design specifications and represents a valuable resource for high-throughput MSI annotation workflows.
The 14 unannotated sulfatides represent inherent data limitations, not algorithmic deficiencies. These species are low-abundance compounds for which only the monoisotopic $[M-H]^-$ peak was detectable above the signal-to-noise threshold, while the isotopic envelope peaks fell below detection limits. This represents a fundamental measurement constraint: when the complete isotopic envelope is not experimentally resolvable due to low abundance, annotation based on isotopic pattern matching becomes physically impossible, regardless of algorithm sophistication. Metaspace appropriately rejects such species rather than making spurious assignments based on incomplete isotopic data—a conservative approach that prioritizes annotation confidence. Manual recovery of these species relied on monoisotopic m/z matching against the custom reference database combined with external validation (Gruber et al. 2025), exploiting orthogonal validation information unavailable to automated algorithms processing only MS1 data.
However, we identified one genuine algorithmic opportunity in the ultra-high-resolution regime. Notably, 37% of our manual annotations in the first replicate (34 of 91) and 40% in the second replicate (39 of 97) were substantiated by ^{34}S isotopic fine structure (IFS) peak detection. This demonstrates that diagnostic IFS information is frequently available in this dataset but underutilized by Metaspace, which restricts isotopic envelope matching to the four most intense peaks. While this limitation does not prevent successful annotation of species with complete isotopic envelopes, the inability to leverage IFS peaks for confidence stratification represents a missed opportunity: high-confidence annotations supported by robust IFS detection remain algorithmically indistinguishable from lower-confidence mass-error-based assignments.
We included a prospective discussion of algorithmic refinements that could enhance performance in ultra-high-resolution workflows, including: (a) expansion of isotopic peak matching to encompass all resolved peaks above instrument-specific signal-to-noise thresholds, enabling full exploitation of IFS information when present; (b) explicit confidence stratification that explicitly correlates annotation reliability with IFS detectability; (c) integration of machine-learning classifiers trained on empirical IFS signatures to improve discrimination between isomeric sulfatides; and (d) incorporation of complementary analytical dimensions (ion mobility, MS/MS fragmentation) where

Powered by Editorial Manager® and ProduXion Manager® from Aries Systems Corporation

available. These additions position this dataset as a valuable training and validation resource for algorithm refinement without diminishing the demonstrated effectiveness of current-generation tools like Metaspace.

2.4 Minor Revisions

2.4.1 Clarification of Table 1:
The row headers for the R2 dataset ("all" vs. "QCL-MIR-guided") are slightly confusing, as all R2 data is QCL-MIR-guided. Please revise these for clarity (e.g., "Total Annotations in ROIs" and "Annotations with Confirmed IFS Evidence").

We thank Reviewer 1 for this important suggestion regarding table clarity. We revised the row labels for improved transparency and precision. The headers were changed from "all" and "QCL-MIR-guided" to "Total Annotations in ROIs" and "Annotations with Confirmed IFS Evidence," respectively. These revised labels explicitly communicate the distinction between the complete set of manually identified sulfatides within the regions of interest and the subset of those annotations that were specifically validated through isotopic fine structure peak detection. This revision eliminates ambiguity and enhances the interpretability of the benchmark dataset for end users.

2.4.2 Definition of "Internal Error":
The legend for Figure 1g should include a brief definition or reference for how "internal error" was calculated to ensure the metric is fully understood.

We thank Reviewer 1 for this suggestion to enhance clarity regarding the "internal error" metric in Figure 1g. We revised the figure legend to explicitly define the calculation method. The internal error was calculated based on Equation 13 from Roux et al., Eur. Phys. J. D (2013) 67:75 (https://link.springer.com/article/10.1140/epjd/e2013-40110-x), which quantifies the consistency of mass measurement accuracy of each identified species. We added the following text to the Figure 1g legend: "Internal error was calculated according to Eq. 13 in Roux et al. [reference number], reflecting the deviation between observed and theoretical isotopic mass distributions." This addition ensures that readers can readily interpret the metric and reproduce the quality assessment independently.

2.4.3 Confirmation of Database Contents:
In the Methods section, please add a sentence explicitly confirming that all manually annotated sulfatide species were included in the custom database file used for the Metaspace analysis. This is a crucial detail for a fair comparison.

We thank Reviewer 1 for identifying this critical detail regarding methodological transparency. We agree that explicit confirmation of database completeness is essential for ensuring a fair and reproducible comparison between manual and algorithmic annotations. We added a clarifying sentence to the Methods section explicitly confirming that all 91 (first replicate) and 97 (second replicate) manually annotated sulfatide species were included in the custom database file (Supplementary Dataset 3) supplied to Metaspace for the analysis. This statement ensures that the comparison is conducted on equal footing and that Metaspace's apparent annotation failures cannot be attributed to missing entries in the reference database. This addition reinforces the validity of our findings regarding the algorithmic limitations of Metaspace and strengthens the benchmark value of our dataset.
2.4.4 Explicit Statement of Dataset Limitations:
In the "Re-use Potential" section, it would be beneficial to explicitly state the inherent trade-off of the ultra-high-resolution approach. Please add a sentence acknowledging that the dataset is optimized for high-confidence annotation and that this comes at the cost of reduced sensitivity and comprehensive spatial coverage compared to a standard MSI experiment.

We thank Reviewer 1 for this important suggestion regarding the transparency of dataset limitations. We agree that explicitly stating the inherent trade-offs of the ultra-high-resolution approach strengthens the manuscript and provides critical context for end users considering this dataset for their work. We added a clarifying statement to the "Re-use Potential" section that explicitly acknowledges the methodological trade-off: whilst the QCL-MIR-guided ultra-high-resolution approach achieves substantially

elevated identification confidence through isotopic fine structure analysis and reduced ion current to minimize space charge effects, this optimization comes at the cost of reduced ion abundance and spatial coverage compared to conventional whole-tissue MSI experiments. Specifically, we noted that the number of sulfatide annotations in the QCL-MIR-guided ultra-high-resolution dataset (91 and 97 for the 60-week replicates) was lower than in the conventional non-guided whole-tissue approach (118 and 115), reflecting the intentional trade-off between analytical depth and comprehensive tissue coverage. This addition ensures that prospective users understand both the strengths and practical limitations of the dataset and can make informed decisions regarding its applicability to their specific research objectives.

2.4.5 Link to Custom Database:
The Methods section mentions the creation of a custom database of 780 theoretical sulfatides. Please explicitly state in the text that this database is available as Supplementary Dataset 3.

We thank Reviewer 1 for this suggestion to enhance the accessibility and traceability of the custom database. We agree that explicitly linking the database reference to its location in the supplementary materials strengthens reproducibility and facilitates access for users. We verified that the Methods section already contains explicit reference to the database availability as Supplementary Dataset 3 in the sentence: " A custom reference database integrating LipidMaps with 780 theoretically derived sulfatide structures, available as Supplementary Dataset 3, was constructed for Metaspace analysis." This statement ensures that readers can readily locate and access the custom database used for the analysis, supporting the reproducibility and transparency objectives of the study.

2.5 Closing Comment
Addressing these points will significantly enhance the manuscript's value and ensure its lasting impact as a key resource for the computational mass spectrometry community.

We appreciate Reviewer 1's constructive feedback, which has substantially strengthened the manuscript's scientific rigor, methodological transparency, and clarity regarding the benchmark dataset's utility. We are confident that these revisions address all concerns raised and position the manuscript as a robust, lasting resource for the computational mass spectrometry community.

3.Reviewer #2:
I believe that the dataset produced is a great contribution to the community. My major concerns are as follows:
We thank Reviewer 2 for the encouraging assessment of the dataset's contribution and for the detailed constructive feedback that has strengthened the manuscript. We address the major concerns below.

3.1 Comment 1
The data described is good but please clarify how would be solution the discrepancy between the manual annotations and the computational annotations and annotations quality for he sulfatide-centered MSI dataset, challenges?
We appreciate this question, which prompted us to clarify the discussion regarding discrepancies between manual and computational annotations. This concern aligns substantively with Reviewer #1's comment regarding the limitations of the Metaspace annotation algorithm (Comment 2.3 “Deeper Analysis of Automated Annotation Discrepancies”), and we have expanded the Discussion section comprehensively to address both perspectives.
The core challenges underlying the manual-computational divergence are fundamentally rooted in algorithmic design constraints and represent important considerations for future tool development. The primary discrepancy arises from Metaspace's scoring methodology, which restricts isotopic envelope matching to the four most intense peaks, thereby failing to exploit diagnostic isotopic fine structure (IFS) information—particularly ^{34}S isotopologues—that was readily accessible in our manually curated dataset. This algorithmic limitation means that Metaspace cannot discriminate between structurally distinct sulfatides that exhibit identical or near-

identical precursor m/z values but differ in their isotopic signatures. Conversely, our manual annotation workflow leveraged the complete isotopic fingerprint available at 1,230,000 mass resolving power, enabling unambiguous identification of species that computational algorithms currently cannot resolve.
Beyond algorithmic limitations, we identified three additional interrelated challenges: (1) signal intensity dependence, wherein low-abundance sulfatides exhibit attenuated IFS peak intensities that may fall below detection thresholds, rendering them inaccessible to IFS-based annotation strategies; (2) database completeness, which we addressed directly by ensuring all manually annotated species were represented in the custom database supplied to Metaspace; and (3) confidence stratification, wherein current algorithms provide no mechanism to distinguish high-confidence annotations supported by robust IFS detection from those inferred from precursor m/z alone.
To bridge this discrepancy, we propose that future annotation algorithms should implement the following innovations: (a) expansion of isotopic envelope matching to encompass all resolved peaks above instrument-specific signal-to-noise thresholds; (b) integration of machine-learning classifiers trained on empirical IFS signatures across heterogeneous signal intensity ranges; (c) explicit confidence scoring that correlates annotation reliability with IFS detectability; and (d) incorporation of complementary analytical dimensions (ion mobility, MS/MS fragmentation) to adjudicate between isotopically indistinguishable candidates.
In response to both reviewers' comments, we have substantially expanded the Discussion section to articulate these pathways for algorithmic advancement. This benchmark dataset provides the ground truth and diverse signal intensity distribution necessary to validate and refine such approaches, thereby directly addressing the manual-computational discrepancy through iterative algorithmic improvement.

3.2 Comment 2
Please remove too old references unless they are pioneer and replace with the new ones.

We appreciate this helpful suggestion regarding the currency and relevance of our reference list. We have carefully reviewed all cited literature and updated the manuscript to emphasize recent publications from 2020 onward, while selectively keeping foundational references that establish essential theoretical and methodological frameworks. Specifically, we removed outdated references that lack current relevance: Masselon et al. (2002) and Marshall & Hendrickson (2008) were removed because they addressed calibration and FT-ICR theory from the early 2000s and have been superseded by modern approaches. Kind & Fiehn (2007) was replaced with current metabolomic annotation strategies reflecting best practices today. Thompson et al. (2020), although focused on mass accuracy, used a FIA-CASI-FTMS workflow that is not directly applicable to our MALDI imaging context and was therefore replaced with Popov et al. (2014), which provides foundational work on isotopic fine structure analysis using dynamically harmonized FT-ICR cells—directly relevant to the ultra-high-resolution lipid characterization presented in this study.
Critically, we integrated Pieczonka et al. (2025) (10.1021/acs.analchem.5c00488) as a contemporary reference addressing modern FT-ICR harmonization and space charge mitigation strategies in ultra-high-resolution instruments. Additionally, we replaced Nikolaev et al. (2016) with Cochran et al. (2024) (https://doi.org/10.3390/biomedicines12081786), a comprehensive review on FT-ICR-MS applications in metabolomics and theoretical frameworks.
Conversely, we retained seminal older references—such as Hess et al. (1996), which introduced the ARSA-deficient mouse model employed in this study, Shi et al. (1998) on the first demonstration of isotopic fine structure resolution in biological molecules, and Wong & Amster (2007) on fundamental space charge physics—because these papers establish foundational biological models, methodological innovations, and physical principles that remain essential references in contemporary FT-ICR and mass spectrometry literature and directly underpin the technical and biological innovations presented in this work. This balanced approach ensures that the manuscript reflects state-of-the-art developments whilst maintaining essential historical and theoretical context.

3.3 Comment 3
Please try to add some of figures as supplementary instead of text,

Powered by Editorial Manager® and ProduXion Manager® from Aries Systems Corporation

	We appreciate this comment, though we note that the phrasing could be interpreted in multiple ways—specifically, whether the suggestion is to move main text figures to supplementary material or conversely to elevate supplementary figures to the main text for enhanced clarity. To provide the most beneficial revision, we interpret your comment as a request to move key supplementary figures into the main manuscript to improve readability and reinforce the manuscript narrative with visual support.<br>In response, we have reorganized the figure presentation to strengthen the main narrative. Specifically, the old Supplementary Figure 7 (showing 77,000 R annotation results) is now integrated into the expanded updated Figure 2b alongside the 1,230,000 R data, providing direct visual comparison of annotation performance across both mass resolutions within the main text. Additionally and line with the first Reviewer (Comment 2.1) we created a new Supplementary Dataset 4 that provides comprehensive elaboration of mass accuracy metrics and isotopic fine structure (IFS) detection across all n=4 biological replicates, further strengthening the quantitative validation of our annotation quality assessment and demonstrating consistency across the full dataset. These reorganizations allow readers to immediately contextualize the superior performance of ultra-high mass resolving power and the robustness of our IFS-based validation strategy without requiring extensive supplementary material consultation, thereby enhancing manuscript clarity and data accessibility.<br>If your comment instead refers to relocating main text figures to supplementary material, we welcome further clarification so that we may optimize the figure arrangement accordingly. Our priority is to present this benchmark dataset in the most comprehensible format for the readership.<br><br>3.4 Comment 4<br>algorithm is not fully optimized or not?<br><br>We appreciate this question regarding algorithmic optimization. However, we note that the phrasing of this comment could be interpreted in multiple ways—specifically, whether the question pertains to the state of optimization of the Metaspace algorithm itself, the optimization of our custom database parameters for Metaspace analysis, or the optimization of our manual annotation workflow. To provide the most comprehensive response, we interpret your comment as addressing the optimization status of the Metaspace algorithm for sulfatide annotation.<br>This topic was extensively addressed in our response to Reviewer #1, Comment 2.3 (Deeper Analysis of Automated Annotation Discrepancies), where we comprehensively analyzed Metaspace's performance on this benchmark dataset.<br>To summarize: Metaspace is well-optimized for its core design specifications, successfully identifying 43 of 91 manually annotated sulfatides at a 10% FDR threshold. The 14 unannotated species represent a measurement limitation—low-abundance compounds with incomplete isotopic envelopes where only the monoisotopic $[M-H]^-$ peak exceeds the noise threshold. This is fundamentally a data constraint, not an algorithmic deficiency. Metaspace's conservative approach of rejecting incomplete datasets is scientifically sound.<br>However, we identified one genuine optimization opportunity: expanding isotopic envelope matching beyond the four most intense peaks to exploit ^{34}S isotopic fine structure information for enhanced confidence stratification. Our data show that 37–40% of sulfatides exhibit resolvable IFS peaks, underscoring the potential of this signal for sulfatide discrimination. This refinement would strengthen performance without compromising the algorithm's established effectiveness.<br><br>3.5 Comment 5<br>How did you recover the missing annotations? Please clarify/elabroate this<br><br>We appreciate this question regarding the recovery of the 14 and 19 sulfatide species that remained unannotated by Metaspace, even at permissive FDR thresholds of 50%. These missing annotations represent low-abundance sulfatide species for which only the monoisotopic $[M-H]^-$ peak exhibited sufficient signal intensity, while parts of the isotopic envelope, particularly the diagnostic ^{34}S isotopologues, fell below the instrument signal-to-noise threshold.<br>Manual spectral inspection at ultra-high mass resolving power (1,230,000 for the MR-MSI dataset) enabled identification of these species through their monoisotopic m/z values combined with pattern matching against the custom sulfatide database and validation against the external reference dataset (Gruber et al. 2025,

Powered by Editorial Manager® and ProduXion Manager® from Aries Systems Corporation

	https://www.nature.com/articles/s41467-025-59839-3). However, because the isotopic envelope could not be resolved for these low-abundance species, the Metaspace algorithm, which requires isotopic peak matching for confidence scoring, could not recognize them algorithmically. This represents a fundamental trade-off: whilst Metaspace requires visible isotopic evidence for high-confidence annotation, low-abundance species often lack sufficient signal intensity to resolve their full isotopic signature, even at ultra-high mass resolving power.<br>The recovery of these marginal cases highlights a critical sensitivity limitation in automated annotation workflows and demonstrates why expert curation informed by validated reference databases remains essential for comprehensive sulfatide characterization in ultra-high-resolution MSI datasets.<br><br><br>3.6 Final Comment<br>Would be happy to review after revisions.<br>We thank Reviewer 2 for the encouraging assessment and constructive feedback. We have systematically addressed all major concerns through comprehensive revision of the Discussion section, careful curation of the reference list, and clarification of annotation methodologies. We look forward to resubmission of the revised manuscript for further consideration.
**Additional Information:**	
**Question**	**Response**
Are you submitting this manuscript to a special series or article collection?	No
**Experimental design and statistics**<br><br><br>Full details of the experimental design and statistical methods used should be given in the Methods section, as detailed in our Minimum Standards Reporting Checklist. Information essential to interpreting the data presented should be made available in the figure legends.<br><br><br>Have you included all the information requested in your manuscript?	Yes
**Resources**<br><br><br>A description of all resources used, including antibodies, cell lines, animals and software tools, with enough information to allow them to be uniquely identified, should be included in the Methods section. Authors are strongly encouraged to cite Research Resource Identifiers (RRIDs) for antibodies, model organisms and tools, where possible.<br><br><br>Have you included the information	Yes

requested as detailed in our Minimum Standards Reporting Checklist?	
**Availability of data and materials**<br><br>All datasets and code on which the conclusions of the paper rely must be either included in your submission or deposited in publicly available repositories (where available and ethically appropriate), referencing such data using a unique identifier in the references and in the "Availability of Data and Materials" section of your manuscript.<br><br>Have you have met the above requirement as detailed in our Minimum Standards Reporting Checklist?	Yes
GigaScience has policies and guidelines in place for the use of generative AI-writing tools such as ChatGPT. If you have used such writing tools to assist with writing the manuscript this must be declared and cited in the text. Authors should not list AI-writing tools and other AI-assisted technologies as an author or co-author and should acknowledge that they are fully responsible for text generated or refined by AI-writing tools.<p><br><br>A summary of use (particularly in the introduction or among methods) needs to be included at the end of the paper, and the outputs should also be included as a supplementary file hosted in GigaDB or other open repositories. Please read our guidelines for more information. <p><br><br>By submitting to GigaScience, you are aware of the journal's AI-writing tools policy, and if you have declared use of such tools below, you have acknowledged this where appropriate in your manuscript and have made a summary of use and outputs available. <p><br><b>AI-assisted writing tools have been used in the preparation of this manuscript?	Yes

Clean revised manuscript without highlighted edits Click here to access/download;Manuscript;Gigascience_QCL-MRMS_Main_REV clean_final__new.docx

A sulfatide-centered ultra-high resolution magnetic resonance MALDI imaging benchmark dataset for MS1-based lipid annotation tools

Lars Gruber[1,2], Stefan Schmidt[1], Thomas Enzlein[1], Carsten Hopf[1,2,3,*]

[1]Center for Mass Spectrometry and Optical Spectroscopy (CeMOS), Technische Hochschule Mannheim, Paul-Wittsack-Str. 10, 68165 Mannheim

[2]Medical Faculty, Heidelberg University, Im Neuenheimer Feld 280, 69117 Heidelberg, Germany

[3]Mannheim Center for Translational Neuroscience (MCTN), Medical Faculty Mannheim, Heidelberg University, Theodor Kutzer-Ufer 1-3, 68167 Mannheim, Germany

*To whom correspondence should be addressed.

ABSTRACT: Spatial 'omics techniques are indispensable for studying complex biological systems and for the discovery of spatial biomarkers. While several current matrix-assisted laser desorption/ionization (MALDI) mass spectrometry imaging (MSI) instruments are capable of localizing numerous metabolites at high spatial and spectral resolution, the majority of MSI data is acquired at the MS1 level only. Assigning molecular identities based on MS1 data presents significant analytical and computational challenges, as the inherent limitations of MS1 data preclude confident annotations beyond the sum formula level. To enable future advancements of computational lipid annotation tools, well-characterized benchmark - or ground truth - datasets are crucial, which exceed the scope of synthetic data or data derived from mimetic tissue models. To this end, we provide two sulfatide-centered, biology-driven magnetic resonance MSI (MR-MSI) datasets at different mass resolving powers that characterize lipids in a mouse model of human metachromatic dystrophy. This data includes an ultra-high-resolution (R ~1,230,000) quantum cascade laser mid-infrared imaging-guided MR-MSI dataset that enables isotopic fine structure analysis and therefore enhances the level of confidence substantially. To highlight the usefulness of the data, we compared 118 manual sulfatide annotations with the number of decoy database-controlled sulfatide annotations performed in Metaspace (67 at FDR < 10%). Overall, our datasets can be used to benchmark annotation algorithms, validate spatial biomarker discovery pipelines, and serve as a reference for future studies that explore sulfatide metabolism and its spatial regulation.

Keywords: MALDI mass spectrometry, MRMS, MALDI imaging, mass spectrometry imaging, metabolite annotation tools, mid-infrared imaging, isotope fine structure, lipidomics, metabolomics

DATA DESCRIPTION

The absence of ground truth datasets, i.e., prior knowledge of which metabolites/lipid are present (or not) in a tissue of interest, and the non-availability of corresponding datasets containing high-confidence annotations for a large number of metabolites/lipids, has been posing a major obstacle to computational advancements in mass spectrometry imaging (MSI) [1]. In particular, datasets that can challenge computational tools for molecular annotation will be crucial for rapid progress in the field [2, 3]. To this end, we generated reusable and widely applicable datasets comprising quadruplicates of spatially focused, high-resolution mass spectrometry imaging data (MS1 level) derived from kidneys of an arylsulfatase A-deficient (ARSA-/-) mouse, a well-known genetic model of human metachromatic leukodystrophy [4]. Specifically, we developed a workflow that leverages Quantum Cascade Laser Mid-infrared (QCL-MIR) imaging to guide MSI on a 7T FT-ICR magnetic resonance mass spectrometer (MR-MS; **Supplementary Fig. 1 and 2**). The resulting MS1 data was interpreted in conjunction with precise reference annotations obtained for sulfatide glycosphingolipid species in defined kidney regions by on-tissue fragmentation-based lipid identification using imaging parallel reaction monitoring - parallel acquisition serial fragmentation (iprm-PASEF) on an orthogonal trapped ion mobility spectrometry (tims) TOF mass spectrometer [5]. Through combination of ultra-high resolution (R ~ 1,230,000) MR-MSI MS1 data with systematic MS2 data obtained on a different mass spectrometer, we are establishing a concept for generating such benchmark datasets. Four biological replicates and cross-modal validation against 4D-lipidomics TIMS-MS ensure data quality [5]. The ultra-high resolution dataset was further intended to be complemented by a high-resolution dataset. All files and preprocessing scripts are publicly available, thus supporting benchmarking and integration within spatial omics analyses, as further demonstrated in this work using Metaspace-ML [6].

CONTEXT

MALDI mass spectrometry imaging (MSI) has evolved into an invaluable tool in spatial biology [7–9] that enables the label-free detection and statistically validated visualization of molecular distributions in tissues [10, 11]. However, achieving reliable bimolecular interpretation of the inherently complex spatial molecular patterns fundamentally depends on the availability of high-quality datasets featuring unambiguous molecular identifications. Such datasets are crucial for facilitating the discovery of spatial biomarkers and yielding insights into tissue function, pathology, and pharmacodynamic or therapeutic responses [12, 13].

Prompted by the instrumental limitations outlined, for instance, in the 4S paradigm [7], several specialized methodologies for subspace imaging have been developed to facilitate the generation of high-quality mass spectrometry imaging (MSI) datasets, including spatial sparse sampling strategies [14, 15] or guided approaches. The latter comprises mass-guided approaches, e.g., single-cell imaging [16] or on-tissue MS2 [17] and imaging-guided approaches [18–22], including the recently developed QCL-MIR imaging-guided MSI workflow [5]. In general, these workflows have been introduced with the objective of enhancing overall throughput. Sometimes, acquisition time saved by restricting MSI to defined ROIs is reallocated to alternative workflows that operate MSI with advanced instrumental settings for MS1 data. These alternative workflows can include adjustments to laser beam settings [23], increased transient durations in FT-ICR MSI, or optimized ramp times in TIMS-MSI, which improve spatial resolution, mass resolution, and ion mobility separation, respectively. Furthermore, imaging-based guidance methods can be combined with a sophisticated MSI technique for on-tissue MS2 that utilizes ion mobility-enhanced methods such as iprm-PASEF [5, 17]. This integration substantially increased data quality by enhancing the confidence level for molecular identifications, all without the need for high-performance liquid chromatography (HPLC) separation and directly in the spatial context of the tissue [24]. Notably, the exploration of the chemical space of sulfatide isoforms in an ARSA-/- mouse model enabled us to introduce a ground truth to the MSI field, since sulfatides are known to accumulate in distinct ROIs of kidney sections from these mice.

As our QCL-MIR imaging-based guidance approach is inherently instrument-agnostic, we have applied it in this study to create an ultra-high-resolution, sulfatide-focused MS1 benchmark dataset using a 7T XR FT-ICR (**Supplementary Fig. 1 and 2**). Using externally validated annotations, this dataset may become a unique benchmark resource (**Fig. 1a**) for developing and enhancing MS1 tools for deep spatial lipidomics and related research fields. This is especially important, because most MSI studies today still rely on acquiring only MS1 data [9, 25].

By default, annotations of MS1 data are limited to the sum formula level. For this and other reasons, the unambiguous annotation of sum formulae to MS1 data remains a non-trivial task, even with ultra-high mass resolving power (R > 500.000) spectra, due to the sheer chemical diversity within biological samples [2, 26, 27]. Consequently, a single precise mass measurement may correspond to multiple candidate sum formulae, thereby complicating definitive assignment, even when the isotopic fine structures (IFS) can be resolved. Nevertheless, the progressive exploitation of accurate mass, isotopic envelope, and IFS information [26–29] substantially increases the reliability of metabolite annotation in MR-MSI-based spatial 'omics studies, a process in which computational tools play a crucial role by enabling automated annotation workflows. For future advancement of such tools in MSI, well-characterized benchmark datasets with high mass accuracy will be essential to enable robust validation and method development. This highlights the necessity and reuse potential of the dataset introduced in this study.

METHODS

Quantum-cascade laser mid-infrared (QCL-MIR) imaging of mouse kidneys

Animal studies involving ARSA-/- mice and cryo-sectioning of kidneys have been described before [5]. To ultimately focus ultra-high resolution MR-MSI data generation on defined kidney regions of interest (ROI) on an adjacent tissue section, we used QCL-MIR imaging for a pre-scan, followed by segmentation of hyperspectral QCL-MIR data to define ROIs [5]. These were then transferred to the MR-MSI instrument (**Supplementary Fig. 1 and 2**). To this end, QCL-MIR imaging data was recorded in sweep scan mode within a spectral range of 950–1800 cm^{-1} at a spectral sampling interval of 4 cm^{-1} on a Hyperion II ILIM (Bruker Optics, Ettlingen, Germany) equipped with a 3.5x objective. Subsequently, ROIs were generated and selected using *in-house* software [30] based on spatial sulfatide distributions in ARSA-/- mouse kidneys, which predominantly occur in the Inner Medulla/Papillae (IMP) and Inner Stripe of Outer Medulla (ISOM). Specifically, these ROIs were then targeted for MR-MSI data acquisition with transient times of 15.7s. All measurements were repeated for n=4 biological replicates.

Matrix spray-coating

10 mg/mL DHAP was dissolved in 70% ACN with 125 mM ammonium sulfate. After sonication, 0.1% TFA and 3 µM of SM4 35:1;O2 (100 µg/mL (= 157.41 µM) in MeOH/chloroform 2:1) as internal standard (IS) were added. Matrix was applied with an M5 TM-Sprayer (HTX Technologies, Chapel Hill, USA). Temperatures of the spray nozzle and tray were 75 °C and 35 °C, respectively. The spraying parameters were as follows: Spray Nozzle Velocity: 1200 mm/min; Flow Rate: 0.1 mL/min; No. of Passes: 10; Track Spacing: 2 mm; Pattern: HH; Pressure: 10 psi; Gas Low Rate: 2 L/min; Nozzle Height: 40 mm; Drying Time: 0s.

Magnetic resonance mass spectrometry imaging (MR-MSI) data acquisition

Ultra-high resolution MSI data was acquired on a solariX 7T XR Fourier Transform Ion Cyclotron Resonance (FT-ICR) MS (Bruker Daltonics, Bremen, Germany), equipped with a smartbeam II 2 kHz laser and ftms control 2.3.0 software (Bruker Daltonics, Build 92). Mass spectra were acquired in negative ion mode (*m/z* range 401.29–2600) and data acquisition size of 8 M, resulting in a free induction decay (FID) time of 15.7s according to and a mass resolving power of 1,230,000 at *m/z* 800. Reducing the number of data points in the time domain to 512k resulted in an FID time of 0.98 s and a mass resolving power of R~77,000. Ion optics settings were constant for all measurements: funnel RF amplitude (150 Vpp), source octopole (5 MHz, 350 Vpp), and collision cell voltage: 1.5 V, cell: 2 MHz, 1200 Vpp. The source DC optics were also constant for all measurements (capillary exit: -200 V, deflector plate: -220 V, funnel 1: -150 V, skimmer 1: -15 V), as well as the ParaCell parameters (transfer exit lens: 30 V, analyzer entrance: 10 V, sidekick: 0 V, side kick offset: 1.5 V, front/back trap plate: -3.4 V, back trap plate quench: 30 V). Sweet excitation power for ion detection was set to 14 %, and ion accumulation time was 0.05 s. The transfer optics were as follows: time of flight: 1 ms, frequency: 4 MHz, and RF amplitude: 350 Vpp. The laser parameters were laser power: 32 %, laser shots: 20, laser frequency: 200 Hz, and laser focus: medium, at a lateral step size of 40 µm.

Mass spectrometry imaging data analysis

For state-of-the-art annotation with Metaspace [31], the v2 (Metaspace ML [6]) algorithm and an *m/z* tolerance of 2 ppm were utilized. The imzML files were exported from SCiLS Lab (Version 2024a Pro, Bruker Daltonics). A custom reference database integrating LipidMaps with 780 theoretically derived sulfatide structures, available as **Supplementary Dataset 3**, was constructed for Metaspace analysis. All sulfatide species manually identified in this study (91 annotations from the first replicate, 97 from the second replicate) were established constituents of the original database prior to manual annotation, thereby ensuring methodological independence and precluding database incompleteness as a confounding factor in the assessment of algorithm performance. For manual annotation, we compared the QCL-MIR-guided MR-MSI data against the ground truth data from Gruber et al. [5]. For direct comparison of ultra-high- and high-resolution datasets, MS-based data segmentation was employed to enable subspace modeling. Bisecting k-means clustering of the MSI data from whole-tissue kidney sections was performed via SCiLS Lab to delineate anatomical regions, specifically the inner stripe of the outer medulla (ISOM) and the Inner Medulla/Papillae (IMP). These region outlines were subsequently used to generate a region-focused dataset, facilitating a more targeted comparative analysis.

MATERIALS

All chemicals and solvents were of HPLC-MS grade. Conductive indium tin oxide (ITO)-coated glass slides were purchased from Diamond Coatings (West Midlands, UK). The MALDI matrix 2,5-dihydroxyacetophenone (DHAP) was purchased from Thermo Fisher Scientific (Waltham, Massachusetts, USA). Acetonitrile (ACN), ethanol (EtOH), LC-MS water, 2-propanol (IPA), and ammonium sulfate (AmS) were obtained from VWR Chemicals (Darmstadt, Germany). The sulfatide standard C_{17} mono-sulfo galactosyl(β) ceramide d18:1/17:0 (SM4 35:1;O2) was purchased from Avanti Polar Lipids (Birmingham, USA). Trifluoroacetic acid (TFA), Mayer's hemalum solution, hydrochloric acid, sodium bicarbonate, magnesium sulfate, eosin Y-solution 0.5%, xylene, and eukitt were purchased from Merck KGaA.

DATA VALIDATION AND QUALITY CONTROL

To enable the advancement of MS^1-based molecular annotation tools for spatial biomarker discovery pipelines and other purposes, we acquired and in-depth-characterized two sulfatide-centered, biology-driven datasets derived from an arylsulfatase A mouse model at two different mass resolving powers. This includes an ultra-high mass resolution MR-MSI dataset (R ~ 1,230,000) acquired using our recently introduced Quantum Cascade Laser (QCL) MIR guidance approach [5], in this study, combined with a 7T FT-ICR mass spectrometer. Briefly summarized, this workflow utilizes QCL-MIR imaging microscopy to rapidly acquire hyperspectral data from tissue samples, here, fresh-frozen kidney sections. Subsequent application of unsupervised segmentation algorithms, specifically k-means clustering, allows for the identification of biologically relevant regions of interest (ROIs) within the kidney tissue, particularly inner medulla/papilla (IMP) and inner stripe of outer medulla (ISOM), where sulfatides are enriched in ARSA -/- mice. Ultra-high-resolution MR-MSI was then performed in a ROI-targeted manner on these two tissue morphologies within kidneys from two 12-week-old and two 60-week-old ARSA-/- mice (**Fig. 1a; Supplementary Fig. 1** and **2**). The QCL-MIR imaging enabled focus on just two morphologies, enhancing both analytical depth and data acquisition efficiency, e.g., measurement time. As a reference and benchmark for the molecular content of these tissue areas and the total number of potential sulfatide identifications, we relied on our published reference data, which is based on three pillars: a known biological pathway leading to lipid-class specific accumulation of sulfatides, iprm-PASEF-derived molecular identifications in conjunction with 4D lipidomics LC-MS data (Supplementary Table 1), and systematic MS/MS fragmentation validation [5]. Sulfatide structural identifications were systematically validated through on-tissue parallel reaction monitoring (PRM) with ion mobility separation. Diagnostic fragment ions specific to each sulfatide subclass provide an unambiguous biochemical chain of evidence. These include neutral loss of the α-hydroxylated fatty acid (α-OH-FA), fragment for a phytosphingoid backbone (PSPB-Gal-SO_3^-), as well as characteristic sulfate-containing headgroup fragments: GalNAc-Gal-SO_3^-, Glc-Gal-SO_3^-, and RCF-Gal-SO_3^- for the respective sulfatide species (SM4, SM3, SM2a, and SB1a). Common fragments, such as Gal-SO_3^- and HSO_4^-, appear across all sulfatide

subclasses and serve as internal validation markers. Representative MS/MS spectra from sulfatide standards demonstrated high consistency (cosine similarity ≥ 0.99) across replicates, confirming the reliability and reproducibility of these fragmentation patterns. All manual annotations in this benchmark dataset were assigned only after successful MS/MS validation within the reference dataset, ensuring maximal confidence for downstream benchmarking studies.

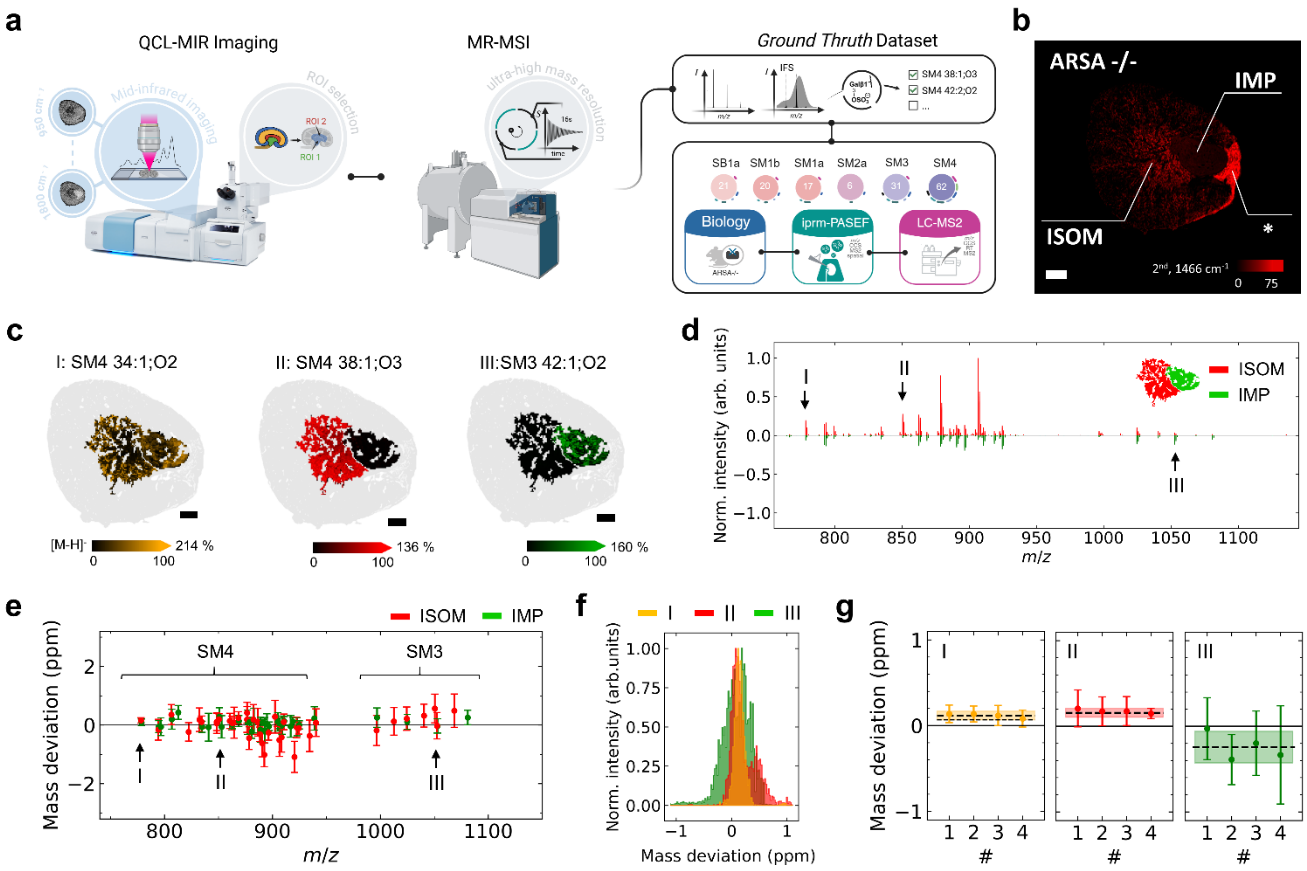

Figure 1. A sulfatide-centered, ultra-high resolution QCL-MIR guided MSI benchmark dataset.
a, Schematic overview of data acquisition and incorporation of reference information. The methodology includes quantum cascade laser mid-infrared (QCL-MIR) imaging of kidney sections for region-focused magnetic resonance mass spectrometry imaging (MR-MSI) at ultra-long transient times (~ 16s), followed by manual sulfatide annotations based on a reference dataset validated at MS2 level [5]. **b,** Representative lipid-distribution in an ARSA-/- kidney section based on the 2nd derivative of absorbance (2nd) at 1466 cm^{-1} yielded predominant sulfatide accumulation in the ISOM region. The asterisk marks a region of high lipid content as described in [5] . Scale bar, 500 µm. **c,** Overlay of region-focused ion images of **(I)** *m/z* 778.5146 (SM4 34:1;O2[M-H]$^-$; orange), **(II)** *m/z* 850.5721 (SM4 38:1;O3[M-H]$^-$; red), and **(III)** *m/z* 1052.6923 (SM3 42:1;O2[M-H]$^-$; green) in kidney (grey). Mass window, ±3 ppm. **d,** Representative butterfly plot of average mass spectra for the inner medulla/papilla (IMP; green) and ISOM (red) identified by QCL-MIR. **e,** Mass deviation and uncertainties (standard deviation) for 47 sulfatides (signals present in at least 50 pixels of either region IMP or ISOM) at R_2~1.23 M. *m/z* values are shifted by +0.2 (IMP) or -0.2 (ISOM) for visualization. **f,** Histogram of sum intensities in ISOM and IMP for (I), (II), and (III) measured with a mass resolution of ~1.23M at *m/z* 800. **g,** Weighted mean mass deviation (dotted line) and uncertainty (*n*=4) presented as internal error[1] (filled area) for (I), (II), and (III). To this end, following Roux et al. [32] the external and internal errors were calculated, with the greater of the two selected as the final uncertainty to provide a conservative estimate. The shown results are consistent with the theoretical value within two standard deviations.

We compared conventional MR-MSI of whole kidney slices at a mass resolution of R_1~77,000 at m/z 800 (1s free induction decay (FID) time; 14,331 pixels, 40 x 40 µm^2 pixel size; 5 hours of data acquisition) in the FT-ICR with QCL-MIR-guided analysis (**Fig. 1b**) focused on the ISOM and IMP ROIs, which achieved R_2~1,230,000 at *m/z* 800 (16s FID time; 2,672 pixels, 40 x 40 µm^2 pixel size; 11.6 hours of data acquisition), resulting in a 16-fold increase in mass resolving power (**Supplementary Fig. 3**). For the QCL-MIR-guided MR-MSI dataset, three sulfatide ion images are presented as examples that displayed similar intensities in both ROIs (**I**, *m/z* 778.5146 (SM4 34:1;O2[M-H]$^-$)), higher intensity in ISOM (**II**, *m/z* 850.5721 (SM4 38:1;O3[M-H]$^-$)), or higher intensity in IMP (**III**, m/z 1052.6923 [SM3 42:1;O2[M-H]-). Unique molecular fingerprints were obtained per region (**Fig. 1d**). In both datasets, the mass deviation was constant across the *m/z* range (750-1100) and was consistently below 2 ppm, even when considering the uncertainties across n=4 biological replicates (**Fig. 2e; Supplementary Fig. 4**). The maximum mass deviation was about 1 ppm for the two less intense sulfatide ions **(I)** and **(III)**

and less than 0.2 ppm for ion **(II)** at R_1~77,000, improving to below 0.2 ppm for **(I)** and (**II)** (around 0.5 ppm for (**III)**) with a mass resolution of R_2~1,230,000 (**Fig. 1f; Supplementary Fig. 4**). The reproducibility of our data is emphasized by comparing the mass deviation across n=4 biological replicates for ions (**I)-(III)**, all of which showed values below 1 ppm (**Fig. 1g**). Overall, the uncertainty of *m/z* values was reduced by a factor of 3-6 for data acquired at ultra-high resolution on an FT-ICR instrument.

The ultra-high mass resolving power applied to the QCL-MIR dataset enables the detection of isotopic fine structures (IFS) for the sulfatides. The IFS, particularly the peak attributed to ^{34}S, was very well resolved, with a signal-to-noise ratio of approximately 10 times the FWHM (**Fig. 2a; Supplementary Fig. 5; Supplementary Dataset 2**). Nevertheless, it is important to note that in all cases where FID times are notably prolonged, it is necessary to operate with a reduced total ion current. This precautionary measure is pivotal to reduce (local) space charge effects [33–35] within the ion cyclotron resonance (ICR) cell (**Supplementary Fig. 6**). However, this results in a loss of sensitivity, which in turn leads to slightly reduced numbers of sulfatide annotations, in particular for those isoforms that have a comparatively lower concentration in IMP and ISOM than in the cortex. Overall, the number of candidate sulfatide identifications using the QCL-MIR imaging-guided ultra-high-mass resolution MR-MSI method was 91 and 97 for the two 60-week ARSA-/- mice, compared to 118 and 115 annotations obtained with the conventional whole-tissue method (**Table 1**). The identification confidence was, on the other hand, dramatically improved, as 34 and 39 ultra-high-mass resolution spectra were supported by IFS information (**Table 1**). Identification was performed manually, as IFS is currently not utilized adequately in commercial sum formula annotation tools, which match experimental against theoretical isotope patterns [36]. In the sulfatide case, the ^{34}S isotope peak was not automatically recognized as such, but pre-definition of S and N as constituents of the molecule-of-interest in the Bruker Smart Formula tool led to successful searches.

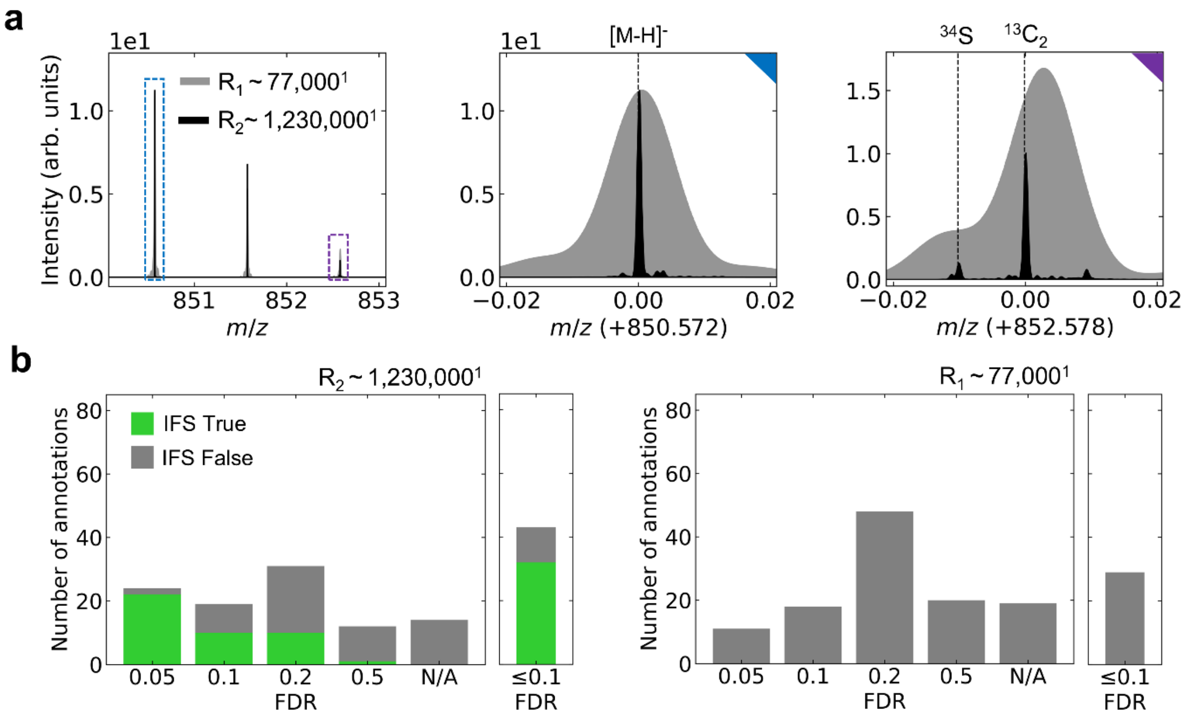

Figure 2. Evaluation of the annotation quality for the sulfatide-centered QCL-MIR guided MSI dataset.
a, Ultra-high resolution MR-MSI data was acquired with a mass resolution of R_1~77,000 (gray) and R_2~1,230,000 (black). Isotopic fine structure (IFS) of SM4 38:1;O3[M-H]⁻ incl. $^{13}C_2$ (M+2) and ^{34}S isotopic peaks, normalized to the monoisotopic peak. **b,** Sulfatide annotations were performed using Metaspace with an in-house database containing 780 theoretical sulfatides compiled from LipidMaps. The ultra-high-resolution dataset (R_2 ~ 1,230,000, left) yielded substantially higher annotation rates than the standard resolution dataset (R_1 ~ 77,000, right), demonstrating the superior discriminatory power of ultra-high mass resolving power for sulfatide characterization. At FDR ≤ 10%, the ultra-high-resolution dataset identified 43 total sulfatides (32 with IFS confirmation, 11 without), compared to only 29 annotations at the same FDR threshold for the standard resolution dataset (which yielded 11 annotations at the more stringent FDR ≤ 5%). [1]Mass resolving power at *m/z* 800. N/A marks annotations that were performed manually (and validated via *in-situ* MS/MS and/or LC-MS/MS) but were not annotated by Metaspace at any FDR level.

To highlight the potential reuse of our dataset as a benchmark for cutting-edge MALDI MSI data annotation, we employed the open-source Metaspace platform [37] to compare sulfatide annotations identified across varying false-discovery rate (FDR) thresholds. FDR-based quality measures have recently been critically assessed in the more mature field of proteomics [38], as i) FDR determination is often a black box, and ii) it is often unclear at what level FDR is set. For MSI, the FDR-control process

in Metaspace is fairly transparent [31]. Given the limited coverage of sulfatides in publicly available databases, we curated a custom database integrating LipidMaps entries with 780 theoretically derived sulfatide structures (**Supplementary Dataset 3**). For data acquired at 77,000 mass resolving power, IMP and ISOM were extracted using MS feature-based segmentation to maintain consistency in the analysis.

Comparison of our manual annotations with Metaspace's database-controlled annotations at different FDR thresholds reveals both the effectiveness of current annotation algorithms and specific limitations when applied to ultra-high-resolution FT-ICR MSI datasets. The superior performance of ultra-high mass resolving power is evident across all FDR stringency levels. At FDR ≤ 5%, the ultra-high-resolution dataset (1,230,000 R) yielded 24 sulfatide annotations (22 supported by IFS), whereas the standard resolution dataset (77,000 R) identified only 11 annotations (**Fig. 2b**). At a 10% FDR threshold, this performance gap widened substantially: Metaspace identified 43 sulfatides from the ultra-high-resolution dataset (32 confirmed by IFS peaks), compared to only 29 annotations at standard resolution, demonstrating robust algorithmic performance and the critical importance of mass resolving power for sulfatide characterization. The number of annotations continued to increase with more permissive FDR thresholds, reaching 74 sulfatides (42 supported by IFS) at FDR ≥ 20% for the ultra-high-resolution dataset and 77 for the standard resolution dataset, though the relative proportion supported by IFS declined with increasing FDR stringency.

However, 14 sulfatides remained unannotated (N/A in **Fig. 2b**) even at a permissive ≥50% FDR threshold. These unannotated species represent low-abundance compounds for which only the monoisotopic $[M-H]^-$ peak exceeded the signal-to-noise threshold, while the complete isotopic envelope fell below detection limits (**Supplementary Fig. 7**). This represents a measurement constraint rather than an algorithmic deficiency: Metaspace appropriately requires resolvable isotopic envelope information for confident annotation and conservatively rejects *m/z* features with incomplete or missing isotopic patterns rather than generating spurious assignments. These missing annotations were recovered through manual monoisotopic *m/z* matching against the custom reference database and external validation [5].

Metaspace's scoring method limits isotopic envelope matching to the four most intense peaks. While this approach successfully annotates species with sufficient abundance to resolve their complete isotopic patterns, it fails to exploit diagnostic IFS information when present above noise thresholds. Our analysis demonstrates that 37% of sulfatide identifications in the first replicate (34 of 91) and 40% in the second replicate (39 of 97) were confirmed by detecting ^{34}S IFS peaks. This represents an underutilized signal: high-confidence annotations substantiated by robust IFS peak detection remain indistinguishable from lower-confidence assignments based solely on precursor *m/z* and mass error tolerance within the current algorithmic framework.

Future annotation algorithms should be refined to leverage the full isotopic envelope in ultra-high-resolution datasets while explicitly accounting for signal-intensity-dependent constraints on IFS detectability. Prospective algorithmic developments should incorporate: 1) expansion of isotopic envelope matching to encompass all resolved peaks above instrument-specific signal-to-noise thresholds, superseding the current four-peak limitation; 2) implementation of confidence stratification metrics that explicitly correlate annotation reliability with precursor ion signal intensity and noise levels, acknowledging that minor isotopic constituents—such as ^{34}S with 4.5% relative abundance compared to the monoisotopic form (exemplified by SM4 38:1;O3 in **Fig. 2a**)—exhibit attenuated detectability at reduced precursor ion intensities, thereby imposing practical limitations on IFS-based structural discrimination for low-abundance species; 3) integration of machine-learning classification frameworks trained on empirically acquired IFS signatures from characterized standards, enabling recognition of subclass-specific isotopic patterns across heterogeneous signal intensity regimes; and 4) incorporation of complementary analytical dimensions such as TIMS-FT-ICR (recently applied to lipid analysis) or MS/MS fragmentation patterns to adjudicate between isotopically indistinguishable molecular formulas when mass defect alone provides insufficient discriminatory power [39, 40]. While such hyphenated approaches show promise for structural discrimination, their integration into spatial lipidomics workflows remains under development [39, 40]. This benchmark dataset provides a validated ground truth required for development and rigorous evaluation of such algorithmic advances, establishing quantitative performance benchmarks for annotation tools in the ultra-high-resolution regime and reinforcing that expert curation remains indispensable despite high data quality achieved with contemporary instrumentation.

RE-USE POTENTIAL

This rigorously validated (see [5]) sulfatide-focused benchmark dataset offers significant reuse potential for the MSI community. Collected at two levels of mass resolving power, the dataset enables a thorough evaluation of annotation strategies, especially those that utilize isotopic fine structure (IFS) for high-confidence MS1-level metabolite identification. As shown, IFS analysis yields greater annotation accuracy than current automated platforms, highlighting the need for further methodological innovation in computational annotation. Although some studies describe ultra-high resolution [41, 42], none of these share their data under FAIR principles, and no externally validated annotations are available. Notably, the ultra-high-resolution QCL-MIR-guided approach used here emphasizes annotation confidence through isotopic fine structure analysis and reduced ion current to minimize space-charge effects; however, this optimization involves trade-offs in sensitivity and

spatial coverage compared to traditional whole-tissue MSI experiments. The number of sulfatides identified in the QCL-MIR-guided regions of interest (91 and 97 for the two replicates) was lower than in the corresponding unguided whole-tissue analysis (118 and 115), reflecting this methodological trade-off between analytical depth and comprehensive tissue coverage. This dataset is a valuable resource for benchmarking and validating spatial biomarker discovery pipelines, developing new annotation algorithms, and supporting future research into sulfatide metabolism and its spatial regulation in biological tissues. Its utility for both computational tool development and biological studies ensures ongoing relevance for advancing spatial metabolomics and related fields.

DATA AVAILABILITY

The underlying MALDI MR-MSI raw data supporting the findings of this study are openly available in Zenodo at http://doi.org/10.5281/zenodo.16842680. The *.imzML* files of the processed MR-MSI are available via Metaspace under https://metaspace2020.org/api_auth/review?prj=0e7b6e78-78cf-11f0-a049-172853cb2b10&token=EaH8S_2gCKoX.

CODE AVAILABILITY

Data acquisition was conducted using existing tools (e,g, https://github.com/CeMOS-Mannheim/QCL_MIR_guided_MSI) and methods as described in the Methods section.

DECLARATIONS

List of abbreviations

ARSA	Arylsulfatase A
FDR	False discovery rate
FID	Free induction decay
FT-ICR	Fourier transform ion cyclotron resonance
FWHM	Full width at half maximum
IFS	Isotopic fine structure
IMP	Inner medulla/papillae
ISOM	Inner stripe of outer medulla
LC-MS	Liquid chromatography mass spectrometry
MALDI	Matrix-assisted laser desorption/ionization
MIR	Mid-infrared
MRMS	Magnetic resonance mass spectrometry
MSI	Mass spectrometry imaging
m/z	mass to charge ratio
QCL	Quantum cascade laser
ROI	Region of interest

Author Contributions (CRediT)
Lars Gruber: Methodology, Investigation, Formal Analysis, Writing - Original Draft
Stefan Schmidt: Methodology, Investigation, Formal Analysis, Writing - Original Draft
Thomas Enzlein: Formal Analysis, Visualization
Carsten Hopf: Conceptualization, Supervision, Project Administration, Resources, Writing - Review & Editing

Funding (FundRef)
This work was supported by:
- The Bundesministerium für Bildung und Forschung (BMBF) under grants 12FH8I05IA (Drugs4Future) and 13FH8I09IA (DrugsData) within the M2Aind partnership (to Carsten Hopf);
- The Ministerium für Wissenschaft, Forschung und Kunst Baden-Württemberg (MWK) via the Mittelbauprogramm (to Carsten Hopf);
- The Deutsche Forschungsgemeinschaft (DFG, project 262133997) for the acquisition of the solariX 7T XR (to Carsten Hopf) and the grant INST874/9-1 (project 497984836).

The funders had no role in study design, data collection/analysis, interpretation, or manuscript preparation.

Competing Interests
Bruker Daltonics co-funded the BMBF-funded projects “Drugs4Future” and “DrugsData” within the framework M^{2}Aind, as mandated by BMBF, but did not influence this study. All other authors declare no competing interests.

DECLARATION OF GENERATIVE AI AND AI-ASSISTED TECHNOLOGIES IN THE WRITING PROCESS

During the preparation of this work, the author(s) used perplexity.ai to improve the readability and language of the manuscript. After using this tool/service, the author(s) reviewed and edited the content as needed and take(s) full responsibility for the content of the published article.

ASSOCIATED CONTENT

Supporting Information

The Supporting Information is available free of charge.

AUTHOR INFORMATION

Corresponding Author

*Carsten Hopf, c.hopf@th-mannheim.de, Center for Mass Spectrometry and Optical Spectroscopy (CeMOS), Technische Hochschule Mannheim, Paul-Wittsack-Str. 10, 68165 Mannheim

Table 1: Cumulative numbers of sulfatide subclass isoforms identified in ARSA-/- mouse kidney by QCL-MIR imaging-guided MR-MSI.
Whole kidney sections of 12- or 60-week-old ARSA-/- mice (n=2 each) were analyzed by conventional non-guided MR-MSI with a mass resolution of R_1 ~ 77k (at m/z 800) and QCL-MIR imaging-guided MR-MSI with a mass resolution of R_2 ~ 1,230k (at *m/z* 800). In many cases of QCL-MIR imaging-guided MSI, isotope fine structures (IFS) could be used for added confidence.

			SM4	SM3	SM2a	SB1a	total
**R_1, FID 1s**	Total annotations in ROI	60w_1	56	37	6	19	118
		60w_2	54	36	6	19	115
		12w_1	45	36	6	16	103
		12w_2	52	36	6	18	112
**R_2, FID 16s**	Total annotations in ROI	60w_1	54	25	0	12	91
		60w_2	54	27	4	12	97
		12w_1	42	16	1	8	67
		12w_2	52	19	1	5	77
	Annotations with Confirmed IFS Evidence	60w_1	25	7	0	2	34
		60w_2	27	8	0	4	39
		12w_1	17	4	0	1	22
		12w_2	20	6	0	0	26

References

1. Alexandrov T. Spatial Metabolomics and Imaging Mass Spectrometry in the Age of Artificial Intelligence. Annu Rev Biomed Data Sci. 2020;3:61–87. doi:10.1146/annurev-biodatasci-011420-031537.

2. Baquer G, Sementé L, Mahamdi T, Correig X, Ràfols P, García-Altares M. What are we imaging? Software tools and experimental strategies for annotation and identification of small molecules in mass spectrometry imaging. Mass Spectrom Rev. 2023;42:1927–64. doi:10.1002/mas.21794.

3. Cochran D, Powers R. Fourier Transform Ion Cyclotron Resonance Mass Spectrometry Applications for Metabolomics. Biomedicines 2024. doi:10.3390/biomedicines12081786.

4. Hess B, Saftig P, Hartmann D, Coenen R, Lüllmann-Rauch R, Goebel HH, et al. Phenotype of arylsulfatase A-deficient mice: relationship to human metachromatic leukodystrophy. Proc Natl Acad Sci U S A. 1996;93:14821–6. doi:10.1073/pnas.93.25.14821.

5. Gruber L, Schmidt S, Enzlein T, Vo HG, Bausbacher T, Cairns JL, et al. Deep MALDI-MS spatial omics guided by quantum cascade laser mid-infrared imaging microscopy. Nat Commun. 2025;16:4759. doi:10.1038/s41467-025-59839-3.

6. Wadie B, Stuart L, Rath CM, Drotleff B, Mamedov S, Alexandrov T. METASPACE-ML: Context-specific metabolite annotation for imaging mass spectrometry using machine learning. Nat Commun. 2024;15:9110. doi:10.1038/s41467-024-52213-9.

7. Schulz S, Becker M, Groseclose MR, Schadt S, Hopf C. Advanced MALDI mass spectrometry imaging in pharmaceutical research and drug development. Curr Opin Biotechnol. 2019;55:51–9. doi:10.1016/j.copbio.2018.08.003.

8. Ma X, Fernández FM. Advances in mass spectrometry imaging for spatial cancer metabolomics. Mass Spectrom Rev. 2024;43:235–68. doi:10.1002/mas.21804.

9. Ngai YT, Lau D, Mittal P, Hoffmann P. Mini Review: Highlight of Recent Advances and Applications of MALDI Mass Spectrometry Imaging in 2024. Anal Sci Adv. 2025;6:e70016. doi:10.1002/ansa.70016.

10. Abu Sammour D, Cairns JL, Boskamp T, Marsching C, Kessler T, Ramallo Guevara C, et al. Spatial probabilistic mapping of metabolite ensembles in mass spectrometry imaging. Nat Commun. 2023;14:1823. doi:10.1038/s41467-023-37394-z.

11. Spangenberg P, Bessler S, Widera L, Bottek J, Richter M, Thiebes S, et al. msiFlow: automated workflows for reproducible and scalable multimodal mass spectrometry imaging and microscopy data analysis. Nat Commun. 2025;16:1065. doi:10.1038/s41467-024-55306-7.

12. Zhang H, Lu KH, Ebbini M, Huang P, Lu H, Li L. Mass spectrometry imaging for spatially resolved multi-omics molecular mapping. Npj Imaging. 2024;2:20. doi:10.1038/s44303-024-00025-3.

13. Rosenberger FA, Thielert M, Mann M. Making single-cell proteomics biologically relevant. Nat Methods. 2023;20:320–3. doi:10.1038/s41592-023-01771-9.

14. Hu H, Helminiak D, Yang M, Unsihuay D, Hilger RT, Ye DH, Laskin J. High-Throughput Mass Spectrometry Imaging with Dynamic Sparse Sampling. ACS Meas Sci Au. 2022;2:466–74. doi:10.1021/acsmeasuresciau.2c00031.

15. Xie YR, Castro DC, Rubakhin SS, Sweedler JV, Lam F. Enhancing the Throughput of FT Mass Spectrometry Imaging Using Joint Compressed Sensing and Subspace Modeling. Anal Chem. 2022;94:5335–43. doi:10.1021/acs.analchem.1c05279.

16. Cairns JL, Huber J, Lewen A, Jung J, Maurer SJ, Bausbacher T, et al. Mass-Guided Single-Cell MALDI Imaging of Low-Mass Metabolites Reveals Cellular Activation Markers. Adv Sci (Weinh). 2025;12:e2410506. doi:10.1002/advs.202410506.

17. Heuckeroth S, Behrens A, Wolf C, Fütterer A, Nordhorn ID, Kronenberg K, et al. On-tissue dataset-dependent MALDI-TIMS-MS2 bioimaging. Nat Commun. 2023;14:7495. doi:10.1038/s41467-023-43298-9.

18. Patterson NH, Tuck M, van de Plas R, Caprioli RM. Advanced Registration and Analysis of MALDI Imaging Mass Spectrometry Measurements through Autofluorescence Microscopy. Anal Chem. 2018;90:12395–403. doi:10.1021/acs.analchem.8b02884.

19. Rabe J-H, A Sammour D, Schulz S, Munteanu B, Ott M, Ochs K, et al. Fourier Transform Infrared Microscopy Enables Guidance of Automated Mass Spectrometry Imaging to Predefined Tissue Morphologies. Sci Rep. 2018;8:313. doi:10.1038/s41598-017-18477-6.

20. Blutke A, Sun N, Xu Z, Buck A, Harrison L, Schriever SC, et al. Light sheet fluorescence microscopy guided MALDI-imaging mass spectrometry of cleared tissue samples. Sci Rep. 2020;10:14461. doi:10.1038/s41598-020-71465-1.

21. Choe K, Xue P, Zhao H, Sweedler JV. macroMS: Image-Guided Analysis of Random Objects by Matrix-Assisted Laser Desorption/Ionization Time-of-Flight Mass Spectrometry. J Am Soc Mass Spectrom. 2021;32:1180–8. doi:10.1021/jasms.1c00013.

22. Esselman AB, Patterson NH, Migas LG, Dufresne M, Djambazova KV, Colley ME, et al. Microscopy-Directed Imaging Mass Spectrometry for Rapid High Spatial Resolution Molecular Imaging of Glomeruli. J Am Soc Mass Spectrom. 2023;34:1305–14. doi:10.1021/jasms.3c00033.

23. Croslow SW, Trinklein TJ, Sweedler JV. Advances in multimodal mass spectrometry for single-cell analysis and imaging enhancement. FEBS Lett. 2024;598:591–601. doi:10.1002/1873-3468.14798.

24. Gachumi G, Purves RW, Hopf C, El-Aneed A. Fast Quantification Without Conventional Chromatography, The Growing Power of Mass Spectrometry. Anal Chem. 2020;92:8628–37. doi:10.1021/acs.analchem.0c00877.

25. Yuan J, Li X, Shen X, Xiong P, Zhu N, Ye Y, Liu J. Comprehensive Metabolite Profiling in Single-Cell Systems via Dual-Modal MALDI-Mass Spectrometry Imaging. Anal Chem. 2025;97:8729–37. doi:10.1021/acs.analchem.4c05480.

26. Shi SD, Hendrickson CL, Marshall AG. Counting individual sulfur atoms in a protein by ultrahigh-resolution Fourier transform ion cyclotron resonance mass spectrometry: experimental resolution of isotopic fine structure in proteins. Proc Natl Acad Sci U S A. 1998;95:11532–7. doi:10.1073/pnas.95.20.11532.

27. Tiquet M, La Rocca R, Kirnbauer S, Zoratto S, van Kruining D, Quinton L, et al. FT-ICR Mass Spectrometry Imaging at Extreme Mass Resolving Power Using a Dynamically Harmonized ICR Cell with 1ω or 2ω Detection. Anal Chem. 2022;94:9316–26. doi:10.1021/acs.analchem.2c00754.

28. Popov IA, Nagornov K, Vladimirov GN, Kostyukevich YI, Nikolaev EN. Twelve million resolving power on 4.7 T Fourier transform ion cyclotron resonance instrument with dynamically harmonized cell--observation of fine structure in peptide mass spectra. J Am Soc Mass Spectrom. 2014;25:790–9. doi:10.1007/s13361-014-0846-7.

29. Sun Z, Wang F, Liu Y, Deng B, Ren R, Wang Y, et al. Recent strategies for improving MALDI mass spectrometry imaging performance towards low molecular weight compounds. TrAC Trends in Analytical Chemistry. 2024;175:117727. doi:10.1016/j.trac.2024.117727.

30. Schmidt S. in-house software for ROI transfer. https://github.com/CeMOS-Mannheim/QCL_MIR_guided_MSI.

31. Palmer A, Phapale P, Chernyavsky I, Lavigne R, Fay D, Tarasov A, et al. FDR-controlled metabolite annotation for high-resolution imaging mass spectrometry. Nat Methods. 2017;14:57–60. doi:10.1038/nmeth.4072.

32. Roux C, Blaum K, Block M, Droese C, Eliseev S, Goncharov M, et al. Data analysis of Q-value measurements for double-electron capture with SHIPTRAP. Eur. Phys. J. D 2013. doi:10.1140/epjd/e2013-40110-x.

33. Hohenester UM, Barbier Saint-Hilaire P, Fenaille F, Cole RB. Investigation of space charge effects and ion trapping capacity on direct introduction ultra-high-resolution mass spectrometry workflows for metabolomics. J Mass Spectrom. 2020;55:e4613. doi:10.1002/jms.4613.

34. Pieczonka SA, Thomas MJ, Schmitt-Kopplin P, Marshall JW. Harmonization of FT-ICR-MS Instruments for Interoperable Multi-Laboratory Comprehensive Compositional Profiling. Anal Chem. 2025;97:8491–8. doi:10.1021/acs.analchem.5c00488.

35. Wong RL, Amster IJ. Experimental Evidence for Space-Charge Effects between Ions of the Same Mass-to-Charge in Fourier-Transform Ion Cyclotron Resonance Mass Spectrometry. Int J Mass Spectrom. 2007;265:99–105. doi:10.1016/j.ijms.2007.01.014.

36. Thompson CJ, Witt M, Forcisi S, Moritz F, Kessler N, Laukien FH, Schmitt-Kopplin P. An Enhanced Isotopic Fine Structure Method for Exact Mass Analysis in Discovery Metabolomics: FIA-CASI-FTMS. J Am Soc Mass Spectrom. 2020;31:2025–34. doi:10.1021/jasms.0c00047.

37. Alexandrov T. METASPACE annotation platform. http://www.metaspace2020.eu.

38. The M, Samaras P, Kuster B, Wilhelm M. Reanalysis of ProteomicsDB Using an Accurate, Sensitive, and Scalable False Discovery Rate Estimation Approach for Protein Groups. Mol Cell Proteomics. 2022;21:100437. doi:10.1016/j.mcpro.2022.100437.

39. Benigni P, Porter J, Ridgeway ME, Park MA, Fernandez-Lima F. Increasing Analytical Separation and Duty Cycle with Nonlinear Analytical Mobility Scan Functions in TIMS-FT-ICR MS. Anal Chem. 2018;90:2446–50. doi:10.1021/acs.analchem.7b04053.

40. Wootton CA, Maillard J, Theisen A, Brabeck GF, Schat CL, Rüger CP, et al. A Gated TIMS FTICR MS Instrument to Decipher Isomeric Content of Complex Organic Mixtures. Anal Chem. 2024;96:11343–52. doi:10.1021/acs.analchem.4c01370.

41. Vandergrift GW, Zemaitis KJ, Veličković D, Lukowski JK, Paša-Tolić L, Anderton CR, Kew W. Experimental Assessment of Mammalian Lipidome Complexity Using Multimodal 21 T FTICR Mass Spectrometry Imaging. Anal Chem. 2023;95:10921–9. doi:10.1021/acs.analchem.3c00518.

42. Grgic A, Nagornov KO, Kozhinov AN, Michael JA, Anthony IGM, Tsybin YO, et al. Ultrahigh-Mass Resolution Mass Spectrometry Imaging with an Orbitrap Externally Coupled to a High-Performance Data Acquisition System. Anal Chem. 2024;96:794–801. doi:10.1021/acs.analchem.3c04146.

 Click here to access/download;Figure;Fig2 new.png

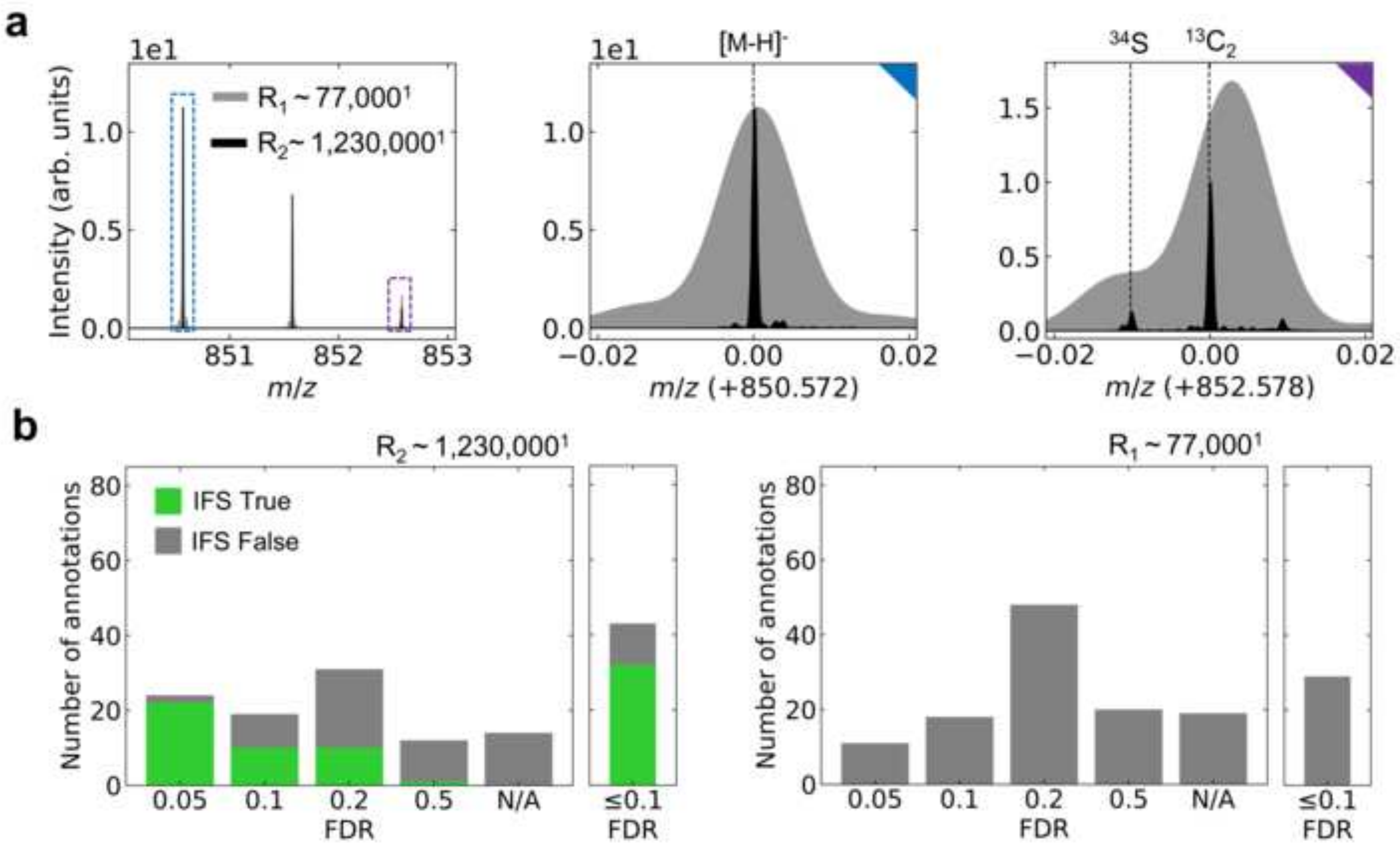

revised Figure 1

Click here to access/download;Figure;Fig1 new.png

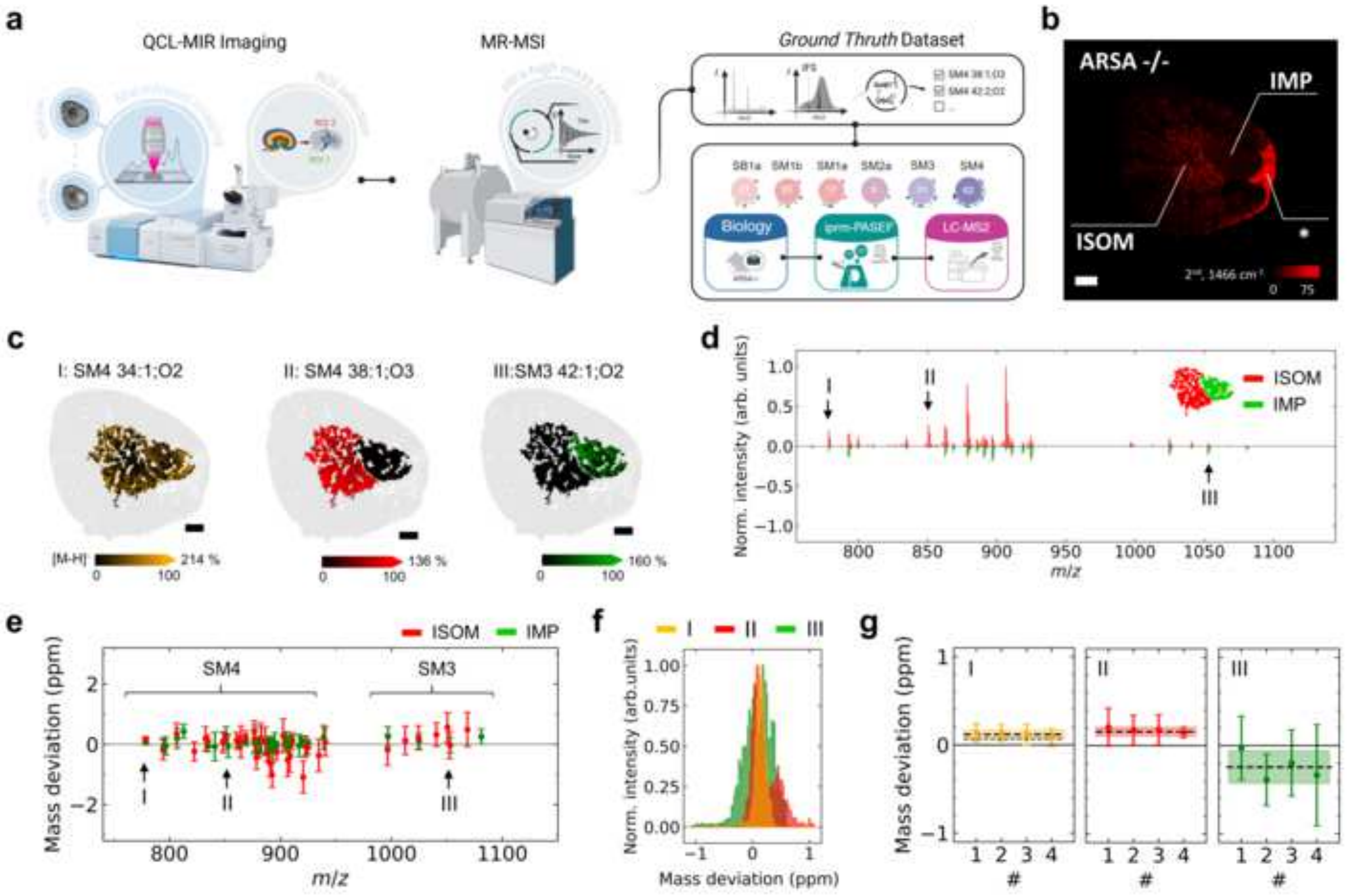

Supplementary Dataset 1

Click here to access/download
Supplementary Material
Supplementary_Dataset_1_FTICR_1s.rar

Supplementary Dataset 2

Click here to access/download
Supplementary Material
Supplementary_Dataset_2_FTICR_16s.rar

Supplementary Dataset 3

Click here to access/download
Supplementary Material
Supplementarty_Datasets_3_List_of_theoretical_sulfatides.csv

Click here to access/download
Supplementary Material
Gigascience_QCL-MRMS_Suppl_REV final.pdf

edited version of original Supplement

Click here to access/download
Supplementary Material
Gigascience_QCL-MRMS_Suppl_REV_edited.docx

Supplementary Dataset 4

Click here to access/download
Supplementary Material
Supplementary_Dataset_4_extended_mz_list.xlsx

Click here to access/download
Supplementary Material
Supplementary_Dataset_5_Detailled_FDR_List.xlsx

revised manuscript with highlighted edits

Click here to access/download
Supplementary Material
Gigascience_QCL-MRMS_Main_REV
edited_final_new.docx

revised manuscript with highlighted edits

Click here to access/download
Supplementary Material
Gigascience_QCL-MRMS_Main_REV
edited_final__new.docx

Cover Letter

Click here to access/download;Personal Cover;Gigascience_QCL-MRMS_REV_Cover_Letter_final.pdf

Center for Mass Spectrometry and Optical Spectroscopy (CeMOS)

Carsten Hopf, PhD

Paul-Wittsack-Str. 10
68163 Mannheim, Germany
Tel. +49 621 292-6802
Fax. +49 621 292-6-6802-1
Email: c.hopf@hs-mannheim.de

To:

Yannan Fan

Editorial Office

Date: 29.10.2025

Submission of revised version of Gruber et al. to *GigaScience*

Dear Yannan Fan,

It is our pleasure to submit a revised version of our manuscript entitled **"A sulfatide-centered ultra-high resolution magnetic resonance MALDI imaging benchmark dataset for MS1-based lipid annotation tools"** for consideration as a Data Note in GigaScience.

We thank the Editor and the anonymous reviewers for their careful assessment of our manuscript, for their generally positive opinion on our manuscript and for the constructive feedback provided. We greatly appreciate the opportunity to revise and strengthen our work in light of these valuable comments. The reviewer input has identified key areas for clarification and enhancement that will substantially improve both the scientific rigor and clarity of our data note.

While the reviewers did not ask for additional experiments, they especially asked for more data, more clarity, more transparency and better documentation of manual data annotation. As a consequence, we have generated **additional supplementary datasets (No. 4 and 5)**, which we now include with the revised version. Here we provide an in-depth overview of mass accuracies and annotations across n=4 biological replicates. Moreover, we updated the manuscript by moving old supplementary figure 7 to **new figure 2b** to further strengthen our comparison of the annotations at the two different resolving powers. In line with the reviewers' comments, we also generated a **new supplementary figure 7** to demonstrate why some low abundant species still remain unannotated even at 50% FDR.

We provide detailed point-for-point responses to each reviewer comment, with corresponding manuscript revisions highlighted. We believe that these revisions directly address all concerns raised and bring the manuscript into alignment with GigaScience's standards for data transparency and reproducibility.

We submit an edited version of the revised manuscript and a clean one for clarity.

We hope you now find our Data Note suitable for publication in GigaScience. Thank you for your consideration.

Yours sincerely,

Carsten

Professor Carsten Hopf, PhD
Head, Center of Mass Spectrometry and Optical Spectroscopy (CeMOS)

Gruber et al. **GIGA-D-25-00298**

Point-for-Point Reply to Reviewers

A sulfatide-centered ultra-high resolution magnetic resonance MALDI imaging benchmark dataset for MS1-based lipid annotation tools

Revision 2
1. Editorial Comments 2
2. Reviewer #1 2
   2.1 Provision of the "Ground Truth" Annotation List: 3
   2.2 Strengthening the "Ground Truth" Justification: 3
   2.3 Deeper Analysis of Automated Annotation Discrepancies: 4
   2.4 Minor Revisions 5
   2.5 Closing Comment 7
3. Reviewer #2: 7
3.1 Comment 1 7
3.2 Comment 2 8
3.3 Comment 3 9
3.4 Comment 4 9
3.5 Comment 5 10
3.6 Final Comment 11

Revision

1. Editorial Comments

Your manuscript "A sulfatide-centered ultra-high resolution magnetic resonance MALDI imaging benchmark dataset for MS1-based lipid annotation tools" (GIGA-D-25-00298) has been assessed by our reviewers. Although it is of interest, we are unable to consider it for publication in its current form. The reviewers have raised a number of points which we believe would improve the manuscript and may allow a revised version to be published in GigaScience.

Their reports, together with any other comments, are below. Please also take a moment to check our website at https://www.editorialmanager.com/giga/ for any additional comments that were saved as attachments.

We thank the Editor and the anonymous reviewers for their careful assessment of our manuscript and for the constructive feedback provided. We greatly appreciate the opportunity to revise and strengthen our work in light of these valuable comments. The reviewer input has identified key areas for clarification and enhancement that will substantially improve both the scientific rigor and clarity of our data note.

Below, we provide detailed point-for-point responses to each reviewer comment, with corresponding manuscript revisions highlighted. We believe that these revisions directly address all concerns raised and bring the manuscript into alignment with GigaScience's standards for data transparency and reproducibility.

In addition, please register any new software application in the bio.tools and SciCrunch.org databases to receive RRID (Research Resource Identification Initiative ID) and biotoolsID identifiers, and include these in your manuscript. Computational workflows should be registered in workflowhub.eu and the DOIs cited in the relevant places in the manuscript. These will facilitate tracking, reproducibility and re-use of your tool.

Does not apply – as outlined in code availability statement.

All web links and URLs should be given a reference number and included in the reference list rather than within the text of the manuscript. Please remove the URLs, cite them as reference and adjust the order of the reference accordingly. Except for "Availability of Source Code and Requirements"

Two web links and URL were removed from the manuscript and moved to references as reference 30 and 37.

2. Reviewer #1

This manuscript by Gruber et al. provides a Data Note detailing a high-value, sulfatide-focused benchmark dataset for the mass spectrometry imaging (MSI) community. The project is well thought

out, technically advanced, and directly meets a major need for biologically relevant, deeply characterized ground-truth data to test MS1-level metabolite annotation software. It is a big technical achievement to create an ultra-high-resolution dataset (R ~1,230,000) with a 7T FT-ICR instrument. The use of isotopic fine structure (IFS) to boost annotation confidence is a major strength. Using QCL-MIR imaging strategically to guide the MSI acquisition is a smart and effective way to do things. It's great that the authors are committed to FAIR principles.

The writing in the manuscript is excellent, and the data is very good. It makes a big difference in the field. There are, however, several changes that should be made to make it clearer, more scientifically complete, and more useful as a stand-alone benchmark resource for the community. The following points are given to help make the manuscript stronger for publication.

We sincerely appreciate Reviewer 1's thoughtful and encouraging assessment of our work. Their recognition of the technical achievement and the potential impact of this benchmark dataset for the MSI community is invaluable, and their constructive suggestions for strengthening the manuscript will significantly improve its clarity and utility.

2.1 Provision of the “Ground Truth" Annotation List:

The benchmark dataset is the most important part of this Data Note. The manuscript's supplementary information, on the other hand, doesn't seem to have the final, curated list of manual annotations that make up the "ground truth." For this dataset to be truly reusable for benchmarking third-party software, it needs another table. This table should show all of the manually annotated sulfatides for each replicate, along with their experimental m/z, proposed sum formula, lipid annotation, mass error (ppm), and a way to tell if IFS was used to confirm them.

We thank Reviewer 1 for highlighting this critical gap. We agree that providing a comprehensive, curated ground-truth annotation table is essential for the utility of this dataset as a benchmark. We have added **new supplementary datasets 4 and 5.** Here, we included a list of all manually annotated sulfatides for each replicate, including the following columns: proposed sum formula, lipid annotation (including acyl chain composition), m/z measured, mass deviation (ppb), and an indicator denoting whether isotopic fine structure (IFS) was used to confirm the annotation (**new Supplementary Dataset 4**). In addition, we provide a list of all sulfatides we annotated in metaspace with our custom database and at which FDR they were annotated for each replicate (**new Supplementary Dataset 5**). These files will serve as the definitive reference for benchmarking third-party MS1-level annotation software and will be prominently linked in the main manuscript and data repository to ensure discoverability. We believe this addition will substantially enhance the reusability and scientific value of the dataset for the community.

2.2 Strengthening the "Ground Truth" Justification:

The manuscript depends on an earlier publication (Ref) to validate the sulfatide structures using MS/MS. It is acceptable to reference previous work, but a benchmark Data Note should be as self-sufficient as possible. Please add a short paragraph to the "Data Validation and Quality Control" section

that sums up the main MS/MS fragmentation evidence from Ref that backs up the sulfatide identifications. This will give users of the dataset a more complete and clear chain of evidence.

We thank Reviewer 1 for this important suggestion regarding data self-sufficiency. We agree that a benchmark Data Note should provide readers with a complete chain of evidence without requiring extensive reference to prior publications. We expanded the "Data Validation and Quality Control" section to include a concise summary of the key MS/MS fragmentation patterns and diagnostic ions that support the sulfatide identifications, referencing the earlier publication while distilling the most critical structural evidence. This addition will enable users of the dataset to understand the rationale behind our identifications directly within the manuscript and enhance the utility of the benchmark resource for the community.

2.3 Deeper Analysis of Automated Annotation Discrepancies:

The comparison with Metaspace shows how important this dataset is by showing that even a top-of-the-line tool can't annotate 14 high-confidence sulfatides. The discussion needs to be longer so that it can look at why these failures could be happening. Please explain why Metaspace's scoring algorithm, which only looks at the four most intense isotopic peaks, might not work well with this kind of ultra-high-resolution data where low-intensity IFS peaks (like 34 S) are very important. Talking about how future algorithms could make better use of this information would make the paper much more useful and help with the development of new tools.

We thank Reviewer 1 for this insightful suggestion. We expanded the Discussion section to provide a thorough analysis of the comparison between manual and automated (Metaspace) annotation, emphasizing that Metaspace performs robustly on this benchmark dataset and that the unannotated species represent a data limitation rather than an algorithmic limitation.

Metaspace performed well overall on this benchmark dataset. At a 10% FDR threshold, Metaspace successfully identified 43 of the 91 manually annotated sulfatides in the ultra-high-resolution dataset, demonstrating effective performance for species with complete or near-complete isotopic envelopes above noise thresholds. The algorithm's isotopic pattern matching and mass accuracy-based scoring framework functions reliably within its design specifications and represents a valuable resource for high-throughput MSI annotation workflows.

The 14 unannotated sulfatides represent inherent data limitations, not algorithmic deficiencies. These species are low-abundance compounds for which only the monoisotopic $[M-H]^-$ peak was detectable above the signal-to-noise threshold, while the isotopic envelope peaks fell below detection limits. This represents a fundamental measurement constraint: when the complete isotopic envelope is not experimentally resolvable due to low abundance, annotation based on isotopic pattern matching becomes physically impossible, regardless of algorithm sophistication. Metaspace appropriately rejects such species rather than making spurious assignments based on incomplete isotopic data—a conservative approach that prioritizes annotation confidence. Manual recovery of these species relied on monoisotopic m/z matching against the custom reference database combined with external validation (Gruber et al. 2025), exploiting orthogonal validation information unavailable to automated algorithms processing only MS1 data.

However, we identified one genuine algorithmic opportunity in the ultra-high-resolution regime. Notably, 37% of our manual annotations in the first replicate (34 of 91) and 40% in the second replicate (39 of 97) were substantiated by ^{34}S isotopic fine structure (IFS) peak detection. This demonstrates that diagnostic IFS information is frequently available in this dataset but underutilized by Metaspace, which restricts isotopic envelope matching to the four most intense peaks. While this limitation does not prevent successful annotation of species with complete isotopic envelopes, the inability to leverage IFS peaks for confidence stratification represents a missed opportunity: high-confidence annotations supported by robust IFS detection remain algorithmically indistinguishable from lower-confidence mass-error-based assignments.

We included a prospective discussion of algorithmic refinements that could enhance performance in ultra-high-resolution workflows, including: (a) expansion of isotopic peak matching to encompass all resolved peaks above instrument-specific signal-to-noise thresholds, enabling full exploitation of IFS information when present; (b) explicit confidence stratification that explicitly correlates annotation reliability with IFS detectability; (c) integration of machine-learning classifiers trained on empirical IFS signatures to improve discrimination between isomeric sulfatides; and (d) incorporation of complementary analytical dimensions (ion mobility, MS/MS fragmentation) where available. These additions position this dataset as a valuable training and validation resource for algorithm refinement without diminishing the demonstrated effectiveness of current-generation tools like Metaspace.

2.4 Minor Revisions

2.4.1 Clarification of Table 1:

The row headers for the R2 dataset ("all" vs. "QCL-MIR-guided") are slightly confusing, as all R2 data is QCL-MIR-guided. Please revise these for clarity (e.g., "Total Annotations in ROIs" and "Annotations with Confirmed IFS Evidence").

We thank Reviewer 1 for this important suggestion regarding table clarity. We revised the row labels for improved transparency and precision. The headers were changed from "all" and "QCL-MIR-guided" to "Total Annotations in ROIs" and "Annotations with Confirmed IFS Evidence," respectively. These revised labels explicitly communicate the distinction between the complete set of manually identified sulfatides within the regions of interest and the subset of those annotations that were specifically validated through isotopic fine structure peak detection. This revision eliminates ambiguity and enhances the interpretability of the benchmark dataset for end users.

2.4.2 Definition of "Internal Error":

The legend for Figure 1g should include a brief definition or reference for how "internal error" was calculated to ensure the metric is fully understood.

We thank Reviewer 1 for this suggestion to enhance clarity regarding the "internal error" metric in Figure 1g. We revised the figure legend to explicitly define the calculation method. The internal error was calculated based on Equation 13 from Roux et al., Eur. Phys. J. D (2013) 67:75 (https://link.springer.com/article/10.1140/epjd/e2013-40110-x), which quantifies the consistency of mass measurement accuracy of each identified species. We added the following text to the Figure 1g

legend: "Internal error was calculated according to Eq. 13 in Roux et al. [reference number], reflecting the deviation between observed and theoretical isotopic mass distributions." This addition ensures that readers can readily interpret the metric and reproduce the quality assessment independently.

2.4.3 Confirmation of Database Contents:

In the Methods section, please add a sentence explicitly confirming that all manually annotated sulfatide species were included in the custom database file used for the Metaspace analysis. This is a crucial detail for a fair comparison.

We thank Reviewer 1 for identifying this critical detail regarding methodological transparency. We agree that explicit confirmation of database completeness is essential for ensuring a fair and reproducible comparison between manual and algorithmic annotations. We added a clarifying sentence to the Methods section explicitly confirming that all 91 (first replicate) and 97 (second replicate) manually annotated sulfatide species were included in the custom database file (Supplementary Dataset 3) supplied to Metaspace for the analysis. This statement ensures that the comparison is conducted on equal footing and that Metaspace's apparent annotation failures cannot be attributed to missing entries in the reference database. This addition reinforces the validity of our findings regarding the algorithmic limitations of Metaspace and strengthens the benchmark value of our dataset.

2.4.4 Explicit Statement of Dataset Limitations:

In the "Re-use Potential" section, it would be beneficial to explicitly state the inherent trade-off of the ultra-high-resolution approach. Please add a sentence acknowledging that the dataset is optimized for high-confidence annotation and that this comes at the cost of reduced sensitivity and comprehensive spatial coverage compared to a standard MSI experiment.

We thank Reviewer 1 for this important suggestion regarding the transparency of dataset limitations. We agree that explicitly stating the inherent trade-offs of the ultra-high-resolution approach strengthens the manuscript and provides critical context for end users considering this dataset for their work. We added a clarifying statement to the "Re-use Potential" section that explicitly acknowledges the methodological trade-off: whilst the QCL-MIR-guided ultra-high-resolution approach achieves substantially elevated identification confidence through isotopic fine structure analysis and reduced ion current to minimize space charge effects, this optimization comes at the cost of reduced ion abundance and spatial coverage compared to conventional whole-tissue MSI experiments. Specifically, we noted that the number of sulfatide annotations in the QCL-MIR-guided ultra-high-resolution dataset (91 and 97 for the 60-week replicates) was lower than in the conventional non-guided whole-tissue approach (118 and 115), reflecting the intentional trade-off between analytical depth and comprehensive tissue coverage. This addition ensures that prospective users understand both the strengths and practical limitations of the dataset and can make informed decisions regarding its applicability to their specific research objectives.

2.4.5 Link to Custom Database:

The Methods section mentions the creation of a custom database of 780 theoretical sulfatides. Please explicitly state in the text that this database is available as Supplementary Dataset 3.

We thank Reviewer 1 for this suggestion to enhance the accessibility and traceability of the custom database. We agree that explicitly linking the database reference to its location in the supplementary materials strengthens reproducibility and facilitates access for users. We verified that the Methods section already contains explicit reference to the database availability as Supplementary Dataset 3 in the sentence: " A custom reference database integrating LipidMaps with 780 theoretically derived sulfatide structures, available as Supplementary Dataset 3, was constructed for Metaspace analysis." This statement ensures that readers can readily locate and access the custom database used for the analysis, supporting the reproducibility and transparency objectives of the study.

2.5 Closing Comment

Addressing these points will significantly enhance the manuscript's value and ensure its lasting impact as a key resource for the computational mass spectrometry community.

We appreciate Reviewer 1's constructive feedback, which has substantially strengthened the manuscript's scientific rigor, methodological transparency, and clarity regarding the benchmark dataset's utility. We are confident that these revisions address all concerns raised and position the manuscript as a robust, lasting resource for the computational mass spectrometry community.

3. Reviewer #2:

I believe that the dataset produced is a great contribution to the community. My major concerns are as follows:

We thank Reviewer 2 for the encouraging assessment of the dataset's contribution and for the detailed constructive feedback that has strengthened the manuscript. We address the major concerns below.

3.1 Comment 1

The data described is good but please clarify how would be solution the discrepancy between the manual annotations and the computational annotations and annotations quality for he sulfatide-centered MSI dataset, challenges?

We appreciate this question, which prompted us to clarify the discussion regarding discrepancies between manual and computational annotations. This concern aligns substantively with Reviewer #1's comment regarding the limitations of the Metaspace annotation algorithm (Comment 2.3 “Deeper Analysis of Automated Annotation Discrepancies”), and we have expanded the Discussion section comprehensively to address both perspectives.

The core challenges underlying the manual-computational divergence are fundamentally rooted in algorithmic design constraints and represent important considerations for future tool development. The primary discrepancy arises from Metaspace's scoring methodology, which restricts isotopic envelope matching to the four most intense peaks, thereby failing to exploit diagnostic isotopic fine

structure (IFS) information—particularly ^{34}S isotopologues—that was readily accessible in our manually curated dataset. This algorithmic limitation means that Metaspace cannot discriminate between structurally distinct sulfatides that exhibit identical or near-identical precursor m/z values but differ in their isotopic signatures. Conversely, our manual annotation workflow leveraged the complete isotopic fingerprint available at 1,230,000 mass resolving power, enabling unambiguous identification of species that computational algorithms currently cannot resolve.

Beyond algorithmic limitations, we identified three additional interrelated challenges: (1) signal intensity dependence, wherein low-abundance sulfatides exhibit attenuated IFS peak intensities that may fall below detection thresholds, rendering them inaccessible to IFS-based annotation strategies; (2) database completeness, which we addressed directly by ensuring all manually annotated species were represented in the custom database supplied to Metaspace; and (3) confidence stratification, wherein current algorithms provide no mechanism to distinguish high-confidence annotations supported by robust IFS detection from those inferred from precursor m/z alone.

To bridge this discrepancy, we propose that future annotation algorithms should implement the following innovations: (a) expansion of isotopic envelope matching to encompass all resolved peaks above instrument-specific signal-to-noise thresholds; (b) integration of machine-learning classifiers trained on empirical IFS signatures across heterogeneous signal intensity ranges; (c) explicit confidence scoring that correlates annotation reliability with IFS detectability; and (d) incorporation of complementary analytical dimensions (ion mobility, MS/MS fragmentation) to adjudicate between isotopically indistinguishable candidates.

In response to both reviewers' comments, we have substantially expanded the Discussion section to articulate these pathways for algorithmic advancement. This benchmark dataset provides the ground truth and diverse signal intensity distribution necessary to validate and refine such approaches, thereby directly addressing the manual-computational discrepancy through iterative algorithmic improvement.

3.2 Comment 2

Please remove too old references unless they are pioneer and replace with the new ones.

We appreciate this helpful suggestion regarding the currency and relevance of our reference list. We have carefully reviewed all cited literature and updated the manuscript to emphasize recent publications from 2020 onward, while selectively keeping foundational references that establish essential theoretical and methodological frameworks. Specifically, we removed outdated references that lack current relevance: Masselon et al. (2002) and Marshall & Hendrickson (2008) were removed because they addressed calibration and FT-ICR theory from the early 2000s and have been superseded by modern approaches. Kind & Fiehn (2007) was replaced with current metabolomic annotation strategies reflecting best practices today. Thompson et al. (2020), although focused on mass accuracy, used a FIA-CASI-FTMS workflow that is not directly applicable to our MALDI imaging context and was therefore replaced with Popov et al. (2014), which provides foundational work on isotopic fine structure analysis using dynamically harmonized FT-ICR cells—directly relevant to the ultra-high-resolution lipid characterization presented in this study.

Critically, we integrated Pieczonka et al. (2025) (10.1021/acs.analchem.5c00488) as a contemporary reference addressing modern FT-ICR harmonization and space charge mitigation strategies in ultra-high-resolution instruments. Additionally, we replaced Nikolaev et al. (2016) with Cochran et al. (2024)

(https://doi.org/10.3390/biomedicines12081786), a comprehensive review on FT-ICR-MS applications in metabolomics and theoretical frameworks.

Conversely, we retained seminal older references—such as Hess et al. (1996), which introduced the ARSA-deficient mouse model employed in this study, Shi et al. (1998) on the first demonstration of isotopic fine structure resolution in biological molecules, and Wong & Amster (2007) on fundamental space charge physics—because these papers establish foundational biological models, methodological innovations, and physical principles that remain essential references in contemporary FT-ICR and mass spectrometry literature and directly underpin the technical and biological innovations presented in this work. This balanced approach ensures that the manuscript reflects state-of-the-art developments whilst maintaining essential historical and theoretical context.

3.3 Comment 3

Please try to add some of figures as supplementary instead of text,

We appreciate this comment, though we note that the phrasing could be interpreted in multiple ways—specifically, whether the suggestion is to move main text figures to supplementary material or conversely to elevate supplementary figures to the main text for enhanced clarity. To provide the most beneficial revision, we interpret your comment as a request to move key supplementary figures into the main manuscript to improve readability and reinforce the manuscript narrative with visual support.

In response, we have reorganized the figure presentation to strengthen the main narrative. Specifically, the **old Supplementary Figure 7** (showing 77,000 R annotation results) is now integrated into the expanded **updated Figure 2b** alongside the 1,230,000 R data, providing direct visual comparison of annotation performance across both mass resolutions within the main text. Additionally and line with the first Reviewer (Comment 2.1) we created a new Supplementary Dataset 4 that provides comprehensive elaboration of mass accuracy metrics and isotopic fine structure (IFS) detection across all n=4 biological replicates, further strengthening the quantitative validation of our annotation quality assessment and demonstrating consistency across the full dataset. These reorganizations allow readers to immediately contextualize the superior performance of ultra-high mass resolving power and the robustness of our IFS-based validation strategy without requiring extensive supplementary material consultation, thereby enhancing manuscript clarity and data accessibility.

If your comment instead refers to relocating main text figures to supplementary material, we welcome further clarification so that we may optimize the figure arrangement accordingly. Our priority is to present this benchmark dataset in the most comprehensible format for the readership.

3.4 Comment 4

algorithm is not fully optimized or not?

We appreciate this question regarding algorithmic optimization. However, we note that the phrasing of this comment could be interpreted in multiple ways—specifically, whether the question pertains to the state of optimization of the Metaspace algorithm itself, the optimization of our custom database parameters for Metaspace analysis, or the optimization of our manual annotation workflow. To provide

the most comprehensive response, we interpret your comment as addressing the optimization status of the Metaspace algorithm for sulfatide annotation.

This topic was extensively addressed in our response to Reviewer #1, Comment 2.3 (Deeper Analysis of Automated Annotation Discrepancies), where we comprehensively analyzed Metaspace's performance on this benchmark dataset.

To summarize: Metaspace is well-optimized for its core design specifications, successfully identifying 43 of 91 manually annotated sulfatides at a 10% FDR threshold. The 14 unannotated species represent a measurement limitation—low-abundance compounds with incomplete isotopic envelopes where only the monoisotopic $[M-H]^-$ peak exceeds the noise threshold. This is fundamentally a data constraint, not an algorithmic deficiency. Metaspace's conservative approach of rejecting incomplete datasets is scientifically sound.

However, we identified one genuine optimization opportunity: expanding isotopic envelope matching beyond the four most intense peaks to exploit ^{34}S isotopic fine structure information for enhanced confidence stratification. Our data show that 37–40% of sulfatides exhibit resolvable IFS peaks, underscoring the potential of this signal for sulfatide discrimination. This refinement would strengthen performance without compromising the algorithm's established effectiveness.

3.5 Comment 5

How did you recover the missing annotations? Please clarify/elabroate this

We appreciate this question regarding the recovery of the 14 and 19 sulfatide species that remained unannotated by Metaspace, even at permissive FDR thresholds of 50%. These missing annotations represent low-abundance sulfatide species for which only the monoisotopic $[M-H]^-$ peak exhibited sufficient signal intensity, while parts of the isotopic envelope, particularly the diagnostic ^{34}S isotopologues, fell below the instrument signal-to-noise threshold.

Manual spectral inspection at ultra-high mass resolving power (1,230,000 for the MR-MSI dataset) enabled identification of these species through their monoisotopic m/z values combined with pattern matching against the custom sulfatide database and validation against the external reference dataset (Gruber et al. 2025, https://www.nature.com/articles/s41467-025-59839-3). However, because the isotopic envelope could not be resolved for these low-abundance species, the Metaspace algorithm, which requires isotopic peak matching for confidence scoring, could not recognize them algorithmically. This represents a fundamental trade-off: whilst Metaspace requires visible isotopic evidence for high-confidence annotation, low-abundance species often lack sufficient signal intensity to resolve their full isotopic signature, even at ultra-high mass resolving power.

The recovery of these marginal cases highlights a critical sensitivity limitation in automated annotation workflows and demonstrates why expert curation informed by validated reference databases remains essential for comprehensive sulfatide characterization in ultra-high-resolution MSI datasets.

3.6 Final Comment

Would be happy to review after revisions.

We thank Reviewer 2 for the encouraging assessment and constructive feedback. We have systematically addressed all major concerns through comprehensive revision of the Discussion section, careful curation of the reference list, and clarification of annotation methodologies. We look forward to resubmission of the revised manuscript for further consideration.
