# Supplementary material for: A sulfatide-centered ultra-high-resolution magnetic resonance MALDI imaging benchmark dataset for MS1-based lipid annotation tools
Source: Gigascience. 2025 Dec 9;14:giaf150. doi: 10.1093/gigascience/giaf150 (PMC12766628; doi:10.1093/gigascience/giaf150)
Supplement: giaf150_Authors_Response_To_Reviewer_Comments_Original_Submission [file giaf150_authors_response_to_reviewer_comments_original_submission.pdf]

Author's Response To Reviewer Comments

Gruber et al. GIGA-D-25-00298

Point-for-Point Reply to Reviewers

A sulfatide-centered ultra-high resolution magnetic resonance MALDI imaging benchmark dataset for MS1-based lipid annotation tools

Revision 2

1. Editorial Comments 2
2. Reviewer #1 2
2.1 Provision of the "Ground Truth" Annotation List: 3
2.2 Strengthening the "Ground Truth" Justification: 3
2.3 Deeper Analysis of Automated Annotation Discrepancies: 4
2.4 Minor Revisions 5
2.5 Closing Comment 7
3. Reviewer #2: 7
3.1 Comment 1 7
3.2 Comment 2 8
3.3 Comment 3 9
3.4 Comment 4 9
3.5 Comment 5 10
3.6 Final Comment 10

Revision

1. Editorial Comments
Your manuscript "A sulfatide-centered ultra-high resolution magnetic resonance MALDI imaging benchmark dataset for MS1-based lipid annotation tools" (GIGA-D-25-00298) has been assessed by our reviewers. Although it is of interest, we are unable to consider it for publication in its current form. The reviewers have raised a number of points which we believe would improve the manuscript and may allow a revised version to be published in GigaScience.

Their reports, together with any other comments, are below. Please also take a moment to check our website at https://www.editorialmanager.com/giga/ for any additional comments that were saved as attachments.

We thank the Editor and the anonymous reviewers for their careful assessment of our manuscript and for the constructive feedback provided. We greatly appreciate the opportunity to revise and strengthen our work in light of these valuable comments. The reviewer input has identified key areas for clarification and enhancement that will substantially improve both the scientific rigor and clarity of our data note.
Below, we provide detailed point-for-point responses to each reviewer comment, with corresponding manuscript revisions highlighted. We believe that these revisions directly address all concerns raised and bring the manuscript into alignment with GigaScience's standards for data transparency and reproducibility.

In addition, please register any new software application in the bio.tools and SciCrunch.org databases to receive RRID (Research Resource Identification Initiative ID) and biotoolsID identifiers, and include these in your manuscript. Computational workflows should be registered in workflowhub.eu and the DOIs cited in the relevant places in the manuscript. These will facilitate tracking, reproducibility and re-use of your tool.

Does not apply – as outlined in code availability statement.

All web links and URLs should be given a reference number and included in the reference list rather than within the text of the manuscript. Please remove the URLs, cite them as reference and adjust the order of the reference accordingly. Except for "Availability of Source Code and Requirements"

Two web links and URL were removed from the manuscript and moved to references as reference 30 and 37.

2. Reviewer #1
This manuscript by Gruber et al. provides a Data Note detailing a high-value, sulfatide-focused benchmark dataset for the mass spectrometry imaging (MSI) community. The project is well thought out, technically advanced, and directly meets a major need for biologically relevant, deeply characterized ground-truth data to test MS1-level metabolite annotation software. It is a big technical achievement to create an ultra-high-resolution dataset (R~1,230,000) with a 7T FT-ICR instrument. The use of isotopic fine structure (IFS) to boost annotation confidence is a major strength. Using QCL-MIR imaging strategically to guide the MSI acquisition is a smart and effective way to do things. It's great that the authors are committed to FAIR principles.
The writing in the manuscript is excellent, and the data is very good. It makes a big difference in the field. There are, however, several changes that should be made to make it clearer, more scientifically complete, and more useful as a stand-alone benchmark resource for the community. The following points are given to help make the manuscript stronger for publication.

We sincerely appreciate Reviewer 1's thoughtful and encouraging assessment of our work. Their recognition of the technical achievement and the potential impact of this benchmark dataset for the MSI community is invaluable, and their constructive suggestions for strengthening the manuscript will significantly improve its clarity and utility.

2.1 Provision of the “Ground Truth" Annotation List:
The benchmark dataset is the most important part of this Data Note. The manuscript's supplementary information, on the other hand, doesn't seem to have the final, curated list of manual annotations that make up the "ground truth." For this dataset to be truly reusable for benchmarking third-party software, it needs another table. This table should show all of the manually annotated sulfatides for each replicate, along with their experimental m/z, proposed sum formula, lipid annotation, mass error (ppm), and a way to tell if IFS was used to confirm them.

We thank Reviewer 1 for highlighting this critical gap. We agree that providing a comprehensive, curated ground-truth annotation table is essential for the utility of this dataset as a benchmark. We have added new supplementary datasets 4 and 5. Here, we included a list of all manually annotated sulfatides for each replicate, including the following columns: proposed sum formula, lipid annotation (including acyl chain composition), m/z measured, mass deviation (ppb), and an indicator denoting whether isotopic fine structure (IFS) was used to confirm the annotation (new Supplementary Dataset 4). In addition, we provide a list of all sulfatides we annotated in metaspace with our custom database and at which FDR they were annotated for each replicate (new Supplementary Dataset 5). These files will serve as the definitive reference for benchmarking third-party MS1-level annotation software and will be prominently linked in the main manuscript and data repository to ensure discoverability. We believe this addition will substantially enhance the reusability and scientific value of the dataset for the community.

2.2 Strengthening the "Ground Truth" Justification:
The manuscript depends on an earlier publication (Ref) to validate the sulfatide structures using MS/MS. It is acceptable to reference previous work, but a benchmark Data Note should be as self-sufficient as

possible. Please add a short paragraph to the "Data Validation and Quality Control" section that sums up the main MS/MS fragmentation evidence from Ref that backs up the sulfatide identifications. This will give users of the dataset a more complete and clear chain of evidence.

We thank Reviewer 1 for this important suggestion regarding data self-sufficiency. We agree that a benchmark Data Note should provide readers with a complete chain of evidence without requiring extensive reference to prior publications. We expanded the "Data Validation and Quality Control" section to include a concise summary of the key MS/MS fragmentation patterns and diagnostic ions that support the sulfatide identifications, referencing the earlier publication while distilling the most critical structural evidence. This addition will enable users of the dataset to understand the rationale behind our identifications directly within the manuscript and enhance the utility of the benchmark resource for the community.

2.3 Deeper Analysis of Automated Annotation Discrepancies:
The comparison with Metaspace shows how important this dataset is by showing that even a top-of-the-line tool can't annotate 14 high-confidence sulfatides. The discussion needs to be longer so that it can look at why these failures could be happening. Please explain why Metaspace's scoring algorithm, which only looks at the four most intense isotopic peaks, might not work well with this kind of ultra-high-resolution data where low-intensity IFS peaks (like 34 S) are very important. Talking about how future algorithms could make better use of this information would make the paper much more useful and help with the development of new tools.

We thank Reviewer 1 for this insightful suggestion. We expanded the Discussion section to provide a thorough analysis of the comparison between manual and automated (Metaspace) annotation, emphasizing that Metaspace performs robustly on this benchmark dataset and that the unannotated species represent a data limitation rather than an algorithmic limitation.
Metaspace performed well overall on this benchmark dataset. At a 10% FDR threshold, Metaspace successfully identified 43 of the 91 manually annotated sulfatides in the ultra-high-resolution dataset, demonstrating effective performance for species with complete or near-complete isotopic envelopes above noise thresholds. The algorithm's isotopic pattern matching and mass accuracy-based scoring framework functions reliably within its design specifications and represents a valuable resource for high-throughput MSI annotation workflows.
The 14 unannotated sulfatides represent inherent data limitations, not algorithmic deficiencies. These species are low-abundance compounds for which only the monoisotopic $[M-H]^-$ peak was detectable above the signal-to-noise threshold, while the isotopic envelope peaks fell below detection limits. This represents a fundamental measurement constraint: when the complete isotopic envelope is not experimentally resolvable due to low abundance, annotation based on isotopic pattern matching becomes physically impossible, regardless of algorithm sophistication. Metaspace appropriately rejects such species rather than making spurious assignments based on incomplete isotopic data—a conservative approach that prioritizes annotation confidence. Manual recovery of these species relied on monoisotopic m/z matching against the custom reference database combined with external validation (Gruber et al. 2025), exploiting orthogonal validation information unavailable to automated algorithms processing only MS1 data.
However, we identified one genuine algorithmic opportunity in the ultra-high-resolution regime. Notably, 37% of our manual annotations in the first replicate (34 of 91) and 40% in the second replicate (39 of 97) were substantiated by ^{34}S isotopic fine structure (IFS) peak detection. This demonstrates that diagnostic IFS information is frequently available in this dataset but underutilized by Metaspace, which restricts isotopic envelope matching to the four most intense peaks. While this limitation does not prevent successful annotation of species with complete isotopic envelopes, the inability to leverage IFS peaks for confidence stratification represents a missed opportunity: high-confidence annotations supported by robust IFS detection remain algorithmically indistinguishable from lower-confidence mass-error-based assignments.
We included a prospective discussion of algorithmic refinements that could enhance performance in ultra-high-resolution workflows, including: (a) expansion of isotopic peak matching to encompass all resolved peaks above instrument-specific signal-to-noise thresholds, enabling full exploitation of IFS information when present; (b) explicit confidence stratification that explicitly correlates annotation reliability with IFS detectability; (c) integration of machine-learning classifiers trained on empirical IFS signatures to improve discrimination between isomeric sulfatides; and (d) incorporation of complementary analytical dimensions

(ion mobility, MS/MS fragmentation) where available. These additions position this dataset as a valuable training and validation resource for algorithm refinement without diminishing the demonstrated effectiveness of current-generation tools like Metaspace.

2.4 Minor Revisions

2.4.1 Clarification of Table 1:
The row headers for the R2 dataset ("all" vs. "QCL-MIR-guided") are slightly confusing, as all R2 data is QCL-MIR-guided. Please revise these for clarity (e.g., "Total Annotations in ROIs" and "Annotations with Confirmed IFS Evidence").

We thank Reviewer 1 for this important suggestion regarding table clarity. We revised the row labels for improved transparency and precision. The headers were changed from "all" and "QCL-MIR-guided" to "Total Annotations in ROIs" and "Annotations with Confirmed IFS Evidence," respectively. These revised labels explicitly communicate the distinction between the complete set of manually identified sulfatides within the regions of interest and the subset of those annotations that were specifically validated through isotopic fine structure peak detection. This revision eliminates ambiguity and enhances the interpretability of the benchmark dataset for end users.

2.4.2 Definition of "Internal Error":
The legend for Figure 1g should include a brief definition or reference for how "internal error" was calculated to ensure the metric is fully understood.

We thank Reviewer 1 for this suggestion to enhance clarity regarding the "internal error" metric in Figure 1g. We revised the figure legend to explicitly define the calculation method. The internal error was calculated based on Equation 13 from Roux et al., Eur. Phys. J. D (2013) 67:75 (https://link.springer.com/article/10.1140/epjd/e2013-40110-x), which quantifies the consistency of mass measurement accuracy of each identified species. We added the following text to the Figure 1g legend: "Internal error was calculated according to Eq. 13 in Roux et al. [reference number], reflecting the deviation between observed and theoretical isotopic mass distributions." This addition ensures that readers can readily interpret the metric and reproduce the quality assessment independently.

2.4.3 Confirmation of Database Contents:
In the Methods section, please add a sentence explicitly confirming that all manually annotated sulfatide species were included in the custom database file used for the Metaspace analysis. This is a crucial detail for a fair comparison.

We thank Reviewer 1 for identifying this critical detail regarding methodological transparency. We agree that explicit confirmation of database completeness is essential for ensuring a fair and reproducible comparison between manual and algorithmic annotations. We added a clarifying sentence to the Methods section explicitly confirming that all 91 (first replicate) and 97 (second replicate) manually annotated sulfatide species were included in the custom database file (Supplementary Dataset 3) supplied to Metaspace for the analysis. This statement ensures that the comparison is conducted on equal footing and that Metaspace's apparent annotation failures cannot be attributed to missing entries in the reference database. This addition reinforces the validity of our findings regarding the algorithmic limitations of Metaspace and strengthens the benchmark value of our dataset.
2.4.4 Explicit Statement of Dataset Limitations:
In the "Re-use Potential" section, it would be beneficial to explicitly state the inherent trade-off of the ultra-high-resolution approach. Please add a sentence acknowledging that the dataset is optimized for high-confidence annotation and that this comes at the cost of reduced sensitivity and comprehensive spatial coverage compared to a standard MSI experiment.

We thank Reviewer 1 for this important suggestion regarding the transparency of dataset limitations. We agree that explicitly stating the inherent trade-offs of the ultra-high-resolution approach strengthens the manuscript and provides critical context for end users considering this dataset for their work. We added a clarifying statement to the "Re-use Potential" section that explicitly acknowledges the methodological trade-off: whilst the QCL-MIR-guided ultra-high-resolution approach achieves substantially elevated

identification confidence through isotopic fine structure analysis and reduced ion current to minimize space charge effects, this optimization comes at the cost of reduced ion abundance and spatial coverage compared to conventional whole-tissue MSI experiments. Specifically, we noted that the number of sulfatide annotations in the QCL-MIR-guided ultra-high-resolution dataset (91 and 97 for the 60-week replicates) was lower than in the conventional non-guided whole-tissue approach (118 and 115), reflecting the intentional trade-off between analytical depth and comprehensive tissue coverage. This addition ensures that prospective users understand both the strengths and practical limitations of the dataset and can make informed decisions regarding its applicability to their specific research objectives.

2.4.5 Link to Custom Database:
The Methods section mentions the creation of a custom database of 780 theoretical sulfatides. Please explicitly state in the text that this database is available as Supplementary Dataset 3.

We thank Reviewer 1 for this suggestion to enhance the accessibility and traceability of the custom database. We agree that explicitly linking the database reference to its location in the supplementary materials strengthens reproducibility and facilitates access for users. We verified that the Methods section already contains explicit reference to the database availability as Supplementary Dataset 3 in the sentence: " A custom reference database integrating LipidMaps with 780 theoretically derived sulfatide structures, available as Supplementary Dataset 3, was constructed for Metaspace analysis." This statement ensures that readers can readily locate and access the custom database used for the analysis, supporting the reproducibility and transparency objectives of the study.

2.5 Closing Comment
Addressing these points will significantly enhance the manuscript's value and ensure its lasting impact as a key resource for the computational mass spectrometry community.

We appreciate Reviewer 1's constructive feedback, which has substantially strengthened the manuscript's scientific rigor, methodological transparency, and clarity regarding the benchmark dataset's utility. We are confident that these revisions address all concerns raised and position the manuscript as a robust, lasting resource for the computational mass spectrometry community.

3. Reviewer #2:
I believe that the dataset produced is a great contribution to the community. My major concerns are as follows:
We thank Reviewer 2 for the encouraging assessment of the dataset's contribution and for the detailed constructive feedback that has strengthened the manuscript. We address the major concerns below.

3.1 Comment 1
The data described is good but please clarify how would be solution the discrepancy between the manual annotations and the computational annotations and annotations quality for he sulfatide-centered MSI dataset, challenges?
We appreciate this question, which prompted us to clarify the discussion regarding discrepancies between manual and computational annotations. This concern aligns substantively with Reviewer #1's comment regarding the limitations of the Metaspace annotation algorithm (Comment 2.3 “Deeper Analysis of Automated Annotation Discrepancies”), and we have expanded the Discussion section comprehensively to address both perspectives.
The core challenges underlying the manual-computational divergence are fundamentally rooted in algorithmic design constraints and represent important considerations for future tool development. The primary discrepancy arises from Metaspace's scoring methodology, which restricts isotopic envelope matching to the four most intense peaks, thereby failing to exploit diagnostic isotopic fine structure (IFS) information—particularly ^{34}S isotopologues—that was readily accessible in our manually curated dataset. This algorithmic limitation means that Metaspace cannot discriminate between structurally distinct sulfatides that exhibit identical or near-identical precursor m/z values but differ in their isotopic signatures. Conversely, our manual annotation workflow leveraged the complete isotopic fingerprint available at 1,230,000 mass resolving power, enabling unambiguous identification of species that computational algorithms currently cannot resolve.

Beyond algorithmic limitations, we identified three additional interrelated challenges: (1) signal intensity dependence, wherein low-abundance sulfatides exhibit attenuated IFS peak intensities that may fall below detection thresholds, rendering them inaccessible to IFS-based annotation strategies; (2) database completeness, which we addressed directly by ensuring all manually annotated species were represented in the custom database supplied to Metaspace; and (3) confidence stratification, wherein current algorithms provide no mechanism to distinguish high-confidence annotations supported by robust IFS detection from those inferred from precursor m/z alone.

To bridge this discrepancy, we propose that future annotation algorithms should implement the following innovations: (a) expansion of isotopic envelope matching to encompass all resolved peaks above instrument-specific signal-to-noise thresholds; (b) integration of machine-learning classifiers trained on empirical IFS signatures across heterogeneous signal intensity ranges; (c) explicit confidence scoring that correlates annotation reliability with IFS detectability; and (d) incorporation of complementary analytical dimensions (ion mobility, MS/MS fragmentation) to adjudicate between isotopically indistinguishable candidates.

In response to both reviewers' comments, we have substantially expanded the Discussion section to articulate these pathways for algorithmic advancement. This benchmark dataset provides the ground truth and diverse signal intensity distribution necessary to validate and refine such approaches, thereby directly addressing the manual-computational discrepancy through iterative algorithmic improvement.

3.2 Comment 2

Please remove too old references unless they are pioneer and replace with the new ones.

We appreciate this helpful suggestion regarding the currency and relevance of our reference list. We have carefully reviewed all cited literature and updated the manuscript to emphasize recent publications from 2020 onward, while selectively keeping foundational references that establish essential theoretical and methodological frameworks. Specifically, we removed outdated references that lack current relevance: Masselon et al. (2002) and Marshall & Hendrickson (2008) were removed because they addressed calibration and FT-ICR theory from the early 2000s and have been superseded by modern approaches. Kind & Fiehn (2007) was replaced with current metabolomic annotation strategies reflecting best practices today. Thompson et al. (2020), although focused on mass accuracy, used a FIA-CASI-FTMS workflow that is not directly applicable to our MALDI imaging context and was therefore replaced with Popov et al. (2014), which provides foundational work on isotopic fine structure analysis using dynamically harmonized FT-ICR cells—directly relevant to the ultra-high-resolution lipid characterization presented in this study. Critically, we integrated Pieczonka et al. (2025) (10.1021/acs.analchem.5c00488) as a contemporary reference addressing modern FT-ICR harmonization and space charge mitigation strategies in ultra-high-resolution instruments. Additionally, we replaced Nikolaev et al. (2016) with Cochran et al. (2024) (https://doi.org/10.3390/biomedicines12081786), a comprehensive review on FT-ICR-MS applications in metabolomics and theoretical frameworks.

Conversely, we retained seminal older references—such as Hess et al. (1996), which introduced the ARSA-deficient mouse model employed in this study, Shi et al. (1998) on the first demonstration of isotopic fine structure resolution in biological molecules, and Wong & Amster (2007) on fundamental space charge physics—because these papers establish foundational biological models, methodological innovations, and physical principles that remain essential references in contemporary FT-ICR and mass spectrometry literature and directly underpin the technical and biological innovations presented in this work. This balanced approach ensures that the manuscript reflects state-of-the-art developments whilst maintaining essential historical and theoretical context.

3.3 Comment 3

Please try to add some of figures as supplementary instead of text,

We appreciate this comment, though we note that the phrasing could be interpreted in multiple ways—specifically, whether the suggestion is to move main text figures to supplementary material or conversely to elevate supplementary figures to the main text for enhanced clarity. To provide the most beneficial revision, we interpret your comment as a request to move key supplementary figures into the main manuscript to improve readability and reinforce the manuscript narrative with visual support.

In response, we have reorganized the figure presentation to strengthen the main narrative. Specifically, the old Supplementary Figure 7 (showing 77,000 R annotation results) is now integrated into the expanded

updated Figure 2b alongside the 1,230,000 R data, providing direct visual comparison of annotation performance across both mass resolutions within the main text. Additionally and line with the first Reviewer (Comment 2.1) we created a new Supplementary Dataset 4 that provides comprehensive elaboration of mass accuracy metrics and isotopic fine structure (IFS) detection across all n=4 biological replicates, further strengthening the quantitative validation of our annotation quality assessment and demonstrating consistency across the full dataset. These reorganizations allow readers to immediately contextualize the superior performance of ultra-high mass resolving power and the robustness of our IFS-based validation strategy without requiring extensive supplementary material consultation, thereby enhancing manuscript clarity and data accessibility.
If your comment instead refers to relocating main text figures to supplementary material, we welcome further clarification so that we may optimize the figure arrangement accordingly. Our priority is to present this benchmark dataset in the most comprehensible format for the readership.

3.4 Comment 4
algorithm is not fully optimized or not?

We appreciate this question regarding algorithmic optimization. However, we note that the phrasing of this comment could be interpreted in multiple ways—specifically, whether the question pertains to the state of optimization of the Metaspace algorithm itself, the optimization of our custom database parameters for Metaspace analysis, or the optimization of our manual annotation workflow. To provide the most comprehensive response, we interpret your comment as addressing the optimization status of the Metaspace algorithm for sulfatide annotation.
This topic was extensively addressed in our response to Reviewer #1, Comment 2.3 (Deeper Analysis of Automated Annotation Discrepancies), where we comprehensively analyzed Metaspace's performance on this benchmark dataset.
To summarize: Metaspace is well-optimized for its core design specifications, successfully identifying 43 of 91 manually annotated sulfatides at a 10% FDR threshold. The 14 unannotated species represent a measurement limitation—low-abundance compounds with incomplete isotopic envelopes where only the monoisotopic $[M-H]^-$ peak exceeds the noise threshold. This is fundamentally a data constraint, not an algorithmic deficiency. Metaspace's conservative approach of rejecting incomplete datasets is scientifically sound.
However, we identified one genuine optimization opportunity: expanding isotopic envelope matching beyond the four most intense peaks to exploit ^{34}S isotopic fine structure information for enhanced confidence stratification. Our data show that 37–40% of sulfatides exhibit resolvable IFS peaks, underscoring the potential of this signal for sulfatide discrimination. This refinement would strengthen performance without compromising the algorithm's established effectiveness.

3.5 Comment 5
How did you recover the missing annotations? Please clarify/elabroate this

We appreciate this question regarding the recovery of the 14 and 19 sulfatide species that remained unannotated by Metaspace, even at permissive FDR thresholds of 50%. These missing annotations represent low-abundance sulfatide species for which only the monoisotopic $[M-H]^-$ peak exhibited sufficient signal intensity, while parts of the isotopic envelope, particularly the diagnostic ^{34}S isotopologues, fell below the instrument signal-to-noise threshold.
Manual spectral inspection at ultra-high mass resolving power (1,230,000 for the MR-MSI dataset) enabled identification of these species through their monoisotopic m/z values combined with pattern matching against the custom sulfatide database and validation against the external reference dataset (Gruber et al. 2025, https://www.nature.com/articles/s41467-025-59839-3). However, because the isotopic envelope could not be resolved for these low-abundance species, the Metaspace algorithm, which requires isotopic peak matching for confidence scoring, could not recognize them algorithmically. This represents a fundamental trade-off: whilst Metaspace requires visible isotopic evidence for high-confidence annotation, low-abundance species often lack sufficient signal intensity to resolve their full isotopic signature, even at ultra-high mass resolving power.
The recovery of these marginal cases highlights a critical sensitivity limitation in automated annotation workflows and demonstrates why expert curation informed by validated reference databases remains essential for comprehensive sulfatide characterization in ultra-high-resolution MSI datasets.

3.6 Final Comment
Would be happy to review after revisions.
We thank Reviewer 2 for the encouraging assessment and constructive feedback. We have systematically addressed all major concerns through comprehensive revision of the Discussion section, careful curation of the reference list, and clarification of annotation methodologies. We look forward to resubmission of the revised manuscript for further consideration.

Editorial Manager®

Cookie Preference Center

We use cookies which are necessary to make our site work. We may also use additional cookies to analyze, improve and personalize our content and your digital experience. For more information, see our Cookie Policy.

You may choose not to allow some types of cookies. However, blocking some types may impact your experience of our site and the services we are able to offer. See the different category headings below to find out more or change your settings.

Allow all

Manage Consent Preferences

Strictly Necessary Cookies

Always active

These cookies are necessary for the website to function and cannot be switched off in our systems. They are usually only set in response to actions made by you which amount to a request for services, such as setting your privacy preferences, logging in or filling in forms. You can set your browser to block or alert you about these cookies, but some parts of the site will not then work.

Functional Cookies

These cookies enable the website to provide enhanced functionality and personalisation. They may be set by us or by third party providers whose services we have added to our pages. If you do not allow these cookies then some or all of these services may not function properly.

Performance Cookies

These cookies allow us to count visits and traffic sources so we can measure and improve the performance of our site. They help us to know which pages are the most and least popular and see how visitors move around the site.

Targeting Cookies

These cookies may be set through our site by our advertising partners. They may be used by those companies to build a profile of your interests and show you relevant adverts on other sites. If you do not allow these cookies, you will experience less targeted advertising.

Cookie List

Clear

Apply Cancel

Consent Leg.Interest

Confirm my choices

Powered by onetrust
