# Supplementary material for: A sulfatide-centered ultra-high-resolution magnetic resonance MALDI imaging benchmark dataset for MS1-based lipid annotation tools
Source: Gigascience. 2025 Dec 9;14:giaf150. doi: 10.1093/gigascience/giaf150 (PMC12766628; doi:10.1093/gigascience/giaf150)
Supplement: giaf150_Reviewer_1_Report_Original_Submission — Morteza Akbari, Ph.D. -- 9/22/2025 [file giaf150_reviewer_1_report_original_submission.pdf]

Reviewer Report

Title: A sulfatide-centered ultra-high resolution magnetic resonance MALDI imaging benchmark dataset for MS1-based lipid annotation tools

Version: Original Submission **Date:** 9/22/2025

Reviewer name: Morteza Akbari, Ph.D.

Reviewer Comments to Author:

This manuscript by Gruber et al. provides a Data Note detailing a high-value, sulfatide-focused benchmark dataset for the mass spectrometry imaging (MSI) community. The project is well thought out, technically advanced, and directly meets a major need for biologically relevant, deeply characterized ground-truth data to test MS1-level metabolite annotation software. It is a big technical achievement to create an ultra-high-resolution dataset (Râˆ¼1,230,000) with a 7T FT-ICR instrument. The use of isotopic fine structure (IFS) to boost annotation confidence is a major strength. Using QCL-MIR imaging strategically to guide the MSI acquisition is a smart and effective way to do things. It's great that the authors are committed to FAIR principles.The writing in the manuscript is excellent, and the data is very good. It makes a big difference in the field. There are, however, several changes that should be made to make it clearer, more scientifically complete, and more useful as a stand-alone benchmark resource for the community. The following points are given to help make the manuscript stronger for publication.Major Revisions1. Provision of the "Ground Truth" Annotation List:The benchmark dataset is the most important part of this Data Note. The manuscript's supplementary information, on the other hand, doesn't seem to have the final, curated list of manual annotations that make up the "ground truth." For this dataset to be truly reusable for benchmarking third-party software, it needs another table. This table should show all of the manually annotated sulfatides for each replicate, along with their experimental m/z, proposed sum formula, lipid annotation, mass error (ppm), and a way to tell if IFS was used to confirm them.2. Strengthening the "Ground Truth" Justification:The manuscript depends on an earlier publication (Ref) to validate the sulfatide structures using MS/MS. It is acceptable to reference previous work, but a benchmark Data Note should be as self-sufficient as possible. Please add a short paragraph to the "Data Validation and Quality Control" section that sums up the main MS/MS fragmentation evidence from Ref that backs up the sulfatide identifications. This will give users of the dataset a more complete and clear chain of evidence.3. Deeper Analysis of Automated Annotation Discrepancies:The comparison with Metaspace shows how important this dataset is by showing that even a top-of-the-line tool can't annotate 14 high-confidence sulfatides. The discussion needs to be longer so that it can look atwhy these failures could be happening. Please explain why Metaspace's scoring algorithm, which only looks at the four most intense isotopic peaks, might not work well with this kind of ultra-high-resolution data where low-intensity IFS peaks (like 34 S) are very important. Talking about how future algorithms could make better use of this information would make the paper much more useful and help with the development of new tools.Minor Revisions1. Clarification of Table 1: The row headers for the R2 dataset ("all" vs. "QCL-MIR-guided") are slightly confusing, as all R2 data is QCL-MIR-guided. Please revise these for clarity (e.g., "Total Annotations in ROIs" and "Annotations with

Confirmed IFS Evidence"). 2. Definition of "Internal Error": The legend for Figure 1g should include a brief definition or reference for how "internal error" was calculated to ensure the metric is fully understood. 3. Confirmation of Database Contents: In the Methods section, please add a sentence explicitly confirming that all manually annotated sulfatide species were included in the custom database file used for the Metaspace analysis. This is a crucial detail for a fair comparison. 4. Explicit Statement of Dataset Limitations: In the "Re-use Potential" section, it would be beneficial to explicitly state the inherent trade-off of the ultra-high-resolution approach. Please add a sentence acknowledging that the dataset is optimized for high-confidence annotation and that this comes at the cost of reduced sensitivity and comprehensive spatial coverage compared to a standard MSI experiment. 5. Link to Custom Database: The Methods section mentions the creation of a custom database of 780 theoretical sulfatides. Please explicitly state in the text that this database is available as Supplementary Dataset 3. Addressing these points will significantly enhance the manuscript's value and ensure its lasting impact as a key resource for the computational mass spectrometry community.

Level of Interest

Please indicate how interesting you found the manuscript: Choose an item.

Quality of Written English

Please indicate the quality of language in the manuscript: Choose an item.

Declaration of Competing Interests

Please complete a declaration of competing interests, considering the following questions:

- Have you in the past five years received reimbursements, fees, funding, or salary from an organisation that may in any way gain or lose financially from the publication of this manuscript, either now or in the future?
- Do you hold any stocks or shares in an organisation that may in any way gain or lose financially from the publication of this manuscript, either now or in the future?
- Do you hold or are you currently applying for any patents relating to the content of the manuscript?
- Have you received reimbursements, fees, funding, or salary from an organization that holds or has applied for patents relating to the content of the manuscript?
- Do you have any other financial competing interests?
- Do you have any non-financial competing interests in relation to this paper?

If you can answer no to all of the above, write 'I declare that I have no competing interests' below. If your reply is yes to any, please give details below.

I have no competing interests

I agree to the open peer review policy of the journal. I understand that my name will be included on my report to the authors and, if the manuscript is accepted for publication, my named report including any attachments I upload will be posted on the website along with the authors' responses. I agree for my report to be made available under an Open Access Creative Commons CC-BY license (http://creativecommons.org/licenses/by/4.0/). I understand that any comments which I do not wish to be included in my named report can be included as confidential comments to the editors, which will not be published.

Choose an item.

To further support our reviewers, we have joined with Publons, where you can gain additional credit to further highlight your hard work (see: https://publons.com/journal/530/gigascience). On publication of this paper, your review will be automatically added to Publons, you can then choose whether or not to claim your Publons credit. I understand this statement.

Yes Choose an item.
